# Can you trust your reconstructed lineage tree? A homoplasy-based approach for irreversible evolution

**DOI:** 10.1101/2025.07.27.667007

**Published:** 2026-07-05

**Authors:** Pini Zilber, Sebastian Prillo, Yaara Neumeier, Nir Yosef, Boaz Nadler

**Affiliations:** * Department of Computer Science and Applied Mathematics, Weizmann Institute of Science, Israel.; † Department of Electrical Engineering and Computer Sciences, University of California, Berkeley, USA.; ‡ Department of Systems Immunology, Weizmann Institute of Science, Israel.

**Keywords:** CRISPR-Cas9 lineage tracing, homoplasy, phylogeny, irreversible evolution, accuracy evaluation, lineage tree reconstruction

## Abstract

Phylogeny inference is a fundamental problem in computational biology, with many proposed algorithms. Emerging techniques that couple single-cell genomics with Cas9-based genome editing open the way for in-depth analysis of cell phylogenies that underlie processes of clonal expansion, selection and diversification, from embryogenesis to cancer. A key distinguishing feature of cell lineage analysis with these techniques is the non-modifiability of Cas9-induced mutations, which motivates revisiting questions in phylogenetics. In this work, we ask one such fundamental question: is it possible to assess the reliability of an inferred lineage tree, even though we do not know its underlying ground truth? We present a homoplasy-based approach for this question that leverages the non-modifiability property. We show via simulations that under a broad range of settings, our method can effectively distinguish accurate reconstructions out of a pool of candidate solutions. Importantly, our homoplasy-based score is substantially more powerful than the commonly used parsimony score - a result that we back by both empirical and theoretical analysis. The computation of the homoplasy score is simple and scalable, thus opening the way for more rigorous analysis of cell lineages.

## 1 Introduction

Reconstruction of phylogenies has long stood as a cornerstone of evolutionary biology, with numerous methods developed over the years (Hennig, 1999; Nei and Kumar, 2000; Hall, 2004; Wiley and Lieberman, 2011). In the classical setting, the objective is to reconstruct the tree of life. The leaves of the tree represent extant species, and the internal nodes provide a way to reason about their latent ancestors far back in evolutionary history. Phylogenetics has also been used to formalize the analysis of sequences of nucleic or amino acids and shed light on the evolutionary forces that shaped them. The process of natural evolution of sequences, captured by classical models such as Jukes-Cantor, reflects the chances of mutations to occur and is assumed to be *modifiable*. Namely, the occurrence of a mutation in a site does not necessarily nullify the chances of that site being mutated again.

The absence of this property is one of the major distinguishing features of an emerging branch of phylogenetics, where trees depict cell lineages, with leaves corresponding to individual sampled cells, and internal nodes reflecting their ancestral cell division relationships (Gong et al., 2021). While cell lineages can be inferred based on somatic mutations or other natural and modifiable forms of heritable variation, the most powerful techniques (in terms of numbers of cells and the resolution of lineage trees) are based on inducible mutations at synthetic DNA elements (Kester and Van Oudenaarden, 2018; McKenna and Gagnon, 2019; Yang et al., 2022). In these applications, the heritable information that guides the retrospective inference of lineages is accrued through genome editing (e.g. with CRISPR/Cas9) of synthetic ”recorder sites” that are integrated into the genome (usually at the 3’ end of synthetic genes). Since CRISPR/Cas9 often has a much lower affinity to the edited sites, it is highly unlikely for additional mutations to occur in already-mutated sites, making this process *non-modifiable* (Sashittal et al., 2023).

Several studies investigated this new setting and proposed suitable algorithms to reconstruct cell lineages based on the mutation profiles accumulated in each cell (Jones et al., 2020; Feng et al., 2021; Seidel and Stadler, 2022; Wang et al., 2023; Sashittal et al., 2023), see also Gong et al. (2021) for a broad performance comparison. One strategy relies on the long-studied Camin-Sokal model (Camin and Sokal, 1965). This model assumes the less restrictive scenario of *irreversibility*, which permits mutated sites to undergo further mutations, but not to revert to an ancestral state. This is a notable difference from non-modifiability, except for the special case of a two-state evolution, where the two become identical. We note that in practical applications, other algorithms, including those designed for the classical problem such as neighbor-joining (Saitou and Nei, 1987), are often effective (Sugino and Lee, 2019; Jones et al., 2020; Prillo et al., 2025).

This multitude of strategies and algorithms for the inference problem raises the following question: given several reconstructions of the cell lineage tree that are all based on the same (non-modifiable) data, which reconstruction is more accurate? (i.e. more similar to the true one). More fundamentally, can we assess whether any individual reconstructed topology is accurate or not? This latter objective extends beyond simply ranking candidate topologies since it also aims to determine if any of the candidate topologies, e.g. the top-ranked ones, are close enough to the true topology and can be used for downstream analysis.

A common strategy to evaluate candidate solutions assumes that the true topology is known and compares directly to it (for example, using the Robinson-Foulds distance or the proportion of correct triplets (Jones et al., 2020; Gong et al., 2021)). While this strategy is naturally restricted, other approaches have been proposed that do not assume knowledge of the ground truth, including comparison of parsimony (the minimal number of ancestral mutation events that explain the data), or likelihood (given an evolutionary model of mutation accrual). These approaches, however, are often used only for ranking solutions and not for making ”absolute” decisions of whether or not a given tree is similar to the latent ground truth. Moreover, due to the limited sequence length in cell lineage tracing experiments, parsimony and likelihood scores may be poor indicators of topological accuracy. Indeed, given typical lineage tracing data, the reconstructed tree after contracting mutationless branches typically contain polytomies. Refining them may produce a collection of binary trees all with the same parsimony score. The phenomenon of multiple distinct trees with identical quality is known as phylogenetic terraces (Sanderson, McMahon, and Steel, 2011; Sanderson, McMahon, Stamatakis, et al., 2015). From a broader perspective, there has been significant efforts on assessing the robustness and reliability of reconstructed trees and their parameters. Particular emphasis has been made on branch support, deriving bootstrap or Bayesian measures for the reliability of specific clades in the reconstructed tree, see Simon (2022) for a review. For non-modifiable cell lineage tree reconstruction, Seidel and Stadler (2022) and Zwaans et al. (2025) developed TiDeTree, a Bayesian framework to estimate both the tree as well as various global parameters, such as mutation rates and transition probabilities. Given a collection of multiple reconstructed trees, a different approach to possibly construct a more accurate tree and to assess the branch support is to consider a consensus tree and compare the various trees to it. For non-modifiable cell lineage tracing, both Gong et al. (2021) and Dai and Molloy (2026) have shown that consensus trees may be more accurate. Finally, another approach to assess the relative reliability of different tree reconstructions is to use external additional knowledge such as gene expression or migration data, see for example Sashittal et al. (2023). Yet, despite this extensive body of work, we are not aware of methods that can effectively distinguish accurate from inaccurate tree reconstructions.

In this work, we present the *pairwise homoplasy score (PHS)* - a simple and theoretically grounded approach to address these problems. Leveraging the non-modifiable nature of the evolution process, our algorithm calculates a sequence of tail probabilities - one for each pair of leaves in the reconstructed tree - and combines them into a single score. We demonstrate that compared to parsimony and likelihood, the PHS approach is substantially more powerful in distinguishing accurate tree reconstructions out of a pool of candidate solutions. Furthermore, we present a theoretical analysis that provides insight into the advantage of PHS over parsimony.

The procedure for calculating the PHS has polynomial-time complexity, and can be easily run on trees with thousands of leaves.^1^ We expect it to become an important part of future pipelines for phylogenetic analysis in non-modifiable systems, and particularly CRISPR/Cas9-based tracing of cell lineages.

## 2 Problem Setup

We first briefly describe a typical cell lineage tracing experiment, and then the mathematical model and assumptions made in our manuscript. A typical experiment starts with a single progenitor cell that has k target sites along its genome, which are all unedited (i.e., yet unmutated). As the experiment ensues, the resulting progeny grows through cell divisions, with possible divergence from neutral growth due to advantageous properties of some clades. This evolutionary process can be described by a binary tree, where nodes correspond to cells. Each node u has a birth time $\tau_{u}$ and a sequence $s^{\left(u\right)}$ of k characters, which are the Cas9-induced mutations at its k sites. The root node of the tree, denoted by r, corresponds to the progenitor cell. It has a birth time $\tau_{r}=0$ and an unmutated sequence $s^{r}=(0,0,\ldots ,0)$. At the end of the experiment, the sequences at a subset of n cells are observed. Without loss Table 1: Model parameters. of generality, we rescale time so the end of the experiment is at τ=1. The n observed cells induce an underlying ground truth binary tree, denoted $T_{\text{GT}}$, which describes their clonal history. For future use, we denote the set of all node sequences in this tree by $S_{\text{GT}}=\left\{s^{\left(u\right)}:u\;\text{is a node in}\;T_{\text{GT}}\right\}$, and by $S_{n}$ the n×k matrix of the observed sequences at the n cells. By definition, $S_{n}$ is the subset of $S_{\text{GT}}$ that corresponds to the terminal leaves of $T_{\text{GT}}$. Finally, in the tree $T_{\text{GT}}$, the path length $\tau_{u,v}$ between a node u and a descendant node v is defined as the difference between their birth times, $\tau_{u,v}=\tau_{v}-\tau_{u}$.

In the main text, we assume for simplicity that the sequences at the n sampled cells are fully observed, namely, all entries of the matrix $S_{n}$ are observed. Settings with missing data, due to either stochastic or heritable missingness, are addressed in Appendix B. Given the n observed sequences $S_{n}$, standard problems are to reconstruct either the underlying tree topology $T_{\text{GT}}$ or the full tree $T_{\text{GT}}S_{\text{GT}}$. In contrast, we focus on assessing whether a given reconstructed tree is accurate and close to $T_{\text{GT}}$. To this end, in the next subsection we describe a commonly assumed model for CRISPR-Cas9 cell lineage data, and in Section 2.2 we mathematically formulate our problem.

### 2.1 CRISPR-Cas9 non-modifiable mutation model

We consider the following probabilistic model for a CRISPR-Cas9 cell lineage tracing experiment, similar to Wang et al. (2023), Sashittal et al. (2023), and Dai and Molloy (2026), among others. The first assumption is that mutations at different target sites occur independently along the tree. Specifically, each unmutated character i∈[k] (namely with $s_{i}=0$) evolves according to a Poisson process with a mutation rate λ. For simplicity, this rate λ is assumed fixed for all character locations in the tree; some extensions are discussed in Section 3. Once a mutation occurs, the mutated state is drawn from a probability distribution ${\left\{q_{j}\right\}}_{j=1}^{m}$ over a set of m possible integer states 1,…,m, where m=1 corresponds to the binary case. The evolution is *non-modifiable*: once a character is mutated, i.e. it has a nonzero state $s_{i}>0$, it cannot mutate again and its state remains fixed. Furthermore, all mutations of a cell are passed on to all its descendants.

In practice, this property is a design feature of CRISPR-Cas9 lineage recorders. An indel at a target site disrupts the sequence recognized by the guide RNA, so the site is not re-cut and the edit is not reverted, and is inherited unchanged by all descendant cells (Yang et al., 2022; Chan et al., 2019).

There are two key quantities that characterize the non-modifiable process described above. One is the probability ρ that at the end of the experiment an observed character is mutated. It is given by

(1) $$\rho =1-e^{-\lambda }.$$

The second quantity is the collision probability, denoted by q. This is the probability that two independent mutation events result in the same mutated state, and it is given by

(2) $$q=\sum_{j=1}^{m}q_{j}^{2}.$$

In general, the collision probability satisfies q≤1, with q=1 if and only if states are binary (m=1).

The parameters characterizing the tree topology and the generative process for the sequences are summarized in Table 1. We conclude this subsection with the following definition that will be used extensively in the manuscript.

#### Definition 1

(full tree). A full tree TS is a structure that consists of both a tree topology T, all of its branch lengths (elapsed time between divisions), and a collection of the character sequences S at all its tree nodes.

### 2.2 Distinguishing between accurate and inaccurate reconstructed trees

As mentioned in the introduction, several reconstruction algorithms were developed for non-modifiable CRISPR-Cas9 evolution models, for example Jones et al. (2020), Feng et al. (2021), Seidel and Stadler (2022), Wang et al. (2023), and Sashittal et al. (2023). The celebrated Neighbor-Joining method (Saitou and Nei, 1987), originally developed for reversible evolution models, was shown to perform well also in non-modifiable settings (Sugino and Lee, 2019; Jones et al., 2020; Prillo et al., 2025).

Given the input sequences $S_{n}$, with a limited sequence length k, it may be impossible to exactly reconstruct the ground-truth tree. Different reconstruction algorithms often output different tree topologies. Some of these reconstructed trees may be accurate, whereas others may be quite distant from the ground-truth tree. The problem at the focus of our work is to develop a method to decide whether a given reconstructed tree is accurate, or far from the ground-truth tree. Crucially, this task needs to be accomplished with the ground-truth tree being unknown. Our method exploits the non-modifiability of CRISPR-Cas9 mutations to calculate the probability of shared mutations across a lineage (homoplasies), thereby providing a robust statistical test for the accuracy of reconstructed trees.

In the phylogenetics literature, several works considered a different, though related problem of proposing meaningful distances between a reconstructed tree T and a known ground truth $T_{\text{GT}}$, such as the Robinson-Foulds (RF) distance (Robinson and Foulds, 1981) and the triplets score (Jones et al., 2020). These measures are useful in simulation studies, but not for assessing tree reconstructions in practical settings, where $T_{\text{GT}}$ is unknown.

Two common measures that do not require knowledge of $T_{\text{GT}}$ are the parsimony and the likelihood of a tree. In the parsimony approach, a simple polynomial-time procedure is applied to a reconstructed topology T to find the minimal number of ancestral mutations that give rise to the observed leaf states (Gusfield, 1997); see Appendix C. In contrast, the likelihood approach considers all possible combinations of ancestral states. Under a Markov assumption, this score can be calculated efficiently, while further requiring a model of mutation accrual and knowledge of edge lengths. According to the maximum parsimony principle, trees with a smaller number of mutations M(TS) are considered closer to the ground-truth one (Fitch, 1971; Albert, 2005). Similarly, trees with higher likelihood values are considered more accurate. Likelihood is more statistically grounded than parsimony: Under the assumption of a generative model of evolution, trees with maximum likelihood L(TS) are statistically consistent (Felsenstein, 1981; Warnow, 2018).

In principle, a set of reconstructed trees can be ordered by their parsimony or likelihood scores. This ordering is useful if the goal is to evaluate how different methods successfully optimized a given parsimony- or likelihood-based objective function. However, success in optimization does not always translate to meaningful differences in topological accuracy (e.g., as measured by RF distance). Furthermore, even if we evaluate trees solely by parsimony or likelihood rather than by topological accuracy, relying on their induced ordering can be misleading. As these scores do not provide an absolute measure of quality, ordering the reconstructed trees may be meaningless if their parsimony or likelihood scores are all very far from that of the ground truth. We empirically illustrate these issues in Section 4.

A fundamental challenge is thus to devise a score that is able to detect if a given tree is accurate or not, for example indicate if its normalized RF distance from the unknown ground-truth tree is smaller than some prescribed value ϵ. We mathematically formulate this challenge as follows: Given a tree TS, a distance measure d and a threshold ϵ∈(0,1), we consider the following hypothesis testing problem,

(3) $$H_{0}:d\left(TS,T_{\text{GT}}S_{\text{GT}}\right)\leq \epsilon \;\text{vs.}\;H_{1}:d\left(TS,T_{\text{GT}}S_{\text{GT}}\right)>\epsilon .$$

We emphasize that the main obstacle is that $T_{\text{GT}}S_{\text{GT}}$, and thus $d\left(TS,T_{\text{GT}}S_{\text{GT}}\right)$, are unknown. Note that in (3), the null hypothesis is the desirable one, in which the candidate tree is close to the ground-truth one. In this paper, we consider the following four distance functions:

- the normalized RF distance
(4) $$d_{\text{RF}}\left(TS,T_{\text{GT}}S_{\text{GT}}\right)=\mathrm{RF}\left(T,T_{\text{GT}}\right)/(2(n-3)),$$
- the triplets distance
(5) $$d_{\text{tri}}\left(TS,T_{\text{GT}}S_{\text{GT}}\right)=1-\mathrm{tri}\left(T,T_{\text{GT}}\right),$$
- a parsimony-based distance
(6) $$d_{\text{P}}\left(TS,T_{\text{GT}}S_{\text{GT}}\right)=\max\left\{0,\frac{M(TS)}{M\left(T_{\text{GT}}S_{\text{GT}}\right)}-1\right\},$$
- and a likelihood-based distance,
(7) $$d_{\text{L}}\left(TS,T_{\text{GT}}S_{\text{GT}}\right)=\max\left\{0,\frac{\log L\left(T_{\text{GT}}S_{\text{GT}}\right)}{\log L(TS)}-1\right\}.$$
  The max operator in Eqs. (6) and (7) ensures that these distances are non-negative. This is required as, given observed data $S_{n}$, the ground-truth tree $T_{\text{GT}}S_{\text{GT}}$ might not be the one with maximum parsimony or highest likelihood.

Our main contribution is an approach to resolve (3), which is applicable and powerful for a wide choice of distance functions between trees and of cutoff values ϵ. Specifically, we propose a homoplasy-based test statistic that quantifies the *consistency* of the candidate tree with respect to the observed sequences $S_{n}$ and the non-modifiability of the CRISPR-Cas9 model. As we show, our approach can distinguish accurate from inaccurate trees, under all four distance functions $d_{\text{RF}}$, $d_{\text{tri}}$, $d_{\text{P}}$ and $d_{\text{L}}$. In Section 4 we present simulation results for $d_{\text{RF}}$ and $d_{\text{P}}$; simulations for $d_{\text{tri}}$ and $d_{\text{L}}$ appear in the appendix. In addition, in Section 5 we provide theoretical support for our approach.

#### Remark 1.

There is a fundamental difference between the problem defined in Eq. (3) and classical statistical hypothesis testing. The latter is often formulated as a decision problem, whether observed data was generated from an assumed statistical model (the null) or an alternative one. In this work, in contrast, we assume that the CRISPR-Cas9 statistical model is correct. Instead, we test the consistency of the ”data” TS with the CRISPR-Cas9 model. Here, the ”data” includes both the observed sequences and the reconstructed tree and its inner sequences.

## 3 A Pairwise Homoplasy Approach

To describe our approach for the hypothesis testing in Eq. (3), we first recall the definition of two classical concepts in tree phylogeny: latest (i.e. lowest or most recent) common ancestor (LCA) and homoplasy.

### Definition 2 (LCA).

The latest common ancestor of a pair of leaves u, v with respect to a tree topology T is the most recent node (with latest birth time τ) whose descendants include both u and v, denoted LCA (u,v).

### Definition 3 (homoplasy and PHS).

For a pair of leaves u and v, there is a homoplasy in their i-th character with respect to a full tree TS if both leaves have the same mutation, $s_{i}^{\left(u\right)}=s_{i}^{\left(v\right)}\neq 0$, but their latest common ancestor was unmutated, $s_{i}^{\left(\text{LCA}(u,v)\right)}=0$. We denote the indicator of such a homoplasy in the i-th character of u and v by

(8) $${\mathrm{phs}}_{i}(u,v)=\left\{\begin{array}{ll} 1, & s_{i}^{\left(u\right)}=s_{i}^{\left(v\right)}\neq 0\;\text{and}\;s_{i}^{\left(\mathrm{LCA}(u,v)\right)}=0,\\ 0, & \text{otherwise,} \end{array}\right.$$

and by phs(u,v) the *pairwise homoplasy score* for the pair of leaves u, v with respect to a full tree TS, defined as their mean PHS over the k characters,

(9) $$\mathrm{phs}(u,v)=\frac{1}{k}\sum_{i=1}^{k}{\mathrm{phs}}_{i}(u,v).$$

For example, consider a full tree TS with $s^{\left(u\right)}=10221$, $s^{\left(v\right)}=13321$, and $s^{\left(\text{LCA}(\text{u},\text{v})\right)}=00020$. Then ${\mathrm{phs}}_{1}(u,v)={\mathrm{phs}}_{5}(u,v)=1$, while ${\mathrm{phs}}_{2}(u,v)={\mathrm{phs}}_{3}(u,v)={\mathrm{phs}}_{4}(u,v)=0$, and thus phs(u,v)=2/5.

Note that to compute the PHS, the unobserved sequence $s^{\left(w\right)}$ at the inner node w=LCA(u,v) needs to be known. In cases where a reconstruction algorithm provides us only with a tree topology T but not with its estimated inner sequences S, we first impute the ancestral states using a simple procedure of post-order traversal (Appendix A.2). Given two sister nodes, the state of their parent can in most cases be inferred without ambiguity due to non-modifiability. The one exception is when the two sister nodes are mutated and have the same state. In this case, we make the parsimonious choice and set the parent cell to the same state as its child nodes.

### 3.1 PHS probability distribution

Let $T_{\text{GT}}$ be a fixed ground-truth topology, and suppose its sequences $S_{\text{GT}}$ are generated according to the non-modifiable mutation model of Section 2.1. As the sequences are random, so are the PHS values of all pairs of tree leaves. Our test statistic is based on the tail probabilities of these values. To construct our test statistic, we shall make use of the following lemma, which describes the PHS probability distribution. Its proof is in Appendix E.

**Algorithm 1:** Computation of the PHS test statistic

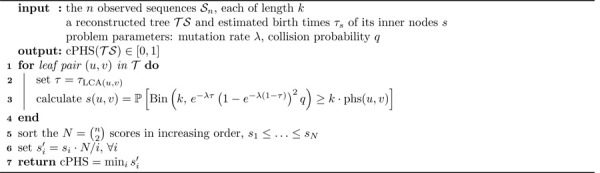

#### Lemma 1.

*Let* u *and* v *be a pair of leaves in* $T_{GT}$. *Denote their LCA by* w=LCA(u,v) *and its birth time by* $\tau_{w}\in [0,1)$. *Let* $\alpha_{\tau_{w}}=e^{-\lambda \tau_{w}}$ *and* $\beta_{\tau_{w}}=1-e^{-\lambda \left(1-\tau_{w}\right)}$, *where* λ *is the mutation rate. Then*, *for sequences of length* k *generated as in Section 2.1 with collision probability* q *(see Eq. (2))*,

(10) $$k\cdot phs(u,v)\sim Bin\left(k,\alpha_{\tau_{w}}\beta_{\tau_{w}}^{2}q\right).$$

Lemma 1 reveals two appealing properties of the PHS distribution. First, in terms of the model parameters, it depends on the mutation rate λ, sequence length k and the collision probability q, but not on the individual mutation probabilities $q_{j}$. Second, for any pair of leaves u, v, the PHS distribution does not depend on the full structure of the tree, but only on a single sufficient statistic: the birth time of their LCA. This simplifies the computation of our proposed test statistic (described in the next section), and facilitates its theoretical analysis (see Section 5).

### 3.2 A PHS-based test statistic

We are now ready to present our proposed PHS test statistic for the hypothesis problem in (3). For simplicity, in this section we assume the model parameters λ and q, as well as the birth times of all inner nodes of T, are known. As described in Appendix A.1, in our simulations we estimate λ and the birth times from the data. The collision probability q is assumed to be known also in simulations since, as detailed in Appendix A.1, q can be accurately estimated in various experimental settings. Moreover, as illustrated in Figure S5 (Appendix K), our test statistic is not sensitive to the exact value of q, and taking q=1/m, which corresponds to a uniform mutation distribution $q_{j}=1/m$, works well.

The calculation of our test statistic is outlined in Alg. 1. Given a candidate tree TS, it consists of two steps.

#### Step 1 (calculating tail probabilities).

Based on Lemma 1, for each pair of leaves (u,v) with latest common ancestor w=LCA(u,v) in the reconstructed tree T, we compute the tail probability of its PHS value as if this tree were the ground-truth one,

(11) $$s(u,v)=\mathbb{P}\left[\mathrm{Bin}\left(k,\alpha_{\tau_{w}}\beta_{\tau_{w}}^{2}q\right)\geq k\cdot \mathrm{phs}(u,v)\right].$$

Each score s(u,v) can be viewed as a p-value (i.e., right-tail probability under $H_{0}$) for the consistency of the observed sequences at the nodes u and v with the reconstructed tree. Since there are n leaves, Eq. (11) provides us with $N=\left(\begin{array}{c} n\\ 2 \end{array}\right)$ scores. The next step is to fuse these multiple p-values into a single score.

#### Step 2 (fusing the multiple scores).

Motivated by multiple hypothesis testing procedures, we sort the N scores in increasing order, $s_{1}\leq s_{2}\leq \ldots \leq s_{N}$, and focus on the first few smallest ones. Similar to the Benjamini-Hochberg (BH) procedure (Benjamini and Hochberg, 1995) (see Remark 3 below for more details), for each i≤N we compute an adjusted score

(12) $$s_{i}^{\prime }=s_{i}\cdot \frac{N}{i}.$$

Finally, our test statistic is the minimal adjusted score,

(13) $$\text{cPHS}(TS)=\underset{i}{\min}\;s_{i}^{\prime }.$$

Note that cPHS(TS)≤1, since $s_{N}^{\prime }=s_{N}\leq 1$. Given a reconstructed tree TS, we reject $H_{0}$ if cPHS(TS) is below some threshold t<1, and accept it otherwise.

##### Remark 2.

Let us provide some motivation and context for the above procedure. Often, some parts of the reconstructed tree are close to the ground truth. By construction, the corresponding scores s(u,v) in those parts are distributed approximately uniformly over [0, 1]. In contrast, other parts of the reconstructed tree may be less accurate. In those cases, some of the scores s(u,v) are expected to be closer to zero. Typically, the number of s(u,v) values that are close to zero is relatively small w.r.t. N≫1. Hence, our setting is similar to high-dimensional multiple hypothesis testing problems, whereby only a few out of many hypotheses deviate from the null.

##### Remark 3 (Relation to the BH procedure).

As described in Benjamini, Heller, and Yekutieli (2009, Section 2(b)), the BH procedure can be presented in terms of adjusted p-values, defined as

$$s_{i}^{\prime \prime }=\min\left\{\underset{j\geq i}{\min}\left\{s_{j}^{\prime }\right\},1\right\}.$$

Under this formulation, all hypotheses i for which $s_{i}^{\prime \prime }<t$ are rejected. In our case, we do not aim to reject or accept individual hypotheses, corresponding to individual p-values $s_{i}^{\prime \prime }$. Instead, we adopt a more stringent criterion: if even a single adjusted score $s_{i}^{\prime \prime }$ falls below the threshold, the entire tree is rejected. Accordingly, our test statistic coincides with the minimum BH-adjusted p-value:

$$\text{cPHS}(TS)=s_{1}^{\prime \prime }.$$

**Figure 1:**
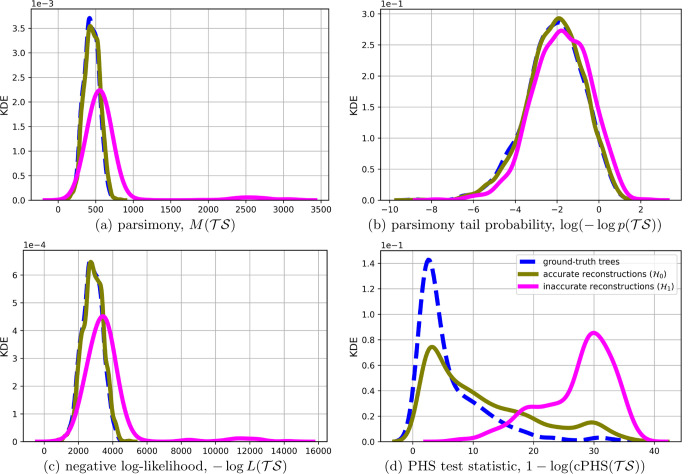
Kernel density estimates for four accuracy measures: parsimony (upper left); parsimony tail-probability (upper right); likelihood (bottom left); and our proposed cPHS (bottom right). The parsimony tail probability and cPHS test statistics are log-transformed for clarity of view. The hypotheses $H_{0}$ and $H_{1}$ are defined by (3) with $d=d_{\text{RF}}$ (4) and ϵ=1/4. The simulations were performed using Cassiopeia (Jones et al., 2020) as follows: binary tres with 10^6^ terminal cells were generated, from which $n=10^{3}$ observed cells were subsampled (subsampling ratio of 10^−3^). The sequence length is k=30, each site has m=32 possible mutations with probabilities $q_{j}$ drawn from an exponential distribution, and λ=−log2, so that there is ρ=50% probability to observe a mutation at a leaf. These parameter values are similar to those of experimental settings, see Jones et al. (2020).

As illustrated in Section 4 and in Appendix K below (see e.g. Figure 1(d)), cPHS is highly powerful in distinguishing between accurate and inaccurate trees. Furthermore, as cPHS separates accurate and inaccurate reconstructed trees by a large margin (see Figure 1), it does not require a delicate tuning of the threshold t. In practice, as illustrated in Section 4, a threshold value that depends only on the cutoff value ϵ of Eq. (3) works well for a wide range of model parameters (such as the sequence length k, the number of mutation states m, etc.).

##### Remark 4 (Non-uniform mutation rate).

Lemma 1, and thus Eq. (11), assume that the mutation rate λ is the same for all k characters. However, as long as characters are assumed to evolve independently, our PHS approach can be easily extended to the case of non-uniform mutation rates; see Claim 1 in Appendix E.

#### Complexity and runtime.

Given a reconstructed tree TS, computing its cPHS value can be done in a polynomial number of operations. Specifically, as the algorithm passes over all leaf pairs for each character, its time complexity is $O\left(kn^{2}\right)$. With our Python implementation, calculating cPHS for a tree with n=1000 leaves and sequences of length k=50 takes approximately two minutes on a standard PC.

#### Missing data.

In practical settings, some entries of $S_{n}$ may be missing, due to either limited sensitivity (the so-called stochastic drop out) or heritable mechanisms (e.g. concomitant resection of two adjacent target sites (Jones et al., 2020)). As described in Appendix B, our PHS approach can be easily extended to handle missing data, and empirically, it continues to perform well also in such cases.

## 4 Simulations

We demonstrate through a comprehensive set of simulations the capability of the cPHS test statistic (13) to resolve the hypothesis testing problem outlined in (3). We compare cPHS with three test statistics: (i) the parsimony of the reconstructed tree, M(TS); (ii) the negative log-likelihood of the reconstructed tree, L(TS); and (iii) the parsimony tail-probability, $p(TS)=\sum_{M\geq M(TS)}\mathbb{P}[M|T]$. The last test statistic is based on the parsimony probability distribution *conditional* on the specific reconstructed tree topology T, denoted as ℙ[M∣T]. As described in Appendix D, this distribution can be computed analytically.

For each simulation in this section, we used Cassiopeia (Jones et al., 2020) to generate 1000 ground-truth trees and their inner sequences, $T_{\text{GT}}S_{\text{GT}}$. Similar to other studies (Jones et al., 2020; Feng et al., 2021; Seidel and Stadler, 2022; Sashittal et al., 2023), each ground-truth tree topology was randomly generated according to a birth-death process. The rates of birth and death are not homogeneous throughout the tree but instead depend on a heritable, sub-clonal fitness level; see Appendix J. Given a tree topology, its inner sequences were generated according to the non-modifiable CRISPR-Cas9 mutation model described in Section 2.1. The considered settings are similar to those in Sashittal et al. (2023): sequence length of k=30, m=32 possible mutations, and n=200 cells. In this section, the matrix $S_{n}$ is fully observed; simulations with missing data appear in Appendix B. The observed data $S_{n}$ from each ground-truth tree was given as input to seven reconstruction algorithms: Neighbor-Joining (Saitou and Nei, 1987), Cassiopeia-Greedy (Jones et al., 2020), Shared-Mutation-Joining (Wang et al., 2023), MaxCut and MaxCut-Greedy (Snir and Rao, 2006; Jones et al., 2020), Spectral and Spectral-Greedy solvers (Jones et al., 2020). The seven reconstructed trees were then classified as either *accurate* or *inaccurate* according to (3), using normalized $\mathrm{RF}\;\left(d=d_{\text{RF}}\right)$ and parsimony $\left(d=d_{\text{P}}\right)$ as distance functions. Results for the other two distance functions, triplets $\left(d=d_{\text{tri}}\right)$ and likelihood $\left(d=d_{\text{L}}\right)$, are provided in Appendix K. We remark that in general, the reconstructed binary tree, as well as the ground-truth one, may contain edges that are not supported by any mutations. Similar to Sashittal et al. (2023) and Dai and Molloy (2026), we first contract mutationless branches, and compute the distance functions on the contracted trees. In particular, if two original binary trees disagreed only on mutationless branches, after this procedure, they are viewed as topologically equivalent, and their RF distance is zero.

### 4.1 Separability Capabilities of cPHS

To motivate our approach, we first demonstrate that given a reconstructed tree TS corresponding to an unknown ground truth $T_{\text{GT}}S_{\text{GT}}$, existing measures are unable to accurately distinguish between the null and the alternative hypotheses in (3). In this simulation, we considered a tree as inaccurate if $d_{\text{RF}}\left(TS,T_{\text{GT}}S_{\text{GT}}\right)>1/4$. This corresponds to the hypothesis testing problem in Eq. (3) with distance function $d=d_{\text{RF}}$ and ϵ=1/4; results for the other three distances d appear in Appendix K.

As shown in panels (a-c) of Fig. 1, for each of the three baseline test statistics there is substantial overlap between their values on accurate and inaccurate reconstructed trees. A similar overlap exists even between ground-truth and inaccurate reconstructed trees. This substantial overlap implies that *regardless of the chosen threshold* t, these measures cannot accurately separate ground-truth trees, or accurate trees, from inaccurate ones. In Figures S7 and S8 of Appendix K, we show that even when accuracy is defined by the *parsimony*-based distance $d_{\text{P}}$ (rather than the normalized RF), both the parsimony score and its tail-probability measure are ineffective in distinguishing between accurate and inaccurate reconstructed trees.

In contrast, Fig. 1(d) shows that our proposed PHS-based test statistic, described in Section 3, achieves a high separation between candidate trees with low (good) and high (bad) RF distances. Furthermore, it achieves a *nearly perfect* separation between ground-truth trees and candidate trees with high RF distance. In the next subsection and Appendix K we show that a similar conclusion holds for the other distances $d_{\text{RF}}$, $d_{\text{tri}}$, and $d_{\text{L}}$. Specifically, for each of these other distance functions, there is significant overlap in the distribution based on the parsimony or likelihood measures. In contrast, our PHS-based test statistic achieves a high separability. Notably, as demonstrated in Appendix K, cPHS is a better accuracy measure than parsimony even if the distance measure is parsimony itself, and similarly for likelihood. That is, if our goal is to tell whether the parsimony/likelihood of the candidate tree is close to the ground-truth parsimony/likelihood, it is more useful to look at its cPHS than at its parsimony/likelihood.

In Section 5 we provide theoretical support for the empirical advantage of our homoplasy-based approach over parsimony. Specifically, we prove that to detect certain incorrect topologies, parsimony requires exponentially longer sequences (in tree depth) compared to our PHS approach.

### 4.2 Classification Accuracy

The previous section demonstrated the separability capabilities of cPHS in comparison to other test statistics. In practice, however, we aim to classify reconstructed trees as either accurate or inaccurate by applying a threshold to these test statistics. In this section, we evaluate the classification performance of each baseline test statistic by assessing its ability to classify correctly if a reconstructed tree is accurate or not. Given a distance function and a cutoff value ϵ, for any tree denote by y∈{accurate, inaccurate} its true label according to (3). Similarly, denote by ${\hat{y}}_{t}$ the label estimated by some test statistic with a threshold t. For example, for cPHS,

$${\hat{y}}_{t}=\left\{\begin{array}{ll} \text{accurate,} & \text{cPHS}\geq t,\\ \text{inaccurate,} & \text{cPHS}<t. \end{array}\right.$$

We first examine the performance of the four test statistics across all possible thresholds, then focus on specific choices of t. In the first simulation, we compute the true positive rate (TPR) and false positive rate (FPR) of each test statistic as a function of the threshold t. These rates are defined as follows:

$$\mathrm{TPR}(t)=\mathbb{P}\left[{\hat{y}}_{t}=y|y=\mathrm{accurate}\right]\;\text{and}\;\mathrm{FPR}(t)=\mathbb{P}\left[{\hat{y}}_{t}\neq y|y=\mathrm{accurate}\right].$$

**Figure 2:**
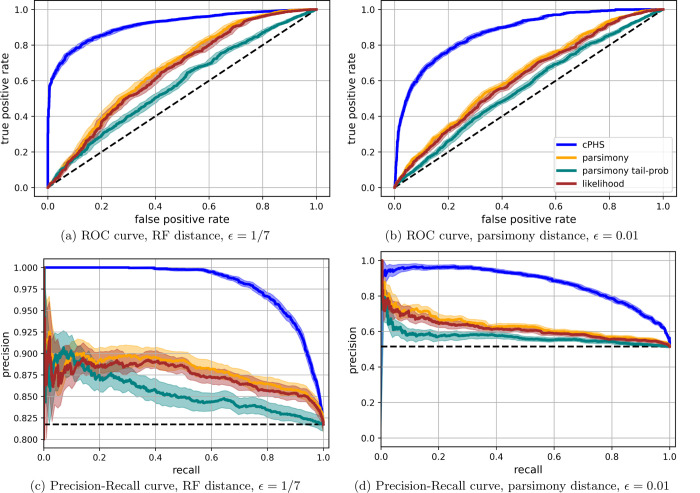
ROC (top) and Precision-Recall (bottom) curves of several test statistics for the hypothesis testing in (3). Each point on the curve corresponds to a different threshold t. The difference between the left and right panels is the distance measure in use: normalized RF with ϵ=1/7 (left) and parsimony distance with ϵ=0.01 (right). Dashed lines represent the expected performance of random guessing. The rates are calculated over 1000 realizations.

Plotting the TPR and FPR for all threshold values t gives ROC curves, presented in Fig. 2, with 95% confidence intervals computed via bootstrap. Panel (a) displays the results for $d=d_{\text{RF}}$ with ϵ=1/7, and panel (b) displays the results for $d=d_{\text{P}}$ with ϵ=0.01. It is apparent that, *regardless* of the selected threshold t, the performance of cPHS is superior to that of the other test statistics. Notably, the ROC curve of cPHS exhibits a steep ascent on the left side, indicating that cPHS achieves a satisfactory TPR even at low FPR levels.

Panels (c) and (d) of Fig. 2 present Precision-Recall curves. These metrics are defined as follows:

$$\mathrm{Precision}(t)=\mathbb{P}\left[{\hat{y}}_{t}=y|{\hat{y}}_{t}=\mathrm{accurate}\right]\;\text{and}\;\mathrm{Recall}(t)=\mathrm{TPR}(t).$$

A clear distinction in performance between cPHS and the other test statistics is evident under these metrics as well. Specifically, when the hypotheses in (3) involve the RF distance $\left(d=d_{\text{RF}}\right)$, as seen in panel (c), cPHS achieves a high Precision of 0.93 at an equally high Recall.

**Figure 3:**
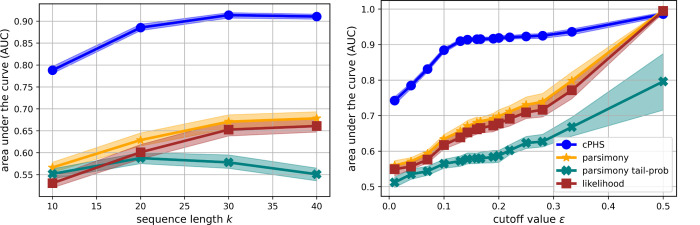
AUC values for the ROC curves of several test statistics for the hypothesis testing in (3) with $d=d_{\text{RF}}$. Left: ϵ=1/7 is fixed and the sequence length k is varying. Right: k=30 is fixed and ϵ is varying. Each point is based on 1000 realizations of the simulation.

The simulations above were performed with sequence length k=30 and number of mutated states m=32. Next, we examined the performance as these parameters are varied. Figure 3 shows the AUC (area under the ROC curve) as a function of the sequence length k (left panel) and of the number of mutated states m (right panel). Additional results for varying values of m, n and ρ (bijectively related to λ, see (1)) can be found in Appendix K, as well as results for the AUPRC (area under the Precision-Recall curve). As shown, under a wide range of settings, cPHS consistently outperforms the other test statistics by a significant margin.

We now turn to results obtained for specific choices of the threshold t. Our goal is to evaluate the robustness of cPHS when using a fixed threshold, rather than optimizing t for each parameter setting. To this end, we focus on the balanced accuracy metric, which is commonly used to evaluate classification performance. Because our dataset exhibits approximately equal class support, we prefer this metric over the F1-score to ensure a symmetric evaluation that fully incorporates true negatives. Balanced Accuracy is defined as the average of sensitivity and specificity:

$$\mathrm{sensitivity}(t)=\mathrm{TPR}(t)\;\text{and}\;\mathrm{specificity}(t)=\mathbb{P}\left[{\hat{y}}_{t}=y|y=\text{inaccurate}\right].$$

**Figure 4:**
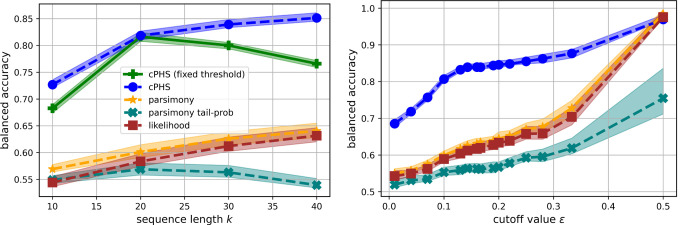
Balanced accuracy of several test statistics for the hypothesis testing in (3), with the same setting as in Figure 3. Notably, cPHS with a fixed threshold achieves a significantly higher balanced accuracy than the other approaches, even when those are applied with optimally tuned thresholds.

For each parameter configuration, we compute the balanced accuracy across several thresholds and record the maximum value. We then compare this optimal performance to that of cPHS using a fixed threshold t chosen based only on ϵ, independent of model parameters like k or m. Figure 4 summarizes this comparison. Specifically, the left panel shows results for different values of k with a cutoff value fixed at ϵ=1/7, and the right panel for different values of ϵ with k fixed at 30. In the left panel, the cPHS threshold is fixed at $t=10^{-3}$. Two conclusions can be drawn from these results: First, the performance of cPHS is similar for a fixed threshold (based solely on ϵ) and an optimally tuned one. Second, whether using fixed or optimal thresholds, cPHS consistently outperforms all other test statistics, evaluated with their own optimal thresholds. Additional results in Appendix K confirm that cPHS maintains strong performance with a fixed threshold across a wide range of values of m, n and ρ, as well as for a different value of ϵ.

### 4.3 Identification of the Ground-truth tree

**Figure 5:**
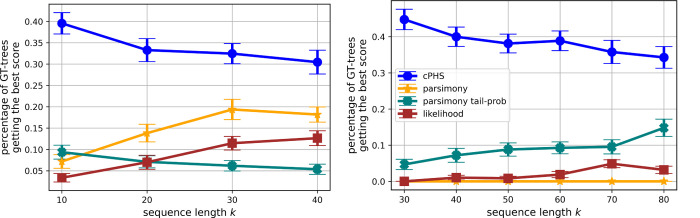
Ability to detect the ground-truth tree as a function of the sequence length k. Left: same setting as in Figure 3; in particular, n=200, k=30 and m=32. Right: larger trees, with $n=10^{3}$ cells, k=50 and m=50. Each point is calculated over 1000 realizations.

Finally, we illustrate the statistical power of cPHS over parsimony and likelihood by a different type of analysis. Specifically, we consider the following question: Suppose we are given a set of candidate trees, which includes trees reconstructed by various algorithms as well as the ground-truth tree. Is it possible to detect which tree is the ground-truth one? In other words, what is the probability that the test statistic of the ground-truth tree is better than its values for all reconstructed ones? Figure 5 illustrates results for various test statistics as a function of the sequence length k. The left panel illustrates the advantage of cPHS over parsimony and likelihood in the setting considered in this section thus far, namely with n=200 cells and m=32 possible mutations. The right panel of Figure 5 shows results for larger trees, with $n=10^{3}$ observed cells subsampled from total of 10^6^ in the original tree and m=50. Notably, in this setting the ground-truth tree is very rarely the most parsimonious or the maximum likelihood tree. In contrast, in approximately 40% of the cases, $\mathrm{cPHS}\left(T_{\text{GT}}S_{\text{GT}}\right)>\mathrm{cPHS}(TS)$ for all reconstructed trees TS. These results are in agreement with those described in previous sections.

## 5 Theoretical Support for PHS

In this section, we present theoretical support for our PHS-based approach. In Section 3.2 we proposed to assess the accuracy of a full tree by the cPHS test statistic (13). This quantity, however, is difficult to analyze theoretically, as it depends in a non-trivial way on the tail probabilities of the PHS values at all leaf pairs in the tree. Instead, we analyze a simpler PHS-based test statistic, $\text{tPHS}\left(TS\right)$, defined below. Our main result is that even this simpler measure is significantly more powerful than parsimony in detecting inaccurate tree topologies.

The PHS-based test statistic we consider in this section is defined as follows.

### Definition 4 (Total PHS).

The total PHS of a full tree TS is the sum of the PHS values over all its $\left(\begin{array}{c} n\\ 2 \end{array}\right)$ pairs of terminal leaves,

(14) $$\mathrm{tPHS}(TS)=\frac{1}{2}k\cdot \sum_{\left(u,v\right)}\mathrm{phs}(u,v),$$

where phs(u,v) is defined in Eq. (9).

To simplify the analysis, we assume that the ground-truth tree has a homogeneous (binary) tree topology.

### Definition 5 (Homogeneous tree topology).

A tree T is called homogeneous if all its edges have the same length (elapsed time). The depth d of the tree is the number of edges from the root to any of its leaves.

We consider the following scenario. Given the sequences at the terminal leaves, we assume that an algorithm reconstructed a tree topology T which is nearly identical to the ground-truth $T_{\text{GT}}$, and differs from it by a single *swap* of two leaves u, v from different sides of the original tree. Our key result is that tPHS is *qualitatively* more powerful than parsimony in detecting such incorrect topologies. We show that the difference in tPHS between the incorrect tree and the ground-truth one, normalized by the tPHS standard deviation, is much larger than the parsimony difference, normalized by its standard deviation. In simple words, the tPHS measure can provably distinguish between such correct and incorrect reconstructed trees, whereas parsimony cannot.

Next, we formally define a leaf swap. An illustration appears in Fig. S2 of Appendix F.

### Definition 6 (Leaf swap).

Let u, v be a pair of leaves in a full tree $T_{\text{GT}}S_{\text{GT}}$. Denote by ${\left(TS\right)}_{u↔v}$ the tree obtained from $T_{\text{GT}}S_{\text{GT}}$ by swapping the leaves u and v. This swap operation is followed by a correction of the sequences in the ancestors of u and v to satisfy the non-modifiability constraint: at each character i∈[k], if a mutated ancestor w of u or v has an unmutated descendant, or if two of its descendants are mutated to different states, then the ancestor is set to be unmutated, $s_{i}^{\left(w\right)}=0$. Further, denote the difference in tPHS and parsimony values following the swap by

(15) $${\text{Δ}}_{u↔v}^{P}=\mathrm{tPHS}\left({\left(TS\right)}_{u↔v}\right)-\mathrm{tPHS}\left(T_{\text{GT}}S_{\text{GT}}\right),\;\text{and}\;{\text{Δ}}_{u↔v}^{M}=M\left({\left(TS\right)}_{u↔v}\right)-M\left(T_{\text{GT}}S_{\text{GT}}\right).$$

The following theorem shows that the difference ${\text{Δ}}_{u↔v}^{P}$ is much larger than ${\text{Δ}}_{u↔v}^{M}$, both properly normalized. The proof appears in Appendix H.

### Theorem 1.

*Let* $T_{GT}S_{GT}$ *be a homogeneous full tree of depth* d *whose sequences*, *of length* k, *were generated according to the model in Section 2.1, with mutation rate* λ, *collision probability* q, *and mutation probability at a leaf* ρ *given in* (1). *Let* u *and* v *be a pair of leaves whose* LCA(u,v) *is the tree root. Let* ${\text{Δ}}_{u↔v}^{P}$ *and* ${\text{Δ}}_{u↔v}^{M}$ *be the respective change in tPHS and in parsimony*, *as defined in* (15), *following the swap* u↔v. *Then*, *for a sufficiently large* d, *their normalized expectations* $R^{P}=E\left[{\text{Δ}}_{u↔v}^{P}\right]/\sqrt{V\left[tPHS\left(T_{GT}S_{GT}\right)\right]}$ *and* $R^{M}=E\left[{\text{Δ}}_{u↔v}^{M}\right]/\sqrt{V\left[M\left(T_{GT}S_{GT}\right)\right]}$ *satisfy the following lower bound and upper bound*, *respectively*,

(16) $$R^{P}>\frac{(1-\rho q)\lambda }{4\rho \sqrt{q}}\cdot \frac{\sqrt{k}}{d},$$

(17) $$R^{M}<\frac{2(1-\rho q)\left(1-\frac{\rho }{\lambda }\right)}{(1-\rho )\lambda^{\frac{3}{2}}}\cdot \frac{\sqrt{kd^{5}}}{2^{d}}.$$

The theorem holds provided that d is sufficiently large, with the required bound depending on λ. For instance, when λ≤3, it suffices to have d≥10.

Let us discuss the consequences of this theorem. Consider a large tree with n≫1 leaves, and depth $d=\log_{2}n$. Viewing the model parameters λ, q as fixed, according to Eq. (16), for the change in tPHS to be significant it suffices that $\sqrt{k}/d≳1$. That is, the required sequence length scales only *polylogarithmically* with the number of leaves, $k≳{\left(\log_{2}n\right)}^{2}$. We note that, as proven in Erdős et al. (1999) and Wang et al. (2023), a similar relation between k and n suffices for accurate tree reconstruction under reversible and non-modifiable mutation models, respectively. In contrast, for the change in parsimony to be significant, a necessary condition is that the right-hand side of (17) is large. This translates into a significantly longer sequence of length $k≳n^{2}/{\left(\log_{2}n\right)}^{5}$, which grows *polynomially* with n. In simple words, compared to tPHS, parsimony requires exponentially longer (in tree depth) sequences to detect an incorrect topology of the form of a leaf swap.

#### *Remark* 5 (leaf swap on the same side of tree).

Theorem 1 addresses the swap of leaves whose LCA is the tree root. The theorem can be easily generalized to the swap of leaves u, v whose LCA(u,v) is an inner node. This would result in two changes to the RHS of Equations (16) and (17). First, instead of d we would have d−l, where l is the depth of LCA(u,v). Second, instead of k we would have $k\cdot e^{-\lambda l/d}$, the expected number of unmutated characters at the node LCA(u,v). As long as l is much smaller than d, namely LCA(u,v) is close enough to the root, the consequences of the theorem continue to hold. Specifically, tPHS would be able to detect such a leaf swap with a sequence length polylogarithmic in n, whereas parsimony would require a sequence length polynomial in n.

#### *Remark* 6 (several leaf swaps). Theorem 1 considers a single leaf swap.

The theorem can be readily extended to the case of several leaf swaps, provided that the different LCAs of the leaf pairs are located at distinct branches of the tree. In particular, the LCAs must be inner nodes; see Remark 5. In this case, the effect of the leaf swaps on both the tPHS and parsimony is additive.

## 6 cPHS evaluation on a dataset of lineage tracing in lung adenocarcinoma

To evaluate the cPHS score with real-world data, we applied it to a dataset of lineage tracing in a mouse model of lung adenocarcinoma from Yang et al. (2022). This dataset features an inducible lineage recording system consisting of Cas9, synthetic DNA elements that can be targeted by Cas9 (acting as lineage recording sites), and guide RNAs that help direct Cas9 to those sites. The lineage recording system was integrated into the genome of mice that already harbored inducible oncogenic mutations of the genes Kras and Trp53. By direct administration of Cre-carrying virus to the lungs of the engineered animals (referred to as KPTracers), the authors have induced two processes that operate on the lung epithelium in parallel: malignant transformation (switching on oncogenic mutations in Kras and Trp53) and lineage recording (switching on Cas9). The resulting tumors, including both primary and metastatic lesions, were obtained between five to six months after induction and analyzed using single cell RNA-sequencing. This analysis provided both lineage traces (i.e., the sequence of each target site - either mutated or untouched) and transcriptome information (i.e., estimation of expression for thousands of genes) for each tumor cell.

Our dataset consisted of 63 samples (tumors), spanning a wide range of parameters, including the number of cells per tumor (106≤n≤4798, average = 689), collision probabilities (0.06≤q≤0.77, average = 0.28), mutation rates (1.2≤λ≤7.3, average = 3.1), and number of successfully captured recording sites (6≤k≤67, average = 25). See Supplementary Table (KPTracer_summary.xlsx) for the complete description of these samples and their analysis. To evaluate cPHS we first generated a set of negative controls for each tumor, consisting of five random binary trees built over the same set of leaves (i.e., tumor cells and their lineage traces). To compute cPHS scores of these trees we followed the rationale presented in Section 4 and used dedicated simulation analysis to select threshold values t based on the observed parameters (see Appendix M). We find that the ability to reject random trees tracks closely with the quality of the respective sample (Figures S17, S18). Specifically, randomized trees built for the 42 samples that had a sufficient number of recording sites (k>15) and did not exhibit high collision probabilities (q<0.35) were all rejected (0 of 210 random trees accepted as accurate by cPHS). These correspond to good quality samples with sufficient rates of integration of lineage recording sites and to a sufficient capture rate of RNA molecules (resulting in sufficiently large k). They also correspond to cases with well-calibrated affinity of the guide RNAs, avoiding early saturation in which many cells in the eventual tumor have identical lineage traces (thus high q). These results are further supported by a more extensive simulation analysis, finding that also in fully simulated data where the ground truth is known, in the regime of insufficient-quality (k≤15 or q≥0.35) no value of t reliably separates accurate from inaccurate reconstructions for any reasonable choice of ϵ (Tables S1, S2, S3, S4).

**Figure 6:**
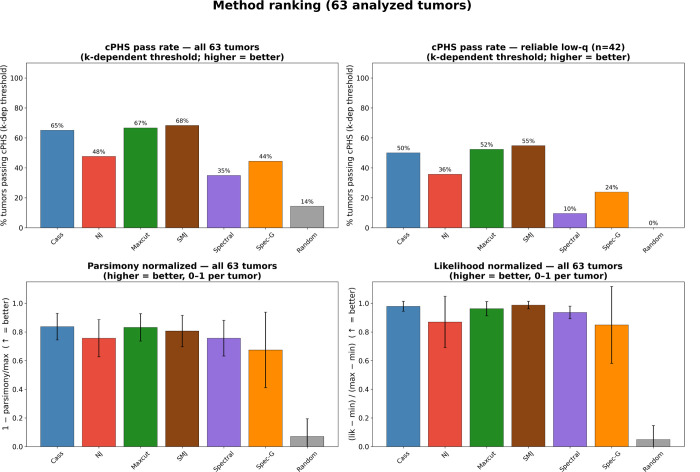
Method ranking. Top row: cPHS pass rate across all 63 tumors (left) and across the 42 reliable low-q tumors (right), both using the calibrated k-dependent thresholds ($t=10^{-3}$ for k≤24, $t=10^{-4}$ for k≥25). Bottom row: normalized parsimony (left) and normalized likelihood (right) across all 63 tumors, where higher values indicate better performance. Error bars show standard deviations across tumors.

We next reconstructed lineage trees for each tumor sample using six algorithms that are implemented in the Cassiopeia package (Jones et al., 2020), including Neighbor Joining, Shared Mutation Joining (SMJ), Max-Cut, a compatibility-based heuristic (Cassiopeia greedy), and two algorithms based on spectral decomposition. Of the 42 tumors with sufficient quality, cPHS confirmed at least one of the six reconstructions as valid in 28 of the cases. Importantly, all the 14 samples for which the trees were rejected fall within a regime of low k to n ratio (k/n<0.05). All other samples had a higher ratio and had at least one inferred tree (out of the six inferred per sample) that was accepted by cPHS. This result mirrors the theoretically-established relationship between n and the minimal value of k that is required to achieve accurate reconstruction (Wang et al., 2023). Among the algorithms used for inference, SMJ, Max-Cut, and compatibility-based greedy received the highest acceptance rate by cPHS (50%−55% of trees; see Figure 6). The two spectral methods had the lowest acceptance rate, and consistently had the lowest ranks in terms of parsimony and likelihood (Figure 6).

We next aimed to verify that cPHS reflects topological errors rather than merely separating reconstructions from random trees. Focusing on two of the inference algorithms (SMJ and Cassiopeia greedy), we degraded the reconstructed trees by a controlled number of Nearest Neighbor Interchange (NNI) moves, which swap subtrees across internal edges and progressively scramble the topology while leaving the character data unchanged. As the fraction of NNI moves increases, the topology drifts further from the original reconstruction. The various metrics reflect this worsening: parsimony and Robinson-Foulds distance rise, while likelihood and triplets decrease (Figure S21). The cPHS acceptance rate acts accordingly with the level of perturbation, with a monotonic decline as the NNI rate increases (Figure S20; Appendix P).

Together, these results demonstrate the applicability of cPHS to real-world data and particularly the merit of enabling an accept/reject decision, beyond ranking. First, it flags parameter regimes in which the inferred trees are a-priori less likely to be accurate, corresponding to tumor samples with insufficient quality or to large samples with too few recording sites. Second, it is able not only to distinguish between random and inferred trees by its raw score, but to also reject all random trees using its classification step. Finally, it is able to sense increasingly-dominant perturbations to the inferred trees, with a reduction in the acceptance rates.

## 7 Discussion

As demonstrated via simulations in Section 4 and supported by theoretical analysis in Section 5, our method offers significantly greater discriminative power than traditional parsimony. Intuitively, this advantage may be explained as follows: parsimony aggregates a local measure — the number of mutations per edge — without accounting for the broader tree structure. In contrast, PHS integrates information across all pairs of leaves, both close and distant, thereby capturing the consistency of the tree’s topology as a whole.

In our work, we considered the minimal adjusted p-value as our test statistic (13). The key parameter of our procedure is the threshold t against which the cPHS statistic is compared. As illustrated in Section 6, in practical settings, a suitable threshold t can be estimated via simulations designed to reflect the characteristics of the experimental setting. Specifically, one may generate multiple ground-truth trees, for example via Cassiopeia (Jones et al., 2020). Given the observed sequences for each ground-truth tree, various reconstruction algorithms can be run, followed by computing the cPHS scores for their outputs. A suitable threshold can then be determined based on the cPHS scores obtained for the most accurate reconstructions. We remark that there are other possible schemes to fuse the scores in (11) into a single test statistic, e.g. the harmonic mean (Wilson, 2019). Our simulations indicate that the statistical power of the harmonic mean is comparable to that of cPHS. However, it is less practical, as in contrast to cPHS, the harmonic mean is very sensitive to the specific value of the threshold t.

In this manuscript, we focused on a non-modifiable model of evolution, whereby Lemma 1, and consequently our cPHS test statistic (13), are based on this assumption. Our proposed homoplasy approach may also be beneficial for assessing the accuracy of reconstructed trees under reversible models. This requires deriving an analogue of Lemma 1 for a given reversible model of evolution. We leave this extension for future work.

Another interesting direction for future research is to use a homoplasy approach not merely to assess the accuracy of trees, but in fact to develop a PHS-based tree reconstruction algorithm. As we illustrated both empirically and theoretically, PHS provides a better tree accuracy measure than parsimony. Hence, it would be interesting to explore if a PHS-based algorithm can reconstruct more accurate trees than those found by current methods that aim to find the tree with maximum parsimony for the observed data.

Finally, the core principles of the PHS framework can be leveraged to evaluate branch support. Currently, cPHS(TS) acts as a global test statistic by aggregating the tail probabilities of all $N=\left(\begin{array}{c} n\\ 2 \end{array}\right)$ leaf pairs in the tree. However, to assess the confidence of a specific internal branch, one could restrict this evaluation to the subset of leaf pairs whose latest common ancestor corresponds to the node defining that branch. By aggregating the adjusted PHS p-values strictly within a specific clade, our approach could theoretically provide a statistical measure of branch support tailored to the irreversible constraints of CRISPR-Cas9 lineage tracing. Because this approach relies on analytical probabilities rather than sequence resampling, it could potentially offer an advantage over traditional bootstrapping, which is often sensitive to the limited sequence lengths typical of CRISPR-Cas9 target arrays.

## Supplementary Material

Supplement 1

Supplement 2

## Figures and Tables

**Table 1:** Model parameters.

number of leaves	n
sequence length	k
number of mutated states	m
mutation probability to state j collision probability mutation rate	$q_{j}$
collision probability	$q\equiv \sum_{j=1}^{m}q_{j}^{2}$
mutation rate	λ
character mutation probability at a leaf	$\rho \equiv 1-e^{-\lambda }$

## Footnotes

1 A Python implementation of our method will be made publicly available upon publication.
