# Supplementary material for: Can you trust your reconstructed lineage tree? A homoplasy-based approach for irreversible evolution
Source: bioRxiv. 2026 Jul 5:2025.07.27.667007. Preprint. [Version 2] doi: 10.1101/2025.07.27.667007 (PMC13344993; doi:10.1101/2025.07.27.667007)
Supplement: Supplement 2 [file NIHPP2025.07.27.667007v2-supplement-2.pdf]

bioRxiv preprint doi: https://doi.org/10.1101/2025.07.27.667007; this version posted July 5, 2026. The copyright holder for this preprint (which was not certified by peer review) is the author/funder, who has granted bioRxiv a license to display the preprint in perpetuity. It is made available under a CC-BY 4.0 International license.

Supplementary Material for:

Can you trust your reconstructed lineage tree? A homoplasy-based approach for irreversible evolution

Pini Zilber, Sebastian Prillo, Yaara Neumeier, Nir Yosef, and Boaz Nadler

A Handling Model Uncertainty and Incomplete Reconstructions

In this section, we address some practical aspects in applying the cPHS statistic. Specifically, we discuss how to estimate unknown model parameters required for the computation; how to handle cases where only the reconstructed tree topology is available without internal node sequences; and how to prevent saturation of the cPHS score in reconstructed trees with very few homoplasies.

A.1 Unknown model parameters and birth times

As discussed in the main text, our PHS test statistic depends on two model parameters: the mutation rate λ and the collision probability q. In addition, in terms of the given tree, it depends on the birth times of the inner nodes. In this section, we discuss their estimation.

Let $\hat{\rho}$ be the fraction of mutated characters in the sequences at the observed cells. Then, as in Jones et al. (2020), the mutation rate λ can be accurately estimated from $\hat{\rho}$ via the plug-in estimate $\hat{\lambda} = -\log(1-\hat{\rho})$; see (1). The collision probability q, in contrast, cannot typically be accurately estimated given the result of a single or few clones, since we do not know when up in the tree an observed mutation occurred. However, this parameter is constant for a fixed experimental system (Jones et al., 2020; Seidel and Stadler, 2022). It is thus possible to estimate it from single-cell RNA-seq data of many clones or even from bulk RNA-sequencing of the target sites, as done in Jones et al. (2020) and Seidel and Stadler (2022). In our simulations, we assume knowledge of q, and estimate λ from the observations. Finally, the birth times τ of the inner nodes are estimated using the maximum-likelihood-based branch length estimator proposed in Prillo et al. (2026). In particular, this estimator lower-bounds the minimal branch length for a mutationless edge.

A.2 Reconstructed topology $\mathcal{T}$ without internal node sequences $\mathcal{S}$

In the main text, it was assumed that the output of a given algorithm is a reconstructed full tree $\mathcal{TS}$, which includes both the tree topology $\mathcal{T}$ and the internal node sequences $\mathcal{S}$. However, some algorithms output only a reconstructed tree topology $\mathcal{T}$ without a corresponding $\mathcal{S}$. Given $\mathcal{T}$ and $\mathcal{S}_{\text{obs}}$, there is more than one possible set of internal node sequences $\mathcal{S}$ consistent with the non-modifiability model assumption. Since to compute the cPHS score requires also the set of internal sequences, the cPHS score "induced" by only a reconstructed topology is not uniquely defined. Similarly, the hypothesis testing (3) with either the parsimony or the likelihood distance is also not well-defined. In this section, we propose a procedure to reject candidate topologies w.r.t. *the parsimony distance*, even without internal sequences.

We first present the procedure, and then its justification. Assuming we are given only a candidate topology $\mathcal{T}$ without reconstructed internal sequences, we first compute the solution to the small MP problem $\mathcal{S}_{\text{MP}}$, under the non-modifiability constraint. This is described in Appendix C and Alg. 2. Next, we compute the test statistic $\text{cPHS}(\mathcal{TS}_{\text{MP}})$. If our test rejects $\mathcal{TS}_{\text{MP}}$, then we reject the topology $\mathcal{T}$ itself.

The justification of this procedure is based on the following lemma, whose proof appears in Appendix E. It states that the solution to the small MP problem maximizes the cPHS score among all the possible sets of sequences that comply with the non-modifiability constraint and coincide with the observed sequences at the leaves.

Lemma 2. *Let $\mathcal{T}$ be a fixed tree topology, and let $\mathcal{S}_n$ be a given set of sequences at its leaves with no missing data. Denote by $\mathcal{S}_{MP}$ a solution of the small MP problem. Then for any set of sequences $\mathcal{S}$ at all tree nodes that complies with the non-modifiability constraint and whose leaves coincide with $\mathcal{S}_n$, it holds that*

$$cPHS(\mathcal{TS}_{MP}) \geq cPHS(\mathcal{TS})\,. \tag{S1}$$

bioRxiv preprint doi: https://doi.org/10.1101/2025.07.27.667007; this version posted July 5, 2026. The copyright holder for this preprint (which was not certified by peer review) is the author/funder, who has granted bioRxiv a license to display the preprint in perpetuity. It is made available under a CC-BY 4.0 International license.

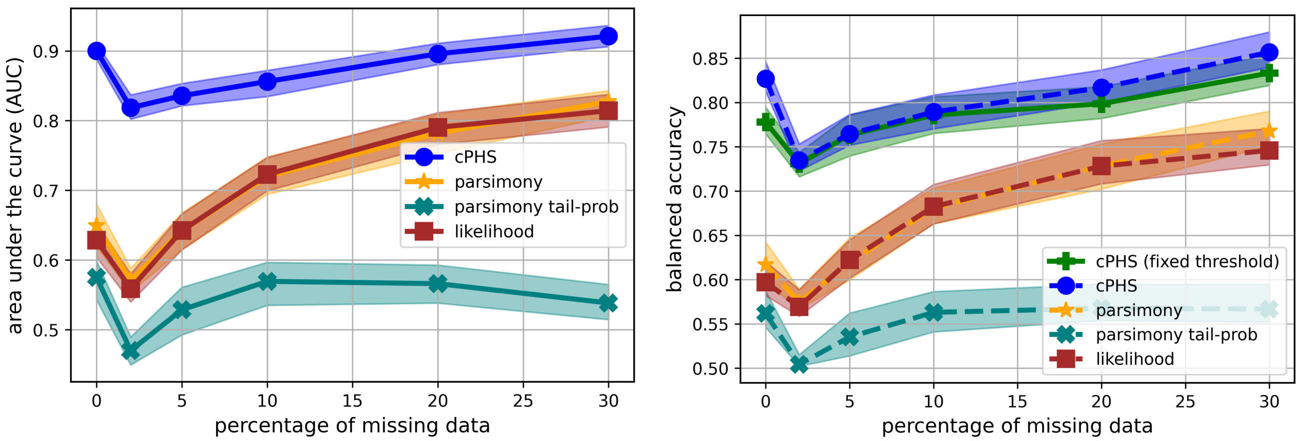

Figure S1: AUC values (left) and balanced accuracy (right) of several test statistics for the hypothesis testing in (3), with the same setting as in Figures 3 and 4 at $k = 30$ and $m = 32$. The x-axis is the total proportion of missing data p_{miss}.

Suppose that our procedure rejected $\mathcal{TS}_{\text{MP}}$. Assuming our test result is correct, $\mathcal{TS}_{\text{MP}}$ satisfies hypothesis $\mathcal{H}_1$ of (3), namely $\mathcal{TS}_{\text{MP}} > (1+\epsilon) \cdot M(\mathcal{T}_{\text{GT}}\mathcal{S}_{\text{GT}})$. Hence, by definition of the small MP problem, $\mathcal{TS}_{\text{MP}}$ satisfies $\mathcal{H}_1$ of (3) for any $\mathcal{S}$. The topology $\mathcal{T}$ should thus be rejected, as required.

A.3 Very few homoplasies

In an extreme case where the reconstructed full tree has very few homoplasies, it may occur that all sorted scores satisfy

$$s_i \geq i/N, \quad \forall i, \tag{S2}$$

In this case, using (13) leads to a saturation in cPHS, outputting the maximal possible value of 1. This precludes a finer comparison of different trees that both obtain cPHS = 1. To overcome this issue, we exclude leaf pairs with no homoplasies, $\{i : s_i = 1\}$, before taking the minimum. Formally, Eq. (13) is modified to

$$\text{cPHS}(\mathcal{TS}) = \min_{i:\, s_i < 1} s'_i.$$

B Missing Data

In the main text, we assumed a complete (i.e., fully observed) data matrix $\mathcal{S}_n$. In practice, however, some entries in $\mathcal{S}_n$, corresponding to characters at the tree leaves, may be unknown. The CRISPR-Cas9 evolution model has two distinct mechanisms of missing data. The first is stochastic missingness, which arises from imperfections in the sequencing process, leading to randomly missing entries in $\mathcal{S}_n$. The second is heritable missingness, which occurs when characters at internal nodes are absent due to transcriptional silencing or double Cas9 resection. These missing characters behave analogously to mutations: their unknown state is non-modifiable and is inherited by all descendant nodes, including the leaves. Let p_{sto} and p_{her} denote the rates of stochastic and heritable missingness in $\mathcal{S}_n$, respectively. Assuming independence, the overall missing data rate is then given by $p_{\text{miss}} = p_{\text{sto}} + p_{\text{her}} - p_{\text{sto}} \cdot p_{\text{her}}$.

To deal with the presence of missing data, two minor modifications are required in the definition of the cPHS measure. Let u and v be a pair of leaf nodes. First, we revise the definition of $\text{phs}_i(u,v)$ in Eq. (8) as follows: if the i-th character of either u or v is missing, then $\text{phs}_i(u,v) = 0$. Second, we modify Eq. (11) in Step 1 of the cPHS computation procedure by replacing k with $k_{\text{eff}}(u,v)$, defined as the number of characters that are non-missing in both u and v.

As illustrated in Figure S1, the adapted cPHS performs well in the presence of missing data, and in particular, it retains its advantage over the other test statistics. Interestingly, the performance of cPHS, as well as that of the other test statistics, improves as p_{miss} increases. This trend, however, may be attributed to a technical factor: the balance between accurately and inaccurately reconstructed trees shifts with increasing p_{miss}. Specifically, as

bioRxiv preprint doi: https://doi.org/10.1101/2025.07.27.667007; this version posted July 5, 2026. The copyright holder for this preprint (which was not certified by peer review) is the author/funder, who has granted bioRxiv a license to display the preprint in perpetuity. It is made available under a CC-BY 4.0 International license.

p_{miss} increases, the reconstruction task becomes more challenging, leading to a higher proportion of inaccurate trees produced by the algorithms. It is reasonable to assume that this shift is the primary driver of the observed improvement in the test statistics' performance.

C Small and Large Parsimony Problems

Let us recall the definitions of the small and large maximum parsimony (MP) problems Felsenstein (2004, Chapters 2 and 4).

Definition 7 (Small MP)**.** In the small MP problem, given a tree topology $\mathcal{T}$ and observed sequences at the leaves $\mathcal{S}_n$, the goal is to reconstruct the sequences at the ancestral (internal) nodes such that the resulting tree has the smallest number of mutations:

$$\min_{\mathcal{S}} \{M(\mathcal{TS}) : \text{leaves}(\mathcal{S}) = \mathcal{S}_n\}. \tag{S3}$$

Whereas small MP optimizes only over the internal sequences for a fixed topology, large MP additionally optimizes over the tree topologies:

Definition 8 (Large MP)**.** In the large MP problem, given the observed sequences at the leaves $\mathcal{S}_n$, the goal is to reconstruct a full tree with the smallest number of mutations:

$$\min_{\mathcal{TS}} \{M(\mathcal{TS}) : \text{leaves}(\mathcal{S}) = \mathcal{S}_n\}. \tag{S4}$$

The small MP problem can be solved efficiently by the Sankoff algorithm (Sankoff, 1975). The non-modifiability constraint can be forced by setting the substitution matrix accordingly. The resulting algorithm, presented in Alg. 2, is simple, and bears a resemblance to Fitch algorithm (Fitch, 1977). In the absence of missing data, the solution to the small MP problem under the non-modifiability constraint is unique (Sashittal et al., 2023). The large MP problem, in contrast, is NP-hard in general (Felsenstein, 2004), and remains so under the non-modifiable model (Day, Johnson, and Sankoff, 1986; Sashittal et al., 2023).

D Parsimony Tail Probability

In this section, we derive the parsimony distribution conditional on a given candidate tree topology, where the inner sequences are random. The distribution is calculated recursively along the tree topology Let w be an unmutated node in the tree, and denote the number of mutations at the i-th character in the subtree whose root is w by $M_w^{(i)}$. If w is a leaf, then no mutation can occur from w downwards, namely $\mathbb{P}[M_w^{(i)} = j] = \delta_{j,0}$. Next, consider the case where w is an inner node. Denote its children by u and v, and the edge length from w to u and to v by $\tau_{w,u}$ and $\tau_{w,v}$, respectively. Recall that the edge length is the time elapsed between the birth time of a node and its child.

Let U and V be the events of mutation occurrence at the i-th character from w to u and to v, respectively. Then $\mathbb{P}[U] = 1 - e^{-\lambda\tau_{w,u}}$ and $\mathbb{P}[V] = 1 - e^{-\lambda\tau_{w,v}}$. In addition, by the Markovian property of the generative process, U and V are independent given that w is unmutated. By the law of total probability,

$$\begin{aligned}
\mathbb{P}\left[M_w^{(i)} = j\right] &= \mathbb{P}[U^c \cap V^c] \cdot \mathbb{P}\left[M_w^{(i)} = j \mid U^c \cap V^c\right] + \mathbb{P}[U^c \cap V] \cdot \mathbb{P}\left[M_w^{(i)} = j \mid U^c \cap V\right] \\
&\quad + \mathbb{P}[U \cap V^c] \cdot \mathbb{P}\left[M_w^{(i)} = j \mid U \cap V^c\right] + \mathbb{P}[U \cap V] \cdot \mathbb{P}\left[M_w^{(i)} = j \mid U \cap V\right] \\
&= e^{-\lambda(\tau_{w,u}+\tau_{w,v})} \cdot \mathbb{P}\left[M_w^{(i)} = j \mid U^c \cap V^c\right] + e^{-\lambda\tau_{w,u}}\left(1 - e^{-\lambda\tau_{w,v}}\right) \cdot \mathbb{P}\left[M_w^{(i)} = j \mid U^c \cap V\right] \\
&\quad + \left(1 - e^{-\lambda\tau_{w,u}}\right) e^{-\lambda\tau_{w,v}} \cdot \mathbb{P}\left[M_w^{(i)} = j \mid U \cap V^c\right] + \left(1 - e^{-\lambda\tau_{w,u}}\right)\left(1 - e^{-\lambda\tau_{w,v}}\right) \cdot \mathbb{P}\left[M_w^{(i)} = j \mid U \cap V\right].
\end{aligned}$$

If both u and v are unmutated (namely under $U^c \cap V^c$), $M_w^{(i)} = M_u^{(i)} + M_v^{(i)}$. As $M_u^{(i)}$ and $M_v^{(i)}$ are independent, $\mathbb{P}\left[M_w^{(i)} = j \mid U^c \cap V^c\right] = \sum_{i=0}^{j} \mathbb{P}\left[M_u^{(i)} = i\right] \cdot \mathbb{P}\left[M_v^{(i)} = j - i\right]$. If v is mutated but not u ($U^c \cap V$), $M_w^{(i)} = M_v^{(i)} + 1$ due to the non-modifiability of the evolution process. Hence, $\mathbb{P}\left[M_w^{(i)} = j \mid U^c \cap V\right] = \mathbb{P}\left[M_u^{(i)} = j - 1\right]$. Similarly, $\mathbb{P}\left[M_w^{(i)} = j \mid U \cap V^c\right] = \mathbb{P}\left[M_v^{(i)} = j - 1\right]$. Finally, if both u and v are mutated, $M_w^{(i)} = 2$ due to non-modifiability,

bioRxiv preprint doi: https://doi.org/10.1101/2025.07.27.667007; this version posted July 5, 2026. The copyright holder for this preprint (which was not certified by peer review) is the author/funder, who has granted bioRxiv a license to display the preprint in perpetuity. It is made available under a CC-BY 4.0 International license.

Algorithm 2: Solution to the small MP problem under non-modifiable model

input : Tree topology $\mathcal{T}$ and sequences at leaves $\mathcal{S}^{(\text{leaves})}$
output: Full tree $\mathcal{TS}$

Function *smallMP($\mathcal{TS}$)*
  /* reconstruct ancestral states at internal nodes by solving the small MP problem */
  $\mathcal{TS} \leftarrow \text{smallMP}(\mathcal{TS}, \text{root})$
  /* set the sequence at the root to zeros */
  for $i = 1$ *to* k **do**
    $S_i^{(\text{root})} \leftarrow 0$
  end
  return $\mathcal{TS}$
end

Function *smallMP($\mathcal{TS}$, node)*
  if *node is not a leaf* **then**
    /* solve recursively for each of the two node's children */
    $s^{(\text{left})} \leftarrow \text{smallMP}(\mathcal{TS}, \text{leftChild(node)})$
    $s^{(\text{right})} \leftarrow \text{smallMP}(\mathcal{TS}, \text{rightChild(node)})$
    /* determine node's sequence according to its children, taking non-modifiability into account */
    for $i = 1$ *to* k **do**
      if $s_i^{(left)} = 0$ *or* $s_i^{(right)} = 0$ *or* $s_i^{(left)} \neq s_i^{(right)}$ **then**
        $S_i^{(\text{node})} \leftarrow 0$
      else
        $S_i^{(\text{node})} \leftarrow s_i^{(\text{left})}$
      end
    end
  return $S^{(\text{node})}$
end

and thus $\mathbb{P}\left[M_w^{(i)} = j \mid U \cap V\right] = \delta_{j,2}$. Putting everything together, we conclude

$$\begin{aligned}\mathbb{P}\left[M_w^{(i)} = j\right] &= e^{-\lambda(\tau_{w,u}+\tau_{w,v})} \cdot \sum_{i=0}^{j} \mathbb{P}\left[M_u^{(i)} = i\right] \mathbb{P}\left[M_v^{(i)} = j - i\right] + e^{-\lambda\tau_{w,u}} \left(1 - e^{-\lambda\tau_{w,v}}\right) \cdot \mathbb{P}\left[M_u^{(i)} = j - 1\right] \\ &\quad + \left(1 - e^{-\lambda\tau_{w,u}}\right) e^{-\lambda\tau_{w,v}} \cdot \mathbb{P}\left[M_v^{(i)} = j - 1\right] + \left(1 - e^{-\lambda\tau_{w,u}}\right)\left(1 - e^{-\lambda\tau_{w,v}}\right) \cdot \delta_{j,2}.\end{aligned}$$

This recursive equation allows us to compute the parsimony distribution at a node w at the i-th character, $\mathbb{P}[M_w^{(i)}]$, as a function of its children distributions, $\mathbb{P}[M_u^{(i)}]$ and $\mathbb{P}[M_v^{(i)}]$. The distribution of the total parsimony (sum of mutations at all characters) of a tree topology $\mathcal{T}$ with random sequences $\mathcal{S}$ is given by convolving the individual distributions $\mathbb{P}[M_{\text{root}}^{(i)}]$,

$$\mathbb{P}[M(\mathcal{TS}) = M_{\text{root}}] = \mathbb{P}\left[M_{\text{root}}^{(1)}\right] \circledast \ldots \circledast \mathbb{P}\left[M_{\text{root}}^{(k)}\right] = \mathbb{P}\left[M_{\text{root}}^{(1)}\right] \circledast \ldots \circledast \mathbb{P}\left[M_{\text{root}}^{(1)}\right].$$

The second equality follows from the assumption of uniform mutation rate; see Remark 4. Finally, the parsimony tail probability for a given tree topology $\mathcal{T}$ is $\mathbb{P}[M(\mathcal{TS}) \leq M_{\text{root}}]$.

bioRxiv preprint doi: https://doi.org/10.1101/2025.07.27.667007; this version posted July 5, 2026. The copyright holder for this preprint (which was not certified by peer review) is the author/funder, who has granted bioRxiv a license to display the preprint in perpetuity. It is made available under a CC-BY 4.0 International license.

E Proofs of Lemmas 1 and 2

Proof of Lemma 1. Let $p_{u,v} = \mathbb{P}[\text{phs}_1(u,v) = 1]$, where the probability is over the random sequences $\mathcal{S}_{\text{GT}}$. Then, by the definition of homoplasy, with $w = \text{LCA}(u,v)$,

$$\begin{aligned} p_{u,v} &= \mathbb{P}[s_1^{(w)} = 0 \wedge s_1^{(u)} \neq 0 \wedge s_1^{(u)} = s_1^{(v)}] \\ &= \mathbb{P}[s_1^{(w)} = 0] \cdot \mathbb{P}[s_1^{(u)} \neq 0, s_1^{(v)} \neq 0 \mid s_1^{(w)} = 0] \cdot \mathbb{P}[s_1^{(u)} = s_1^{(v)} \mid s_1^{(w)} = 0 \wedge s_1^{(u)} \neq 0 \wedge s_1^{(v)} \neq 0]. \end{aligned} \tag{S5}$$

Let us analyze each of the three terms in the RHS of (S5). The first expression is simply the probability that the first character in $s^{(w)}$ is unmutated, which is given by

$$\mathbb{P}[s_1^{(w)} = 0] = \alpha_{\tau_w}.$$

Regarding the second term, since characters at node u and at node v evolve independently, conditional on their LCA w being unmutated,

$$\mathbb{P}[s_1^{(u)} \neq 0, s_1^{(v)} \neq 0 \mid s_1^{(w)} = 0] = \mathbb{P}[s_1^{(u)} \neq 0 \mid s_1^{(w)} = 0] \cdot \mathbb{P}[s_1^{(v)} \neq 0 \mid s_1^{(w)} = 0].$$

The probability that the character at u is unmutated given that w is unmutated is $e^{-\lambda\tau_{w,u}}$ where $\tau_{w,u} = \tau_u - \tau_w = 1 - \tau_w$, where the second equality follows from the fact that u is a leaf. Hence, $\mathbb{P}[s_1^{(v)} \neq 0 \mid s_1^{(w)} = 0] = \beta_{\tau_w}$. As a similar argument holds for v,

$$\mathbb{P}[s_1^{(u)} \neq 0, s_1^{(v)} \neq 0 \mid s_1^{(w)} = 0] = \beta_{\tau_w}^2.$$

As for the third term, since a mutated character is j with probability q_j, the probability of a collision (same character in both nodes) is given by

$$\mathbb{P}[s_1^{(u)} = s_1^{(v)} \mid s_1^{(w)} = 0 \wedge s_1^{(u)}, s_1^{(v)} \neq 0] = \sum_{j=1}^{m} q_j^2 = q.$$

Inserting all of these into (S5) yields $p_{u,v} = \alpha_{\tau_w}\beta_{\tau_w}^2 q$. The Binomial distribution in (10) of the lemma follows from the fact that characters at different locations evolve independently with the same parameters λ and q. ☐

Proof of Lemma 2. Let $\mathcal{S}$ be a set of sequences that coincides with $\mathcal{S}_{\text{GT}}$ at the leaves. Assume that for any pair of leaves u, v,

$$\text{phs}\left(u, v \mid \mathcal{S}_{\text{MP}}\right) \leq \text{phs}\left(u, v \mid \mathcal{S}\right). \tag{S6}$$

Then, by (11), $s(u, v \mid \mathcal{S}_{\text{MP}}) \geq s(u, v \mid \mathcal{S})$. Therefore, upon computing (10), (11), sorting the s-values, adjusting them by (12) and taking the minimum as in (13), (S1) of the lemma follows. Hence, we now prove that (S6) indeed holds.

Let u, v be a pair of leaves, and let $w = \text{LCA}(u, v)$. For simplicity, we focus on the first character location. Since characters evolve identically and independently, the same argument holds for all the characters.

Let z_1, z_2 be the immediate children of w. There are four generic possible scenarios for $\left(s_1^{(z_1)}, s_1^{(z_2)}\right)$: $(0,0), (0,1), (1,2)$ and $(1,1)$. All the other possibilities are equivalent to one of these four. Under the non-modifiable model, $s_1^{(w)}$ must be 0 in the first three cases. The only freedom of choice left to the inner sequences reconstruction algorithm is in the case of $s_1^{(z_1)} = s_1^{(z_2)} = 1$, which we now analyze in detail. In this case, all the descendants of w have 1 in their first character. Hence, assigning $s_1^{(w)} = 1$ minimizes the PHS of all leaf pairs that are descendants of w, including u and v. It is left to show that in $\mathcal{S}_{\text{MP}}$, w is indeed assigned with 1 in this case.

Let us analyze the effect of $s_1^{(w)}$ on the total number of mutations, $M(\mathcal{T}\mathcal{S}_{\text{MP}})$. Assigning $s_1^{(w)} = 1$ increases the total number of mutations by either 0 (if the sibling of w has 1) or 1 (if it has 0). Assigning w with 0, on the other hand, increases the total number of mutations by at least 2, as two changes are required from $s_1^{(w)}$ to $s_1^{(z_1)}$ and to $s_1^{(z_2)}$. Hence, the small MP solution assigns $s_1^{(w)}$ with 1, as required. ☐

In the main text, we assume a uniform mutation rate λ across all k characters. However, as claimed in Remark 4, our results can be easily generalized. This is formalized in the following claim.

bioRxiv preprint doi: https://doi.org/10.1101/2025.07.27.667007; this version posted July 5, 2026. The copyright holder for this preprint (which was not certified by peer review) is the author/funder, who has granted bioRxiv a license to display the preprint in perpetuity. It is made available under a CC-BY 4.0 International license.

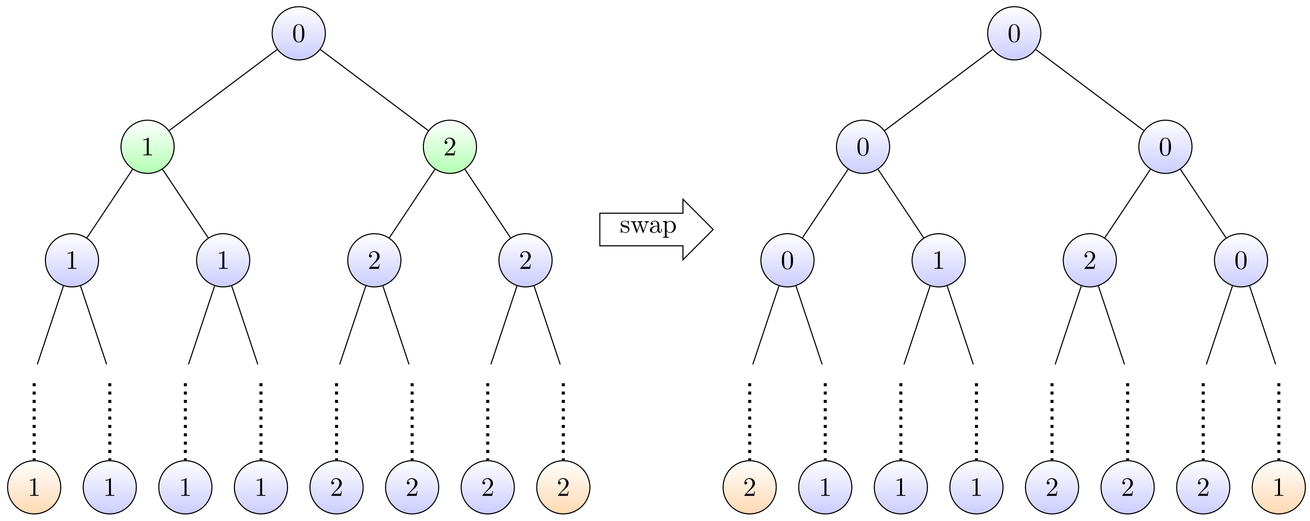

Figure S2: Effect of a leaf swap. Left: a ground-truth tree where mutations occurred early in the tree (at the green nodes). As a result, each of its two subtrees has a different mutated state. Right: a reconstructed tree which differs from the GT tree by a single swap (the swapped leaves are marked in orange). The inner node sequences are reconstructed to satisfy the non-modifiability assumption.

Claim 1 (non-uniform mutation rates)**.** *Under the assumptions of Lemma 1 but with non-uniform mutation rates λ_i for each $i \in [k]$, the probability distribution of $k \cdot phs(u, v)$ is Poisson Binomial with*

$$p_i = \alpha^i_{\tau_w} \left(\beta^i_{\tau_w}\right)^2 q, \tag{S7}$$

where $\alpha^{(i)}_{\tau_w} = e^{-\lambda_i \tau_w}$ and $\beta^{(i)}_{\tau_w} = 1 - e^{-\lambda_i(1-\tau_w)}$.

Proof. By following the steps of the proof of Lemma 1, we get that $p_{u,v} = \alpha^{(1)}_{\tau_w} \left(\beta^{(1)}_{\tau_w}\right)^2 q = p_1$. More generally,

$$\mathbb{P}[\text{phs}_1(u, v) = i] = p_i.$$

The claim follows from the independence of different characters and the definition of the Poisson Binomial distribution. □

F Illustration of a Leaf Swap, and its Effects on PHS and Parsimony

Let us illustrate the difference in tPHS and parsimony values following a leaf swap. For simplicity, the sequence length in our example consists of a single character, $k = 1$. Suppose that in the ground-truth tree, the immediate offspring of the root mutated to two different states: the left branch mutated to 1 and the right branch to 2. As a result, the state of all the leaves in the left subtree is 1, and it is 2 in the right subtree; see Figure S2(left) for an illustration. Now, suppose that a tree reconstruction algorithm made a single error of swapping the leftmost and rightmost leaves; see Figure S2(right). In this example, there are no homoplasies in the ground-truth tree, and all its leaf pairs have $\text{phs}(u, v) = 0$. In the reconstructed tree, in contrast, a non-negligible fraction of leaf pairs have a PHS of 1. As stated in Lemma 10 below, in this case the increase in tPHS scales as the number of leaf pairs, which is roughly 4^d. This is comparable to the tPHS standard deviation over random inner sequences; see Lemma 4. Hence, PHS is sufficiently powerful to identify the reconstructed tree as incorrect. In contrast, as presented in Lemma 5, the parsimony increase is only linear in d. This is negligible compared to the parsimony standard deviation over random inner sequences, which scales as the number of leaves, $n = 2^d$; see Lemma 3. As a result, the parsimony measure is unable to distinguish between the correct and the incorrect tree.

G Auxiliary Lemmas for Theorem 1

The proof of Theorem 1 makes use of four auxiliary lemmas, outlined below. In the following, $\mathcal{L}(\mathcal{T})$ denotes the set of leaves of a tree $\mathcal{T}$.

bioRxiv preprint doi: https://doi.org/10.1101/2025.07.27.667007; this version posted July 5, 2026. The copyright holder for this preprint (which was not certified by peer review) is the author/funder, who has granted bioRxiv a license to display the preprint in perpetuity. It is made available under a CC-BY 4.0 International license.

G.1 Mean and variance of parsimony and tPHS

We start with two lemmas, regarding the mean and variance of parsimony and tPHS. The first lemma provides exact expressions for the parsimony mean and variance, assuming a homogeneous tree. The second lemma provides upper bounds for the mean and variance of tPHS in general trees, not necessarily homogeneous. Their proofs appear in Sections I.1 and I.2, respectively.

Lemma 3. *Let $\mathcal{T}_{GT}$ be a homogeneous tree of depth d, whose root node is unmutated. Let $\mathcal{D}$ be the distribution of its set of sequences $\mathcal{S}_{GT}$ of length k, generated according to the non-modifiable model of Section 2.1 with mutation rate λ. Then, taking expectation over $\mathcal{D}$, for $\alpha_d = e^{-\lambda/d} \neq 1/2$,*

$$\mathbb{E}[M(\mathcal{T}_{GT}\mathcal{S}_{GT})] = k \cdot 2^d \cdot \frac{2e^{-\lambda}(1-\alpha_d)}{2\alpha_d - 1} \cdot \left(1 - e^{\lambda} \cdot 2^{-d}\right), \tag{S8}$$

and

$$\mathbb{V}[M(\mathcal{T}_{GT}\mathcal{S}_{GT})] = k \cdot 4^d \cdot \frac{4e^{-2\lambda}(1-\alpha_d)^3}{(2\alpha_d-1)^3} \cdot \left\{1 - \frac{e^{\lambda}(2\alpha_d - 1)\left[2(1-\alpha_d)d + \alpha_d - 2\right] \cdot 2^d - e^{2\lambda}\alpha_d}{2(1-\alpha_d)^2} \cdot 4^{-d}\right\}. \tag{S9}$$

Lemma 4. *Consider the same setting as in Lemma 3, but with an arbitrary tree $\mathcal{T}_{GT}$ (not necessarily homogeneous). Denote by $N = \binom{n}{2}$ its number of leaf pairs. Then, taking expectation over $\mathcal{D}$,*

$$\mathbb{E}[tPHS(\mathcal{T}_{GT}\mathcal{S}_{GT})] \leq k \cdot N\rho^2 q, \tag{S10}$$

and

$$\mathbb{V}[tPHS(\mathcal{T}_{GT}\mathcal{S}_{GT})] < k \cdot N^2\rho^2 q, \tag{S11}$$

where q is the collision probability and ρ is the probability of observing a mutation at a leaf given in (1).

Comparing Lemmas 3 and 4 reveals an important difference between parsimony and tPHS: the parsimony does not depend on the collision probability q. In contrast, the mean and variance of tPHS are linear in q. As a result, in general, a small value of q - which is directly related to the number of homoplasies - increases the statistical power of cPHS; see (16).

Remark 7. Lemma 3 assumes that $\alpha_d \neq 1/2$. In the case $\alpha_d = 1/2$, the mean and variance read

$$\mathbb{E}[M(\mathcal{T}_{\text{GT}}\mathcal{S}_{\text{GT}})] = kd \quad \text{and} \quad \mathbb{V}[M(\mathcal{T}_{\text{GT}}\mathcal{S}_{\text{GT}})] = \frac{k}{12}\left(2d^3 - 9d^2 + 13d\right). \tag{S12}$$

This can be easily verified by plugging $\alpha_d = 1/2$ in the proof of the lemma, or by taking the limit $\alpha_d \to 1/2$ in Eqs. (S8) and (S9).

G.2 The effect of a leaf swap on parsimony and on tPHS

We present two lemmas regarding the mean change in parsimony and tPHS, following a swap of two leaves u, v from different sides of the tree (see Section 5). For convenience, the lemmas consider the change in parsimony and tPHS at a single character. Since both parsimony and tPHS are additive in the sequence length k, the mean change in these quantities is simply k times that of a single character.

Definition 9. [per-character parsimony] Given a full tree $\mathcal{TS}$, for any $i \in [k]$ denote by $M_i(\mathcal{TS})$ the number of mutations in the i-th character across all edges of the tree.

Definition 10. [per-character tPHS] Given a full tree $\mathcal{TS}$, denote by $\text{tPHS}_i(\mathcal{TS})$ the number of leaf pairs with a PHS of 1 at their i-th character, $i \in [k]$:

$$\text{tPHS}_i(\mathcal{TS}) = \frac{1}{2}\left|\{(u,v) \in \mathcal{L}(\mathcal{T}) \mid \text{phs}_i(u,v) = 1\}\right|. \tag{S13}$$

The factor of $1/2$ compensates for double summation of pairs. Note that by definition, the parsimony and tPHS of a tree are the sum of its per-character parsimony and tPHS scores: $M(\mathcal{TS}) = \sum_{i=1}^k M_i(\mathcal{TS})$ and $\text{tPHS}(\mathcal{TS}) = \sum_{i=1}^k \text{tPHS}_i(\mathcal{TS})$, respectively. In addition, $\text{tPHS}_i(\mathcal{TS}) = k \cdot \sum_{(u,v)} \text{phs}_i(u,v)$.

Let us now present the lemmas. Their proofs appear in Appendices I.3 and I.4, respectively.

bioRxiv preprint doi: https://doi.org/10.1101/2025.07.27.667007; this version posted July 5, 2026. The copyright holder for this preprint (which was not certified by peer review) is the author/funder, who has granted bioRxiv a license to display the preprint in perpetuity. It is made available under a CC-BY 4.0 International license.

Lemma 5. *Consider the same setting as in Lemma 3. Further suppose $\lambda \le d/2$. Let $u, v \in \mathcal{L}(\mathcal{T}_{GT})$ be two leaves whose LCA is the tree root. Then, taking expectation over $\mathcal{D}$, for any $i \in [k]$, the expected parsimony change in the i-th character following the leaf swap $u \leftrightarrow v$ is given by*

$$\mathbb{E}\left[M_i\left((\mathcal{TS})_{u\leftrightarrow v}\right) - M_i(\mathcal{T}_{GT}\mathcal{S}_{GT})\right] = 2(1-\rho q)\cdot\left(d - \frac{\rho}{1-\alpha_d}\right). \tag{S14}$$

Lemma 6. *Assume the conditions of Lemma 5. Then, taking expectation over $\mathcal{D}$, the expected tPHS change in the i-th character following the leaf swap $u \leftrightarrow v$ satisfies*

$$\mathbb{E}\left[tPHS_i\left((\mathcal{TS})_{u\leftrightarrow v}\right) - tPHS_i(\mathcal{T}_{GT}\mathcal{S}_{GT})\right] > \frac{2}{3}(1-\alpha_d)(1-\rho q)\cdot\left(4^{d-1}-1\right). \tag{S15}$$

Lemmas 5 and 6 demonstrate a key difference between parsimony and tPHS: Following the leaf swap described above, the mean tPHS value increases *exponentially* with respect to the tree depth d, whereas on average, parsimony increases only linearly.

H Proof of Theorem 1

Proof. First, we prove the lower bound (16) on the normalized change in tPHS due to a leaf swap. Let $\alpha_d = e^{-\lambda/d}$. For any $d \ge 2\lambda$,

$$1-\alpha_d \ge \frac{\lambda}{d} - \frac{1}{2}\left(\frac{\lambda}{d}\right)^2 \ge \frac{\lambda}{d} - \frac{\lambda}{4d} = \frac{3\lambda}{4d}. \tag{S16}$$

Inserting this inequality into (S14) of Lemma 6 yields

$$\mathbb{E}\left[\Delta^P_{u\leftrightarrow v}\right] = k\cdot\left(\mathbb{E}[\text{tPHS}\left((\mathcal{TS})_{u\leftrightarrow v}\right)] - \mathbb{E}[\text{tPHS}(\mathcal{T}_{\text{GT}}\mathcal{S}_{\text{GT}})]\right) > \frac{\lambda k}{2}(1-\rho q)\frac{4^{d-1}-1}{d}. \tag{S17}$$

Next, we apply Lemma 4. Since the tree is homogeneous, its number of leaves is $n = 2^d$. Now, for $d \ge 2$, $N = \binom{n}{2} = \binom{2^d}{2} \le 2(4^{d-1}-1)$. Hence, Eq. (S11) of the lemma gives

$$\sqrt{\mathbb{V}\left[\text{tPHS}(\mathcal{T}_{\text{GT}}\mathcal{S}_{\text{GT}})\right]} < 2\left(4^{d-1}-1\right)\cdot\rho\sqrt{qk}.$$

Equation (16) follows by combining this with (S17).

Next, we prove the upper bound (17) regarding the normalized change in parsimony. Combining the additivity of the parsimony in the sequence length k with (S14) of Lemma 5 regarding the change in parsimony at a single character, gives that

$$\mathbb{E}\left[\Delta^M_{u\leftrightarrow v}\right] = 2(1-\rho q)\left(d - \frac{\rho}{1-\alpha_d}\right)k \le 2(1-\rho q)\left(1-\frac{\rho}{\lambda}\right)dk, \tag{S18}$$

where the inequality above follows from $1-\alpha_d \le \lambda/d$. Next, we apply Lemma 3. For a sufficiently large d, $\alpha_d > 1/2$, and thus satisfies the assumption of the lemma. Further, for a sufficiently large d, the expression in the curly brackets in the RHS of (S9) is larger than 2/3. Hence,

$$\mathbb{V}\left[M(\mathcal{T}_{\text{GT}}\mathcal{S}_{\text{GT}})\right] \ge k\cdot\frac{8(1-\alpha_d)^3 e^{-2\lambda}}{3(2\alpha_d-1)^3}\cdot 4^d \ge \frac{8k}{3}\cdot(1-\alpha_d)^3 e^{-2\lambda}\cdot 4^d,$$

where the second inequality above follows from $\alpha_d \le 1$. Combining this with Eqs. (1) and (S16) yields

$$\sqrt{\mathbb{V}\left[M(\mathcal{T}_{\text{GT}}\mathcal{S}_{\text{GT}})\right]} \ge \sqrt{\frac{8k}{3}}\cdot\left(\frac{3\lambda}{4d}\right)^{3/2} e^{-\lambda}\cdot 2^d > \lambda^{3/2}(1-\rho)\cdot\frac{2^d\sqrt{k}}{\sqrt{d^3}}.$$

Equation (17) follows by combining this with (S18). □

bioRxiv preprint doi: https://doi.org/10.1101/2025.07.27.667007; this version posted July 5, 2026. The copyright holder for this preprint (which was not certified by peer review) is the author/funder, who has granted bioRxiv a license to display the preprint in perpetuity. It is made available under a CC-BY 4.0 International license.

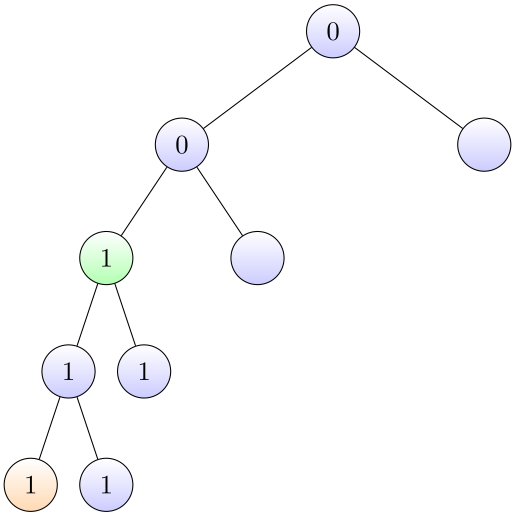

Figure S3: Illustration of the origin of an observed mutation (Def. 11) at a node u (colored in orange) with $k = 1$. The most distant node with the same mutation, $A_1(u)$, is colored in green node. Their edge distance is $E_1(u) = 2$.

I Proofs of Lemmas 3, 4, 5 and 6

To prove the lemmas, we first introduce some definitions. Recall that $\mathcal{L}(\mathcal{T})$ denotes the set of leaves of a tree $\mathcal{T}$. For convenience, for any leaf z define $\mathrm{phs}(z, z) = 0$. In addition, we define the following three quantities regarding the origin of observed mutations, that will be used in our proof. See Fig. S3 for their illustration.

Definition 11. Let $\mathcal{TS}$ be a full tree whose sequences were generated by the non-modifiable mutation process of Section 2.1. Let $u \in \mathcal{L}(\mathcal{T})$ be a leaf in the tree. For each character location i, we denote by $A_i(u)$ the most distant ancestor of u that had a mutation in the i-th character. If either $s_i^{(u)} = 0$ or $s_i^{(u)} \neq 0$ but its father node was unmutated, then we define $A_i(u) = u$. In addition, we denote by $E_i(u)$ the number of edges from $A_i(u)$ to u, and by $\mathcal{T}_{A_i(u)}$ the subtree whose root is $A_i(u)$.

Note that by non-modifiability of the mutation model, $s_i^{(A_i(u))} = s_i^{(u)}$.

I.1 Proof of Lemma 3 (parsimony mean and variance)

The proof of Lemma 3 makes use of the following auxiliary lemma, to be proved shortly.

Lemma 7. *Assume the same setting as in Lemma 3. Let $\mathcal{T}_l$ be a subtree of $\mathcal{T}_{GT}$ of depth $l \leq d$. Let $\mathcal{D}_l$ be the distribution of its set of sequences $\mathcal{S}_l \subseteq \mathcal{S}_{GT}$, conditioned on the event that the sequence at the root node of $\mathcal{T}_l$ is unmutated. For any $i \in [k]$, denote the parsimony in the i-th character of the subtree $\mathcal{T}_l\mathcal{S}_l$ by $M_i^l = M_i(\mathcal{T}_l\mathcal{S}_l)$. Then, taking expectation over $\mathcal{D}_l$,*

$$\mathbb{E}\left[M_i^l\right] = \frac{2(1-\alpha_d)}{2\alpha_d - 1}\left((2\alpha_d)^l - 1\right), \tag{S19}$$

and

$$\mathbb{V}\left[M_i^l\right] = \frac{2(1-\alpha_d)}{(2\alpha_d-1)^3} \cdot \left(2(1-\alpha_d)^2(2\alpha_d)^{2l} - (1-2\alpha_d)(2(1-\alpha_d)l + \alpha_d - 2)(2\alpha_d)^l - \alpha_d\right). \tag{S20}$$

Proof of Lemma 3. By the linearity of expectation, $\mathbb{E}[M(\mathcal{T}_{\mathrm{GT}}\mathcal{S}_{\mathrm{GT}})] = \sum_{i=1}^k \mathbb{E}\left[M_i^d\right]$, where M_i^d is defined in Lemma 7. As characters at different locations $i \in [k]$ evolve independently from each other, $\mathbb{V}[M(\mathcal{T}_{\mathrm{GT}}\mathcal{S}_{\mathrm{GT}})] = \sum_{i=1}^k \mathbb{V}\left[M_i^d\right]$. The lemma follows by taking $l = d$ in Eqs. (S19) and (S20) of Lemma 7. ∎

Proof of Lemma 7. Without loss of generality, we may assume that $i = 1$. To simplify notation, we omit the subscript 1 from M_1^l. We prove the claim by induction on l. For $l = 0$, the subtree consists of only the root, and thus $M_0 = 0$ deterministically. As the RHS of Eqs. (S19) and (S20) vanish at $l = 0$, the induction base is proved.

bioRxiv preprint doi: https://doi.org/10.1101/2025.07.27.667007; this version posted July 5, 2026. The copyright holder for this preprint (which was not certified by peer review) is the author/funder, who has granted bioRxiv a license to display the preprint in perpetuity. It is made available under a CC-BY 4.0 International license.

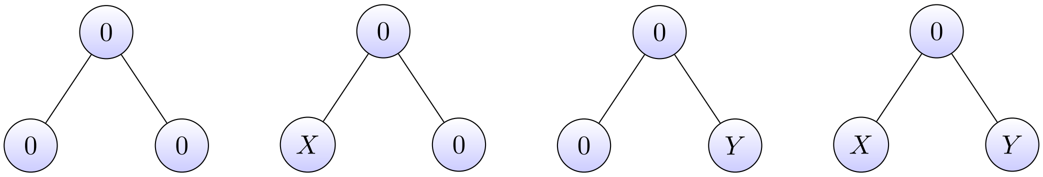

Figure S4: Given an unmutated root, there are four possible scenarios for the states of its two children: (i) no mutation in both branches, (ii) a mutation only in the left branch; (iii) a mutation only in the right branch, and (iv) mutations in both branches. Here $X, Y \in [m]$.

Next, assume that the induction hypothesis holds, namely (S19) and (S20) hold for some $l-1 \geq 0$. We now prove it holds for l. Let $E_l = \mathbb{E}[M^l]$ and $V_l = \mathbb{V}[M^l]$ be the mean and variance, respectively, of the number of mutations in a subtree of depth l whose root has an unmutated sequence. The mean and variance depend only on l and not on the specific subtree, because all subtrees of a homogeneous tree are homogeneous as well. Let $M^{l-1}_{(\text{left})}$ and $M^{l-1}_{(\text{right})}$ be the parsimony scores of the subtrees at the left and right branches of $\mathcal{T}_l\mathcal{S}_l$, respectively, each conditioned on the event that the corresponding subtree roots are unmutated. For future use, note that by the Markovian property of the generative process, $M^{l-1}_{(\text{left})}$ and $M^{l-1}_{(\text{right})}$ are independent given that the root is unmutated.

To analyze E_l and V_l, we split into cases. Let $A \in \{0, 1, 2\}$ be the number of mutations from the root of $\mathcal{T}_l$ to its immediate children; see Figure S4 for an illustration of the different possible events. We first calculate the mean and variance conditioned on a specific value of A. Under the event $A = 0$ (case (i) in Figure S4),

$$\mathbb{E}[M^l \mid A = 0] = \mathbb{E}\left[M^{l-1}_{(\text{left})} + M^{l-1}_{(\text{right})}\right] = 2E_{l-1}, \tag{S21a}$$

and

$$\mathbb{V}[M^l \mid A = 0] = \mathbb{V}\left[M^{l-1}_{(\text{left})} + M^{l-1}_{(\text{right})}\right] = 2V_{l-1}, \tag{S21b}$$

Conditioned on $A = 1$, there are two scenarios: the mutation can be either in the left or in the right branch (cases (ii) and (iii) in Fig. S4, respectively). Due to the non-modifiability of the mutation process, no further mutations can occur in the mutated branch. As a result, $M^l = 1 + M^{l-1}_{(\text{right})}$ in case of a mutation in the left branch, and $M^l = M^{l-1}_{(\text{left})} + 1$ in case of a mutation in the right branch. Since the scenarios of a mutation at the left and at the right branches have equal probabilities,

$$\mathbb{E}[M^l \mid A = 1] = \frac{1}{2}\,\mathbb{E}\left[M^{l-1}_{(\text{left})} + 1\right] + \frac{1}{2}\,\mathbb{E}\left[1 + M^{l-1}_{(\text{right})}\right] = E_{l-1} + 1, \tag{S22a}$$

and

$$\mathbb{V}[M^l \mid A = 1] = \frac{1}{2}\,\mathbb{V}\left[M^{l-1}_{(\text{left})} + 1\right] + \frac{1}{2}\,\mathbb{V}\left[1 + M^{l-1}_{(\text{right})}\right] = V_{l-1}. \tag{S22b}$$

Conditioned on $A = 2$ (case (iv) in Figure S4), due to the non-modifiability of the mutation process,

$$\mathbb{E}[M^l \mid A = 2] = 2 \quad \text{and} \quad \mathbb{V}[M^l \mid A = 2] = 0. \tag{S23}$$

The probability of observing a mutation along a single edge, given that the parent node is unmutated, is $1 - \alpha_d$. By independence of $M^{l-1}_{(\text{left})}$ and $M^{l-1}_{(\text{right})}$, we have that $\mathbb{P}[A = 2] = (1 - \alpha_d)^2$, $\mathbb{P}[A = 1] = 2\alpha_d(1 - \alpha_d)$, and $\mathbb{P}[A = 0] = \alpha_d^2$. Hence, by the law of total expectation,

$$\begin{aligned} E_l &= \sum_{i=0}^{2} \mathbb{P}[A = i] \cdot \mathbb{E}[M^l \mid A = i] \\ &= \alpha_d^2\,\mathbb{E}[M^l \mid A = 0] + 2\alpha_d(1 - \alpha_d)\,\mathbb{E}[M^l \mid A = 1] + (1 - \alpha_d)^2\,\mathbb{E}[M^l \mid A = 2]. \end{aligned}$$

Together with Eqs. (S21a), (S22a) and (S23), we obtain

$$\begin{aligned} E_l &= \alpha_d^2 \cdot 2E_{l-1} + 2\alpha_d(1 - \alpha_d) \cdot (E_{l-1} + 1) + (1 - \alpha_d)^2 \cdot 2 \\ &= 2\left(\alpha_d E_{l-1} + 1 - \alpha_d\right). \end{aligned}$$

bioRxiv preprint doi: https://doi.org/10.1101/2025.07.27.667007; this version posted July 5, 2026. The copyright holder for this preprint (which was not certified by peer review) is the author/funder, who has granted bioRxiv a license to display the preprint in perpetuity. It is made available under a CC-BY 4.0 International license.

Inserting the induction hypothesis (S19) for $l-1$ yields that (S19) hold for l.

Similarly, by the law of total variance,

$$V_l = \mathbb{E}_A[\mathbb{V}[M_l \mid A]] + \mathbb{V}_A[\mathbb{E}[M^l \mid A]].$$

The second term above satisfies

$$\mathbb{V}_A[\mathbb{E}[M^l \mid A]] = \mathbb{E}_A[\mathbb{E}[M^l \mid A]^2] - \mathbb{E}_A[\mathbb{E}[M^l \mid A]]^2 = \sum_{i=0}^{2} \mathbb{P}[A=i]\cdot \mathbb{E}[M^l \mid A=i]^2 - E_l^2,$$

so that

$$V_l = \sum_{i=0}^{2} \mathbb{P}[A=i]\cdot \left(\mathbb{V}[M^l \mid A=i] + \mathbb{E}[M^l \mid A=i]^2\right) - E_l^2.$$

Inserting the expressions derived for the mean and variance conditioned on A, Eqs. (S21), (S22) and (S23) give that

$$\begin{aligned} V_l &= \alpha_d^2\left(2V_{l-1} + 4E_{l-1}^2\right) + 2\alpha_d(1-\alpha_d)\left(V_{l-1} + (E_{l-1}+1)^2\right) + (1-\alpha_d)^2\cdot 4 - E_l^2 \\ &= 2\alpha_d V_{l-1} + 2\alpha_d(1+\alpha_d)E_{l-1}^2 + 4\alpha_d(1-\alpha_d)E_{l-1} + 2(2-3\alpha_d+\alpha_d^2) - E_l^2 \\ &= 2\alpha_d V_{l-1} + \frac{2\alpha_d(1-\alpha_d)}{(1-2\alpha_d)^2}\left(2(1-\alpha_d)(2\alpha_d)^{l-1} - 1\right)^2, \end{aligned}$$

where the last equality follows from (S19). Plugging in the induction hypothesis (S20) at $l-1$ yields (S20) at l. □

I.2 Proof of Lemma 4 (PHS mean and variance)

Proof. Let $u, v \in \mathcal{L}(\mathcal{T}_{\mathrm{GT}})$ be a pair of leaves in the tree, and denote their LCA by w. Let $\tau_w \in [0,1)$ be the birth time of w. By Lemma 1, $k\cdot \mathrm{phs}(u,v)$ follows a Binomial distribution with k trials and success probability $p = e^{-\lambda\tau_w}\left(1-e^{-\lambda(1-\tau_w)}\right)^2 q$. Hence, using (1),

$$\mathbb{E}[k\cdot \mathrm{phs}(u,v)] = k\cdot p \le (1-e^{-\lambda})^2 q = k\cdot \rho^2 q.$$

Equation (S10) of the lemma follows from the definition of tPHS (14) and the linearity of expectation.

Next, let us consider the variance. By (10), for a single pair of leaves,

$$\mathbb{V}[k\cdot \mathrm{phs}(u,v)] = k\cdot p(1-p) < k\cdot p \le k\cdot \rho^2 q.$$

Equation (S11) follows from the fact that $\mathbb{V}\left[\sum_{i=1}^{N} X_i\right] \le N^2\,\mathbb{V}[X_1]$ for any collection of identically distributed random variables X_i. □

I.3 Proofs of Lemma 5 (mean parsimony change following a leaf swap)

For a fixed tree $\mathcal{T}_{\mathrm{GT}}$, the change in parsimony following a leaf swap between u, v is a random variable that depends only on the random quantities $E_i(u)$ and $E_i(v)$, defined in Def. 11. Indeed, as the following auxiliary lemma shows, conditional on $E_i(u)$ and $E_i(v)$, the change is deterministic. The proof of Lemma 5 follows by combining this result with Lemma 9 below, which characterizes the distribution of $E_i(u)$. The proofs of these auxiliary lemmas appear in Appendices I.5 and I.6.

Lemma 8. *Let $\mathcal{T}_{GT}\mathcal{S}_{GT}$ be a full tree, and let $u, v \in \mathcal{L}(\mathcal{T}_{GT})$ be two leaves whose LCA is the tree root. Then, for any $i \in [k]$, the parsimony change in the i-th character following the leaf swap $u \leftrightarrow v$ is given by*

$$M_i\left((\mathcal{T}\mathcal{S})_{u\leftrightarrow v}\right) - M_i(\mathcal{T}_{GT}\mathcal{S}_{GT}) = \begin{cases} 0, & s_i^{(u)} = s_i^{(v)}, \\ E_i(u) + E_i(v), & s_i^{(u)} \ne s_i^{(v)}. \end{cases} \tag{S24}$$

Lemma 9. *Let $\mathcal{T}_{GT}\mathcal{S}_{GT}$ be a full tree with a homogeneous topology of depth d, generated according to the non-modifiable model of Section 2.1 with a mutation rate λ. Denote $\rho = 1-e^{-\lambda}$ as in (1), and $\alpha_d = e^{-\lambda/d}$. Let u be a leaf in the tree. For any $i \in [k]$, if the i-th character of u is unmutated, $s_i^{(u)} = 0$, then $E_i(u) = 0$. Otherwise, its distribution is given by*

$$\mathbb{P}\left[E_i(u) = t \mid s_i^{(u)} > 0\right] = \frac{1}{\rho}\alpha_d^{d-t-1}(1-\alpha_d), \qquad \text{for } 0 \le t \le d-1. \tag{S25}$$

bioRxiv preprint doi: https://doi.org/10.1101/2025.07.27.667007; this version posted July 5, 2026. The copyright holder for this preprint (which was not certified by peer review) is the author/funder, who has granted bioRxiv a license to display the preprint in perpetuity. It is made available under a CC-BY 4.0 International license.

We now prove the lemma.

Proof of Lemma 5. Denote $s_u = s_i^{(u)}$ and $s_v = s_i^{(v)}$. Let $\Delta = M_i\left((\mathcal{TS})_{u\leftrightarrow v})\right) - M_i(\mathcal{T}_{\mathrm{GT}}\mathcal{S}_{\mathrm{GT}})$. The quantity of interest is $\mathbb{E}[\Delta]$. To this end, denote the event $A = \{s_u \neq s_v\}$. Under A^c, $\Delta = 0$. Hence,

$$\mathbb{E}[\Delta] = \mathbb{P}[A] \cdot \mathbb{E}[\Delta \mid A].$$

Next, we split A into three cases, depending on whether s_u or s_v were mutated, or both:

$$A_1 = \{s_u \neq 0 \wedge s_v \neq 0 \wedge s_u \neq s_v\}, \quad A_2 = \{s_u = 0 \wedge s_v \neq 0\}, \quad \text{and} \quad A_3 = \{s_u \neq 0 \wedge s_v = 0\}.$$

By a symmetry argument, $\mathbb{P}[A_2 \mid A] = \mathbb{P}[A_3 \mid A]$ and $\mathbb{E}[\Delta \mid A_2] = \mathbb{E}[\Delta \mid A_3]$. Hence,

$$\mathbb{E}[\Delta] = \mathbb{P}[A_1] \cdot \mathbb{E}[\Delta \mid A_1] + 2\,\mathbb{P}[A_2] \cdot \mathbb{E}[\Delta \mid A_2]. \tag{S26}$$

Next, we compute the probabilities in the equation above. Since the LCA of u and v is the root, which is unmutated by construction, s_u and s_v are independent random variables. We thus obtain

$$\mathbb{P}[A_1] = \rho^2(1-q) \quad \text{and} \quad \mathbb{P}[A_2] = \rho(1-\rho).$$

Inserting this into (S26) yields

$$\mathbb{E}[\Delta] = \rho^2(1-q) \cdot \mathbb{E}[\Delta \mid A_1] + 2(1-\rho)\rho \cdot \mathbb{E}[\Delta \mid A_2]. \tag{S27}$$

Next, we compute the conditional expectations. Denote $e_u = E_i(u)$ and $e_v = E_i(v)$. By combining this with the law of total expectation and (S24) of Lemma 8,

$$\begin{aligned}\mathbb{E}[\Delta \mid A_2] &= \sum_{e_u,e_v=0}^{d-1} \mathbb{P}[(e_u,e_v) \mid A_2] \cdot \mathbb{E}\left[\Delta \mid (e_u,e_v) \wedge A_2\right] \\ &= \sum_{e_u,e_v=0}^{d-1} \mathbb{P}[e_u \mid s_u = 0] \cdot \mathbb{P}[e_v \mid s_v > 0] \cdot (e_u + e_v)\,.\end{aligned}$$

Since $s_u = 0$, $E_i(u) = 0$ follows by definition. Hence,

$$\mathbb{E}[\Delta \mid A_2] = \sum_{e_v=0}^{d-1} \mathbb{P}[e_v \mid s_v > 0] \cdot e_v.$$

Combining this with (S25) of Lemma 9, we obtain

$$\begin{aligned}\mathbb{E}[\Delta \mid A_2] &= \frac{1}{\rho}\alpha_d^{d-1}(1-\alpha_d) \cdot \sum_{e_v=0}^{d-1} \alpha_d^{-e_v} e_v \\ &= \frac{1}{\rho}\alpha_d^{d-1}(1-\alpha_d) \cdot \alpha_d^{1-d} \cdot \frac{d(1-\alpha_d) + e^{-\lambda} - 1}{(1-\alpha_d)^2} \\ &= \frac{1}{\rho} \cdot \frac{d(1-\alpha_d) - \rho}{1-\alpha_d} = \frac{d}{\rho} - \frac{1}{1-\alpha_d}.\end{aligned} \tag{S28}$$

By similar arguments,

$$\begin{aligned}\mathbb{E}[\Delta \mid A_1] &= \sum_{e_u,e_v=0}^{d-1} \mathbb{P}[e_u \mid s_u > 0] \cdot \mathbb{P}[e_v \mid s_v > 0] \cdot (e_u + e_v) \\ &= \frac{1}{\rho^2}\alpha_d^{2d-2}(1-\alpha_d)^2 \sum_{e_u,e_v=0}^{d-1} \alpha_d^{-e_u-e_v}(e_u + e_v).\end{aligned} \tag{S29}$$

bioRxiv preprint doi: https://doi.org/10.1101/2025.07.27.667007; this version posted July 5, 2026. The copyright holder for this preprint (which was not certified by peer review) is the author/funder, who has granted bioRxiv a license to display the preprint in perpetuity. It is made available under a CC-BY 4.0 International license.

Let us calculate the sum in the RHS of (S29),

$$\begin{aligned}\sum_{e_u,e_v=0}^{d-1} \alpha_d^{-e_u-e_v}(e_u+e_v) &= \sum_{e_u=0}^{d-1} \alpha_d^{-e_u}\left(e_u \sum_{e_v=0}^{d-1} \alpha_d^{-e_v} + \sum_{e_v=0}^{d-1} \alpha_d^{-e_v} e_v\right)\\ &= \sum_{e_u=0}^{d-1} \alpha_d^{-e_u}\left(e_u \cdot \alpha_d^{1-d}\frac{1-e^{-\lambda}}{1-\alpha_d} + \alpha_d^{1-d}\cdot \frac{d(1-\alpha_d)+e^{-\lambda}-1}{(1-\alpha_d)^2}\right)\\ &= \alpha_d^{1-d}\sum_{e_u=0}^{d-1} \alpha_d^{-e_u}\left(e_u \cdot \frac{\rho}{1-\alpha_d} + \frac{d(1-\alpha_d)-\rho}{(1-\alpha_d)^2}\right)\\ &= \alpha_d^{1-d}\left[\left(\alpha_d^{1-d}\cdot\frac{d(1-\alpha_d)-\rho}{(1-\alpha_d)^2}\cdot\frac{\rho}{1-\alpha_d} + \alpha_d^{1-d}\frac{\rho}{1-\alpha_d}\cdot\frac{d(1-\alpha_d)-\rho}{(1-\alpha_d)^2}\right)\right]\\ &= \frac{2\alpha_d^{2-2d}\rho}{(1-\alpha_d)^3}\cdot[d(1-\alpha_d)-\rho] = \frac{2\alpha_d^{2-2d}\rho}{(1-\alpha_d)^2}\cdot d - \frac{2\alpha_d^{2-2d}\rho^2}{(1-\alpha_d)^3}.\end{aligned}$$

Plugging this into (S29) gives that

$$\mathbb{E}[\Delta \mid A_1] = \frac{2d}{\rho} - \frac{2}{1-\alpha_d}. \tag{S30}$$

The lemma follows by combining (S27) with (S28) and (S30). □

I.4 Proof of Lemma 6 (mean tPHS change following a leaf swap)

Similar to parsimony, the change in tPHS following a leaf swap between u, v is a random variable that depends only on the two random quantities $E_i(u)$ and $E_i(v)$, defined in Def. 11. Indeed, as the following auxiliary lemma shows, conditional on $E_i(u)$ and $E_i(v)$, the change is deterministic. The proof of Lemma 6 follows by combining this result with Lemma 9 above, which characterizes the distribution of $E_i(u)$. The proof Lemma 10 appears in Appendix I.5.

Lemma 10. *Let $\mathcal{T}_{GT}\mathcal{S}_{GT}$ be a homogeneous full tree, and let $u, v \in \mathcal{L}(\mathcal{T}_{GT})$ be two leaves whose LCA is the tree root. Suppose that either $s_i^{(u)} \neq s_i^{(v)}$ or $s_i^{(u)} = s_i^{(v)} = 0$. Then, for any $i \in [k]$,*

$$tPHS_i\left((\mathcal{TS})_{u\leftrightarrow v}\right) - tPHS_i(\mathcal{T}_{GT}\mathcal{S}_{GT}) = \frac{1}{3}\left(4^{E_i(u)} + 4^{E_i(v)} - 2\right). \tag{S31}$$

We now prove the lemma.

Proof of Lemma 6. Denote $s_u = s_i^{(u)}$ and $s_v = s_i^{(v)}$ By similar arguments as in the proof of Lemma 5, (S27) holds for $\Delta = \mathrm{tPHS}_i\left((\mathcal{TS})_{u\leftrightarrow v}\right) - \mathrm{tPHS}_i(\mathcal{T}_{\mathrm{GT}}\mathcal{S}_{\mathrm{GT}})$ as well. That is,

$$\mathbb{E}[\Delta] = \rho^2(1-q)\cdot\mathbb{E}[\Delta \mid A_1] + 2(1-\rho)\rho\cdot\mathbb{E}[\Delta \mid A_2], \tag{S32}$$

where $A_1 = \{s_u \neq 0 \wedge s_v \neq 0 \wedge s_u \neq s_v\}$ and $A_2 = \{s_u = 0 \wedge s_v \neq 0\}$. Since u and v are from different sides of the tree, s_u and s_v are independent, as well as e_u and e_v. By combining this with the law of total expectation and (S31) of Lemma 10,

$$\begin{aligned}\mathbb{E}[\Delta \mid A_1] &= \sum_{e_u,e_v=0}^{d-1} \mathbb{P}[E_i(u)=e_u \mid A_1]\,\mathbb{P}[E_i(v)=e_v \mid A_1]\cdot\mathbb{E}[\Delta \mid A_1\cup\{e_u,e_v\}]\\ &= \frac{1}{3}\sum_{e_u,e_v=0}^{d-1} \mathbb{P}[E_i(u)=e_u \mid s_u>0]\,\mathbb{P}[E_i(v)=e_v \mid s_v>0]\cdot\left(4^{e_u}+4^{e_v}-2\right).\end{aligned}$$

Invoking Lemma 9 gives that

$$\begin{aligned}\mathbb{E}[\Delta \mid A_1] &= \frac{1}{3}\sum_{e_u,e_v=0}^{d-1} \frac{1}{\rho}\alpha_d^{d-e_u-1}(1-\alpha_d)\cdot\frac{1}{\rho}\alpha_d^{d-e_v-1}(1-\alpha_d)\cdot\left(4^{e_u}+4^{e_v}-2\right)\\ &= \frac{1}{3\rho^2}\alpha_d^{2d-2}(1-\alpha_d)^2\cdot\sum_{e_u,e_v=0}^{d-1}\alpha_d^{-e_u-e_v}\cdot\left(4^{e_u}+4^{e_v}-2\right).\end{aligned} \tag{S33}$$

bioRxiv preprint doi: https://doi.org/10.1101/2025.07.27.667007; this version posted July 5, 2026. The copyright holder for this preprint (which was not certified by peer review) is the author/funder, who has granted bioRxiv a license to display the preprint in perpetuity. It is made available under a CC-BY 4.0 International license.

Observe that

$$\sum_{e_u,e_v=0}^{d-1} \alpha_d^{-e_u-e_v} = \left(\sum_{e_u=0}^{d-1} \alpha_d^{-e_u}\right)^2 = \left(\alpha_d^{1-d} \cdot \frac{1-\alpha_d^d}{1-\alpha_d}\right)^2 = \alpha_d^{2-2d} \cdot \frac{\rho^2}{(1-\alpha_d)^2},$$

$$\sum_{e_u,e_v=0}^{d-1} \alpha_d^{-e_u-e_v} \cdot 4^{e_u} = \sum_{e_u=0}^{d-1} \alpha_d^{-e_u} \cdot 4^{e_u} \sum_{e_v=0}^{d-1} \alpha_d^{-e_v} = \alpha_d^{1-d} \cdot \frac{4^d-\alpha_d^d}{4-\alpha_d} \cdot \alpha_d^{1-d} \cdot \frac{1-\alpha_d^d}{1-\alpha_d} = \alpha_d^{2-2d} \cdot \frac{4^d-\alpha_d^d}{4-\alpha_d} \cdot \frac{\rho}{1-\alpha_d}.$$

By symmetry, $\sum_{e_u,e_v=0}^{d-1} \alpha_d^{-e_u-e_v} \cdot 4^{e_v} = \sum_{e_u,e_v=0}^{d-1} \alpha_d^{-e_u-e_v} \cdot 4^{e_u}$. Inserting these expression into (S33) yields

$$\begin{aligned}\mathbb{E}\left[\Delta \mid A_1\right] &= \frac{1}{3\rho^2}(1-\alpha_d)^2 \cdot \left(\frac{\rho^2}{(1-\alpha_d)^2} + 2 \cdot \frac{4^d-e^{-\lambda}}{4-\alpha_d} \cdot \frac{\rho}{1-\alpha_d}\right)\\ &> \frac{2}{3\rho^2}(1-\alpha_d)^2 \cdot \frac{4^d-e^{-\lambda}}{4-\alpha_d} \cdot \frac{\rho}{1-\alpha_d}\\ &= \frac{2}{3\rho}(1-\alpha_d) \cdot \frac{4^d-e^{-\lambda}}{4-\alpha_d} > \frac{2}{3\rho}(1-\alpha_d) \cdot \left(4^{d-1}-1\right).\end{aligned}$$

Similarly, by combining the law of total expectation, (S31) of Lemma 10 and (S25) of Lemma 9,

$$\begin{aligned}\mathbb{E}\left[\Delta \mid A_2\right] &= \frac{1}{3}\sum_{e_v=0}^{d-1} \mathbb{P}\left[E_i(v)=e_v \mid s_v>0\right] \cdot (4^{e_v}-1) = \frac{1}{3\rho}\alpha_d^{d-1}(1-\alpha_d) \cdot \sum_{e_v=0}^{d-1} \alpha_d^{-e_v} \cdot (4^{e_v}-1)\\ &= \frac{1}{3\rho}\alpha_d^{d-1}(1-\alpha_d) \cdot \left(\alpha_d^{1-d} \cdot \frac{4^d-e^{-\lambda}}{4-\alpha_d} + \alpha_d^{1-d}\frac{\rho}{1-\alpha_d}\right)\\ &> \frac{1}{3\rho}(1-\alpha_d) \cdot \frac{4^d-1}{4} > \frac{1}{3\rho}(1-\alpha_d) \cdot \left(4^{d-1}-1\right).\end{aligned}$$

Inserting the last two results into (S32) gives (S14) and completes the proof of the lemma. □

I.5 Proofs of Lemmas 8 and 10 (deterministic parsimony and PHS change following a leaf swap)

The proofs of the lemmas use the following two propositions regarding the quantities defined in Def. 11.

Proposition 1. *Let $\mathcal{TS}$ be a full tree, and let u be a leaf in the tree. Let $i \in [k]$. Then for any $z \in \mathcal{T}_{A_i(u)}$,*

$$s_i^{(z)} = s_i^{(u)}. \tag{S34}$$

In addition, let w be the parent of $A_i(u)$. Then

$$s_i^{(w)} = 0. \tag{S35}$$

Proof. To simplify the notations, for any node z, denote $s_z = s_i^{(z)}$. Let $z \in \mathcal{T}_{A_i(u)}$. There are two cases to consider: $s_u = 0$ or $s_u \neq 0$. If $s_u = 0$, then by non-modifiability all ancestors of u are unmutated. Hence, $A_i(u) = u$, and $T_{A_i(u)} = \{u\}$. In this case, (S34) trivially holds. Next, suppose $s_u \neq 0$. Since $A_i(u)$ is an ancestor of u, whose character is mutated, by the non-modifiability $s_{A_i(u)} = s_u$. Since any $z \in \mathcal{T}_{A_i(u)}$ is either $A_i(u)$ or an offspring of $A_i(u)$, (S34) follows by the non-modifiability. Finally, (S35) follows from the definition of $A_i(u)$ as the most distant mutated ancestor of u. □

Proposition 2. *Let $\mathcal{T}_{\text{GT}}\mathcal{S}_{\text{GT}}$ be a full tree, and let u be a leaf in the tree. Let $i \in [k]$, and denote $x = s_i^{(u)}$. Let $\mathcal{TS}$ be the tree after setting $s_i^{(u)} = y \neq x$ and correcting for non-modifiability (cf. Def. 6). Then, for all ancestors z of u that belong to $\mathcal{T}_{A_i(u)}$ it holds that in the original tree $s_i^{(z)} = x$, whereas in the modified tree $\mathcal{TS}$, $s_i^{(z)} = 0$. The sequences at all the other nodes, except u, are the same in $\mathcal{T}_{\text{GT}}\mathcal{S}_{\text{GT}}$ and $\mathcal{TS}$.*

Proof. Let $i \in [k]$. If $\mathcal{T}_{A_i(u)} = \{u\}$, then the lemma holds trivially. Hence, we assume that there is at least one ancestor of u in $\mathcal{T}_{A_i(u)}$, namely $w \in \mathcal{T}_{A_i(u)}$ where w is the parent of u. For convenience, for any node z denote $s_z = s_i^{(z)}$. By Proposition 1, $s_z = x$ for any $z \in \mathcal{T}_{A_i(u)}$ in the original tree $\mathcal{T}_{\text{GT}}\mathcal{S}_{\text{GT}}$. In the modified tree $\mathcal{TS}$, by definition, $s_u = y$. Since $w \in \mathcal{T}_{A_i(u)}$, the sibling of u, denoted $\bar{u}$, satisfies $s_{\bar{u}} = x \neq y$. Hence, by non-modifiability, $s_w = 0$ in the modified tree. As a result, all ancestors of u are corrected from x to 0. Since violations of non-modifiability can only occur from the modified leaf and upwards, the proof is complete. □

bioRxiv preprint doi: https://doi.org/10.1101/2025.07.27.667007; this version posted July 5, 2026. The copyright holder for this preprint (which was not certified by peer review) is the author/funder, who has granted bioRxiv a license to display the preprint in perpetuity. It is made available under a CC-BY 4.0 International license.

We now prove the lemmas.

Proof of Lemma 8. For any node z, denote by $s_z = s_i^{(z)}$ the state of the i-th character of z in $\mathcal{T}_{\text{GT}}\mathcal{S}_{\text{GT}}$, and by s'_z its state in $(\mathcal{TS}_{u\leftrightarrow v})$. Denote $\Delta = M_i\left((\mathcal{TS})_{u\leftrightarrow v}\right) - M_i(\mathcal{T}_{\text{GT}}\mathcal{S}_{\text{GT}})$.

If $s_u = s_v$, then $(\mathcal{TS})_{u\leftrightarrow v} = \mathcal{T}_{\text{GT}}\mathcal{S}_{\text{GT}}$, so $\Delta = 0$ and (S24) holds. Next, we analyze the case $s_u \neq s_v$. Since u and v are from different sides of the tree and the root sequence is fixed with unmutated states, we can decompose the parsimony change as

$$\Delta = \Delta_u + \Delta_v, \tag{S36}$$

where Δ_u and Δ_v are the parsimony changes due to the modification of s_u (while keeping s_v unchanged) and of s_v (while keeping s_u unchanged), respectively.

Let us begin by calculating Δ_u. Denote $a_u = A_i(u)$ and $e_u = E_i^{(u)}$. We split into cases: $e_u > 0$ and $e_u = 0$. First, suppose that $e_u > 0$. According to Proposition 2, the only inner nodes whose sequences are modified following the swap are ancestors z of u that satisfy $z \in \mathcal{T}_{a_u}$. Specifically, they are modified from s_u to 0. Hence, for each such node z, there is a new mutation along the branch to the child which is not u or one of its ancestors. The number of these nodes z is $E_i(u)$. In addition, since $s'_{a_u} = 0$, there is no longer a mutation from a_u's parent to a_u. Finally, we need to ask if there is a new mutation from w, the parent of u, to u. Since $s'_u = s_v$, if $s_v = 0$ then there is no such mutation. Suppose now $s_v > 0$. Since $e_u > 0$, $w \in \mathcal{T}_{a_u}$, and thus $s'_w = 0$. Since $s'_u = s_v > 0$, there is a new mutation from w to u. In total, we obtain that if $e_u > 0$, then

$$\Delta_u = e_u - 1 + 1_{\{s_v > 0\}},$$

where $1_{\{s_v>0\}}$ is the indicator of the event $\{s_v > 0\}$. Under the assumption $e_u > 0$, we have $1_{\{s_u>0\}} = 1$. Hence, we can write

$$\Delta_u = e_u - 1_{\{s_u>0\}} + 1_{\{s_v>0\}}. \tag{S37}$$

Next, suppose that $e_u = 0$. Then, by definition of e_u, $s_w = 0$. In addition, since $w \notin \mathcal{T}_{a_u}$, Proposition 2 implies that $s'_w = 0$. Recall that $s'_u = s_v$. Let us split into three cases: (i) $s_u = s_v$, (ii) $s_u = 0$ and $s_v > 0$, and (iii) $s_u > 0$ and $s_v = 0$. In case (i), $s_u = s'_u$ and $\Delta_u = 0$. In case (ii), $s_u < s'_u$ and $\Delta_u = 1$. Finally, in case (iii), $s_u > s'_u$ and $\Delta_u = -1$. It can be thus verified that (S37) holds also in the case $e_u = 0$.

By similar arguments, one can show that

$$\Delta_v = e_v - 1_{\{s_v>0\}} + 1_{\{s_u>0\}}$$

where $e_v = E_i^{(v)}$ is either 0 or greater than 0. Inserting this together with (S37) into (S36) yields (S24). □

In the following proof, we denote the PHS of a leaf pair $z, \tilde{z}$ before and after the swap by $\text{phs}(z,\tilde{z}) = \text{phs}\left(z,\tilde{z} \mid \mathcal{T}_{\text{GT}}\mathcal{S}_{\text{GT}}\right)$ and $\text{phs}'(z,\tilde{z}) = \text{phs}\left(z,\tilde{z} \mid (\mathcal{TS})_{u\leftrightarrow v}\right)$, respectively.

Proof of Lemma 10. Let $i \in [k]$. For any node z, denote by $s_z = s_i^{(z)}$ the state of the i-th character of $s^{(z)}$ in $\mathcal{T}_{\text{GT}}\mathcal{S}_{\text{GT}}$, and by s'_z its state in $(\mathcal{TS})_{u\leftrightarrow v}$. Denote $\Delta = \text{tPHS}_i\left((\mathcal{TS})_{u\leftrightarrow v}\right) - \text{tPHS}_i(\mathcal{T}_{\text{GT}}\mathcal{S}_{\text{GT}})$. Further denote $x = s_u$ and $y = s_v$.

If $x = y$, then $(\mathcal{TS})_{u\leftrightarrow v} = \mathcal{T}_{\text{GT}}\mathcal{S}_{\text{GT}}$, in which case the LHS of (S31) vanishes. Since, by assumption, $x = y = 0$, $E_i(u) = E_i(v) = 0$, and the RHS of (S31) vanishes as well. Next, we analyze the case $x \neq y$. For convenience, we denote by $\Delta(z,\tilde{z})$ the contribution of a specific pair of leaves $z, \tilde{z} \in \mathcal{L}(\mathcal{T}_{\text{GT}})$ at their i-th character to the total PHS change Δ,

$$\Delta(z,\tilde{z}) = \text{phs}_i\left(z,\tilde{z} \mid (\mathcal{TS})_{u\leftrightarrow v}\right) - \text{phs}_i\left(z,\tilde{z} \mid \mathcal{T}_{\text{GT}}\mathcal{S}_{\text{GT}}\right).$$

Let $U = \mathcal{L}(\mathcal{T}_{A_i(u)})$ and $V = \mathcal{L}(\mathcal{T}_{A_i(v)})$ be the sets of tree leaves whose LCA is $A_i(u)$ and $A_i(v)$, respectively. Further let $W = U \cup V$ and $R = \mathcal{L}(\mathcal{T}_{\text{GT}}) \setminus W$. Then

$$\begin{aligned}\mathcal{L}(\mathcal{T}_{\text{GT}}) \times \mathcal{L}(\mathcal{T}_{\text{GT}}) &= (R \times R) \cup (R \times W) \cup (W \times R) \cup (W \times W) \\ &= (R \times R) \cup (R \times W) \cup (W \times R) \cup (U \times V) \cup (V \times U) \cup (U \times U) \cup (V \times V),\end{aligned}$$

bioRxiv preprint doi: https://doi.org/10.1101/2025.07.27.667007; this version posted July 5, 2026. The copyright holder for this preprint (which was not certified by peer review) is the author/funder, who has granted bioRxiv a license to display the preprint in perpetuity. It is made available under a CC-BY 4.0 International license.

where the union is over disjoint sets. Since for $\Delta(z,\tilde{z}) = \Delta(\tilde{z},z)$ for any $z,\tilde{z}$, we can decompose the total PHS change as

$$\Delta = \frac{1}{2}\sum_{z,\tilde{z}\in R}\Delta(z,\tilde{z}) + \sum_{z\in W,\tilde{z}\in R}\Delta(z,\tilde{z}) + \sum_{z\in U,\tilde{z}\in V}\Delta(z,\tilde{z}) + \frac{1}{2}\sum_{z,\tilde{z}\in U}\Delta(z,\tilde{z}) + \frac{1}{2}\sum_{z,\tilde{z}\in V}\Delta(z,\tilde{z}).$$

Let $U^* = U \setminus \{u\}$ and $V^* = V \setminus \{v\}$. Then $U \times V = (U^* \times V^*) \cup (\{u\} \times V^*) \cup (U^* \times \{v\}) \cup (\{u\} \times \{v\})$, $U \times U = (U^* \times U^*) \cup (\{u\} \times U^*) \cup (U^* \times \{u\}) \cup (\{u\} \times \{u\})$, and similarly for $V \times V$. Therefore,

$$\begin{aligned}\Delta = &\frac{1}{2}\sum_{z,\tilde{z}\in R}\Delta(z,\tilde{z}) + \sum_{z\in W,\tilde{z}\in R}\Delta(z,\tilde{z}) + \sum_{z\in U^*,\tilde{z}\in V^*}\Delta(z,\tilde{z}) + \frac{1}{2}\sum_{z,\tilde{z}\in U^*}\Delta(z,\tilde{z}) + \frac{1}{2}\sum_{z,\tilde{z}\in V^*}\Delta(z,\tilde{z}) \\ &+ \sum_{z\in U^*\cup V^*}\left(\Delta(z,u) + \Delta(z,v)\right) + \Delta(u,v),\end{aligned} \tag{S38}$$

where we used the fact that by definition, $\Delta(u,u) = \Delta(v,v) = 0$.

Let us analyze each of the seven terms in the RHS of (S38). First, we show that the first two terms vanish, since

$$\Delta(z,\tilde{z}) = 0, \quad \forall z \in \mathcal{L}(\mathcal{T}_{\mathrm{GT}}) \text{ and } \tilde{z} \in R. \tag{S39}$$

To prove (S39), let $\tilde{z} \in R$, and consider three possible cases for the leaf z: either $z \in R$, $z \in U$, or $z \in V$. If $z \in R$, then also $\mathrm{LCA}(z,\tilde{z}) \in R$. By Proposition 2, the sequences at both $z,\tilde{z}$, and $\mathrm{LCA}(z,\tilde{z})$ are not affected by the leaf swap, and thus $\Delta(z,\tilde{z}) = 0$. Next, suppose that $z \in U$. By Proposition 1, $s_{\mathrm{LCA}(z,\tilde{z})} = 0$. By Proposition 2, also $s'_{\mathrm{LCA}(z,\tilde{z})} = 0$, that is $s'_{\mathrm{LCA}(z,\tilde{z})} = s_{\mathrm{LCA}(z,\tilde{z})}$. Hence $\Delta(z,\tilde{z}) = 0$ also in this case. The case $z \in V$ is similar to $z \in U$. Therefore, (S39) is proved.

Next, we show that the third term in the RHS of (S38) also vanishes, since

$$\sum_{z\in U^*,\tilde{z}\in V^*}\Delta(z,\tilde{z}) = 0, \quad \forall z \in U^* \text{ and } \tilde{z} \in V^*. \tag{S40}$$

This equality follows trivially by the fact that $s_z = x \neq y = s_{\tilde{z}}$ for any $z \in U^*$ and $\tilde{z} \in V^*$.

Next, we show that the fourth term in the RHS of (S38) satisfies

$$\frac{1}{2}\sum_{z,\tilde{z}\in U}\Delta(z,\tilde{z}) = \frac{1}{3}\left(4^{e_u} - 3\cdot 2^{e_u} + 2\right). \tag{S41}$$

Let $z,\tilde{z} \in U$. First, let us show that in the unmodified tree, $\mathrm{phs}(z,\tilde{z}) = 0$. If $x = 0$, then this holds by definition of phs. Next, suppose that $x \neq 0$. Since $\mathrm{LCA}(z,\tilde{z}) \in \mathcal{T}_{\mathrm{u}}$, Proposition 1 implies that $s_{\mathrm{LCA}(z,\tilde{z})} = x \neq 0$. Hence $\mathrm{phs}(z,\tilde{z}) = 0$ also in this case. Next, to analyze $\mathrm{phs}(z,\tilde{z})'$ in the modified tree, let B be the set of ancestors of u which are in $\mathcal{T}_{\mathrm{u}}$. By (S31) of Lemma 10, these ancestors $b \in B$ are the only inner nodes whose states at location i get modified. Specifically, they satisfy $s'_b = 0$. Hence, $\mathrm{phs}(z,\tilde{z})' = 1$ if $\mathrm{LCA}(z,\tilde{z}) \in B$, and $\mathrm{phs}(z,\tilde{z})' = 0$ otherwise. Let us calculate the number of pairs $(z,\tilde{z})$ whose LCA is in B. Denote $e_u = E_i^{(u)}$, and let $B = (b_1,\ldots,b_{e_u})$, where b_{l+1} is the parent of b_l, and b_1 is the parent of u. Observe that b_l has 2^{l-1} leaves in each of its branches. However, since u is in one of the branches and $z,\tilde{z} \neq u$, there are only 2^{l-1} relevant leaves in one of the branches. The total number of pairs is thus

$$\sum_{l=1}^{e_u} 2^{l-1}\cdot\left(2^{l-1} - 1\right) = \frac{1}{3}\left(4^{e_u} - 3\cdot 2^{e_u} + 2\right).$$

This proves (S41). Similarly, for the fifth term in the RHS of (S38),

$$\frac{1}{2}\sum_{z,\tilde{z}\in V^*}\Delta(z,\tilde{z}) = \frac{1}{3}\left(4^{e_v} - 3\cdot 2^{e_v} + 2\right). \tag{S42}$$

Next, we show that the last term in the RHS of (S38) vanishes,

$$\Delta(u,v) = 0. \tag{S43}$$

This follows from $s_u = x \neq y = s_v$, which implies that $\mathrm{phs}(u,v) = \mathrm{phs}'(u,v) = 0$.

bioRxiv preprint doi: https://doi.org/10.1101/2025.07.27.667007; this version posted July 5, 2026. The copyright holder for this preprint (which was not certified by peer review) is the author/funder, who has granted bioRxiv a license to display the preprint in perpetuity. It is made available under a CC-BY 4.0 International license.

Finally, we show that the sixth term in the RHS of (S38) satisfies

$$\sum_{z \in U^* \cup V^*} (\Delta(z,u) + \Delta(z,v)) = 2^{e_u} + 2^{e_v} - 2. \tag{S44}$$

Observe that

$$\begin{aligned}\sum_{z \in U^* \cup V^*} (\Delta(z,u) + \Delta(z,v)) &= \sum_{z \in U^*} \left[\mathrm{phs}'(z,u) - \mathrm{phs}(z,u)\right] + \sum_{z \in V^*} \left[\mathrm{phs}'(z,u) - \mathrm{phs}(z,u)\right] \\ &\quad + \sum_{z \in V^*} \left[\mathrm{phs}'(z,v) - \mathrm{phs}(z,v)\right] + \sum_{z \in U^*} \left[\mathrm{phs}'(z,v) - \mathrm{phs}(z,v)\right]. \end{aligned} \tag{S45}$$

Let $z \in U^*$. By Proposition 1, $s_{\mathrm{LCA}(z,u)} = x \neq 0$, and thus $\mathrm{phs}(z,u) = 0$. In the modified tree, $s'_u = s_v = y \neq x = s_z$, and thus also $\mathrm{phs}'(z,u) = 0$. Next, since $s_v \neq s_z$, we have also $\mathrm{phs}(z,v) = 0$. In the modified tree, $s'_v = s_u = x = s_z$. In addition, since z and v are from different sides of the tree, their LCA is the root which is unmutated. Hence, $\mathrm{phs}'(z,v) = 1$. Now, let $z \in V^*$. By a similar argument, it follows that $\mathrm{phs}(z,v) = \mathrm{phs}'(z,v) = \mathrm{phs}(z,u) = 0$ and $\mathrm{phs}'(z,u) = 1$. Hence, (S45) reads

$$\sum_{z \in U^* \cup V^*} (\Delta(z,u) + \Delta(z,v)) = |U^*| + |V^*|,$$

from which (S44) follows. Plugging (S39), (S40), (S41), (S42) and (S43) and (S44) into (S38) proves the lemma.

□

I.6 Proof of Lemma 9 (probability distribution of $E_i(u)$)

Proof of Lemma 9. Let $e_u = E_i^{(u)}$. If $s_i^{(u)} = 0$, then $e_u = 0$ by definition. Next, consider the case $s_i^{(u)} > 0$. By Bayes' law,

$$\mathbb{P}\left[e_u = t \mid s_i^{(u)} > 0\right] = \frac{\mathbb{P}\left[e_u = t \wedge s_i^{(u)} > 0\right]}{\mathbb{P}\left[s_i^{(u)} > 0\right]}.$$

By definition, $A_i(u)$ is e_u edges above u. Let b be the parent of $A_i(u)$. Then b is unmutated and $A_i(u)$ is mutated. Since the depth of b is $d - e_u - 1$, we have

$$\mathbb{P}\left[e_u = t \wedge s_i^{(u)} > 0\right] = \alpha_d^{d-t-l} \cdot (1 - \alpha_d).$$

In addition,

$$\mathbb{P}\left[s_i^{(u)} > 0\right] = 1 - \mathbb{P}\left[s_i^{(u)} = 0\right] = 1 - \alpha_d^d = \rho.$$

The lemma follows by combining the above three equations. □

J Generation of Random Ground-Truth Tree Topologies

We generated ground-truth trees using the birth-death process implemented in the Cassiopeia package (Jones et al., 2020), specifying parameters for birth and death rates to model realistic lineage structures observed in single-cell tracing experiments. In our simulations, the time between cell divisions follows an exponential distribution with a birth rate initialized to 2. Tree lineages die at times drawn from an exponential distribution with a rate fixed at 0.75. Upon a cell division, its fitness changes with probability 50%. If the fitness changes, then the birth rate is multiplied by 1.1^z where z is drawn from a Normal distribution with mean 0.5 and standard deviation 0.25. The tree grows until the cell population reaches a predefined value, and then it terminates. The times are rescaled such that the end of the experiment is set to have time $\tau = 1$. Finally, a set of n leaves is subsampled. This subset induces an underlying ground-truth topology, denoted by $\mathcal{T}_{\mathrm{GT}}$.

bioRxiv preprint doi: https://doi.org/10.1101/2025.07.27.667007; this version posted July 5, 2026. The copyright holder for this preprint (which was not certified by peer review) is the author/funder, who has granted bioRxiv a license to display the preprint in perpetuity. It is made available under a CC-BY 4.0 International license.

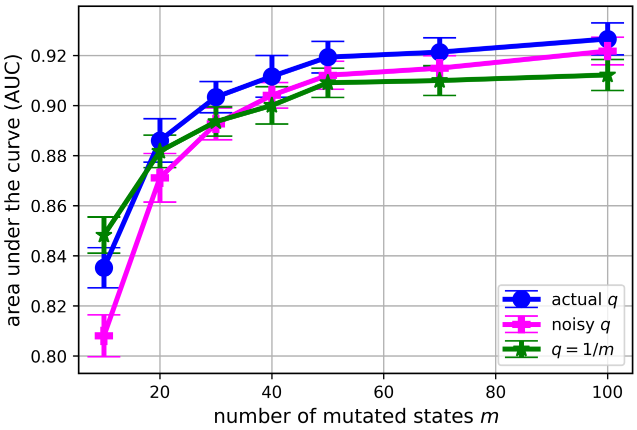

Figure S5: AUC values for cPHS with the true value of q, a noisy value of it, and with $q = 1/m$ that corresponds to uniform mutation probabilities ($q_j = 1/m$). The setting is the same as in Figure 3(right).

K Additional Simulation Results

As described in Section 3, our PHS-based test statistic requires the knowledge of the model parameter q. In our simulations, we assumed that this parameter is known for reasons explained in Appendix A.1. Moreover, empirically, our test statistic is not sensitive to the exact value of q. To illustrate this point, we calculated cPHS with three alternatives for the value of q: (i) the actual q; (ii) a noisy version of it, $q+\mathcal{N}(0, 0.3q)$, trimmed to the range $[0.1q, 1]$; and (iii) $q = 1/m$, which corresponds to uniform probability distribution of mutation states, namely $q_j = 1/m$ for $j = 1, \ldots, m$. The result, under the same setting of Figure 3, is depicted in Figure S5. As shown, the performance of cPHS is only slightly affected by an inaccurate estimation of q.

In the main text, Figures 2, 3, and 4 summarize the outcomes obtained from 7000 reconstructed trees evaluated under each parameter configuration. Figure S6 presents histograms that distinguish the counts of accurately versus inaccurately reconstructed trees within this sample of 7000. In the upper two panels of Figure S6, the distribution of accurate and inaccurate reconstructions is approximately balanced. In contrast, the lower panel exhibits a pronounced imbalance: the number of inaccurate reconstructions markedly exceeds that of accurate ones. This is due to the fact that the cutoff value ϵ in the lower panel is smaller.

Next, Figures S7, S8, S9, and S10 complement Fig. 1 of the main text by showing results for additional distance functions. Specifically, Figures S7 and S8 show the result for the parsimony distance function ($d = d_{\mathrm{P}}$) with the cutoff values $\epsilon = 0.03$ and $\epsilon = 0.1$, respectively. Figures S9 and S10 show results for the likelihood ($d = d_{\mathrm{L}}$) and triplets ($d = d_{\mathrm{tri}}$) distance functions, with the cutoff values $\epsilon = 0.05$ and $\epsilon = 1/5$, respectively. In all these results, the conclusion of Fig. 1 holds: only cPHS exhibits a good separation between the distribution of accurate and inaccurate trees. As discussed in the main text, these results imply that cPHS can tell whether the parsimony/likelihood of the reconstructed tree is close to the ground-truth one even better than parsimony and likelihood themselves.

Figures S11 and S12 complement Figures 2 and 3 of the main text, by showing results for the triplets and likelihood distance functions, with cutoff values $\epsilon = 0.1$ and $\epsilon = 0.03$, respectively. Figures S13, S15 and S16 complement Figures 3 and 4 of the main text but show results for additional cutoff values ϵ and additional ranges of parameters. Specifically, Figure S13 shows results for the same normalized RF function ($d = d_{\mathrm{RF}}$), but with the cutoff value $\epsilon = 1/4$. Next, Figure S14 shows the AUPRC (area under the Precision-Recall curve) as a function of the sequence length k, generalizing the results presented in the bottom panels of Figure 2. Finally, Figures S15 and S16 show results across ranges of three other model parameters: the number of mutations m, the mutation probability at a leaf ρ, and the number of observed leaves n. Across all these settings, the qualitative conclusions remain consistent with those presented in the main text.

==

L Analysis of the KP data set: set up

The KPTracer study deposited character matrices for 85 tumor samples (Yang et al., 2022). We selected the samples for analysis in two stages. First, we applied the quality-control criteria of Yang et al. (2022), which keep a sample if it has at least 5% of cells with a unique indel profile, at least 20% unsaturated target sites, and more than 100 cells.

bioRxiv preprint doi: https://doi.org/10.1101/2025.07.27.667007; this version posted July 5, 2026. The copyright holder for this preprint (which was not certified by peer review) is the author/funder, who has granted bioRxiv a license to display the preprint in perpetuity. It is made available under a CC-BY 4.0 International license.

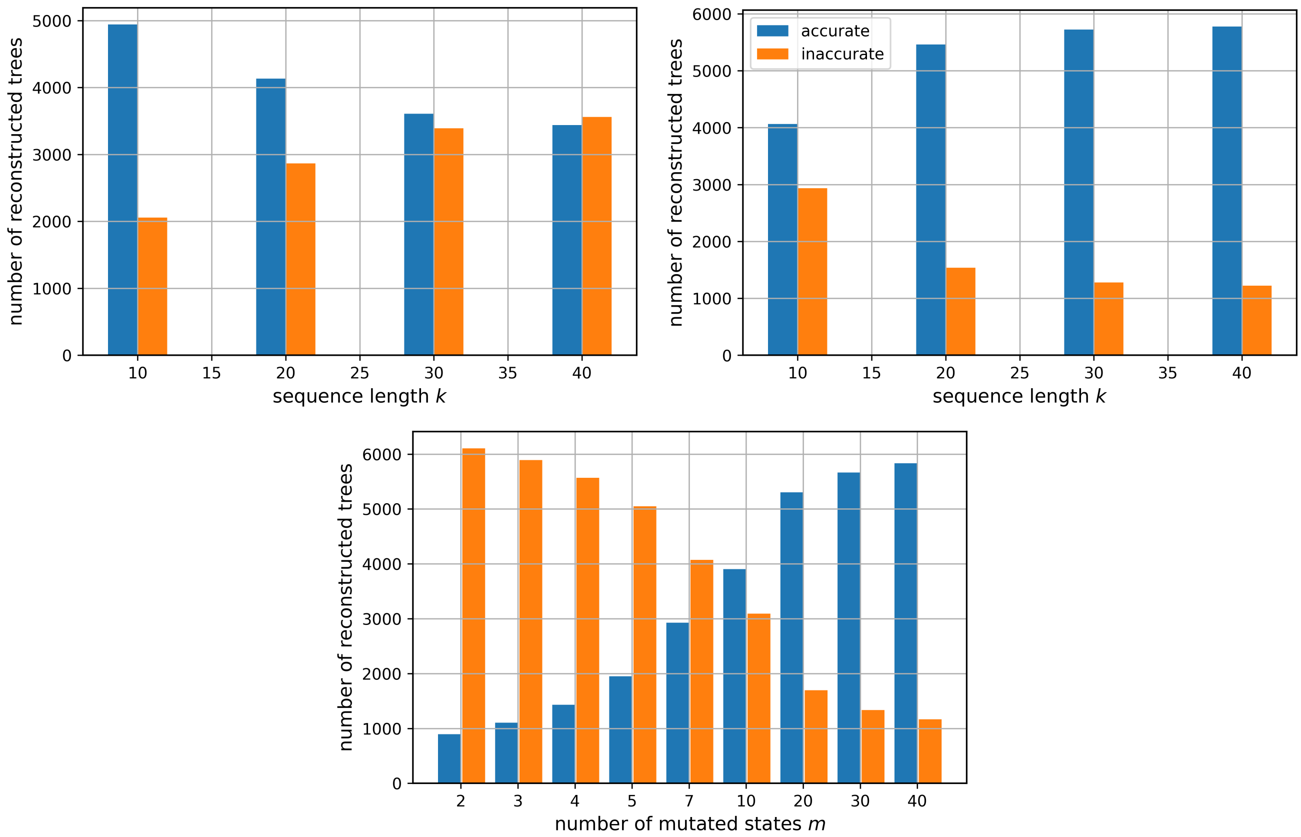

Figure S6: Histograms of accurate and inaccurate reconstructed trees, according to the hypothesis testing (3), as a function of sequence length k and number of mutated states m. Upper left panel: $d = d_{\mathrm{P}}$ and $\epsilon = 0.01$. Upper right panel: $d = d_{\mathrm{RF}}$ and $\epsilon = 1/7$. Lower panel: $d = d_{\mathrm{RF}}$ and $\epsilon = 1/7$.

For the composite samples (those combining cells from multiple lesions of an animal, labeled `_All` or `_Fam`), the indel and saturation statistics are not given in the deposit, so we applied only the cell-count criterion ($n > 100$). This stage removed 11 samples, leaving 74. Second, we reconstructed each remaining sample with all six reconstruction algorithms available in Cassiopeia package and removed any sample for which an algorithm failed to produce a tree (most often Neighbor Joining, which errors on some inputs), so that every analyzed sample has a complete set of six reconstructions. This removed a further 11 samples (6 individual tumors and 5 composites), leaving the **63 samples** analyzed in this work. The complete per-sample summary, with all recording parameters, is provided as a supplementary spreadsheet (`KPTracer_summary.xlsx`).

M Threshold calibration for the KP data

The use of cPHS score with the KP data requires a simulation study dedicated to calibrate the threshold for the parameters of the data. We generated ground-truth trees with the same birth-death process and CRISPR-Cas9 mutation overlay as in Section 4, spanning leaf counts $n \in \{100, 200, 300, 500, 1000, 2000, 3000\}$ character-site counts $k \in \{7, 10, 15, 20, 25, 30, 50, 70, 100\}$, mutation rates $\rho \in \{0.7, 0.9, 0.99\}$, and collision rates $q \in \{0.02, 0.1, 0.33\}$ with 100 repetitions per configuration. Trees were reconstructed with Cassiopeia Greedy, Maximum Cut, and SMJ. Each tree was labeled accurate or inaccurate by its normalized Robinson–Foulds distance to the ground truth, under cutoffs $\varepsilon \in \{0.10, 0.25, 0.50, 0.67\}$; for each configuration we found the optimal cPHS threshold (maximizing balanced accuracy) and evaluated the fixed thresholds $t \in \{0.05, 0.01, 10^{-3}, 10^{-4}, 10^{-5}, 10^{-6}\}$. The results of this analysis and the derived cutoffs (t), stratified by n, k, ρ, and q , are presented in Tables S1, S2, S3 and S4. In the range of n values that we analyzed we find that generally, for $k \leq 15$ or $q > 0.35$ the BA values are low, marking these regimes as a-priori difficult to infer accurate trees. Nevertheless, we select a threshold value for these cases (taking the highest scoring one) to enable our analysis of random instances in those regimes. For $k > 15$ the threshold tightens as ρ increases, reflecting that higher mutation rates push cPHS values downward.

bioRxiv preprint doi: https://doi.org/10.1101/2025.07.27.667007; this version posted July 5, 2026. The copyright holder for this preprint (which was not certified by peer review) is the author/funder, who has granted bioRxiv a license to display the preprint in perpetuity. It is made available under a CC-BY 4.0 International license.

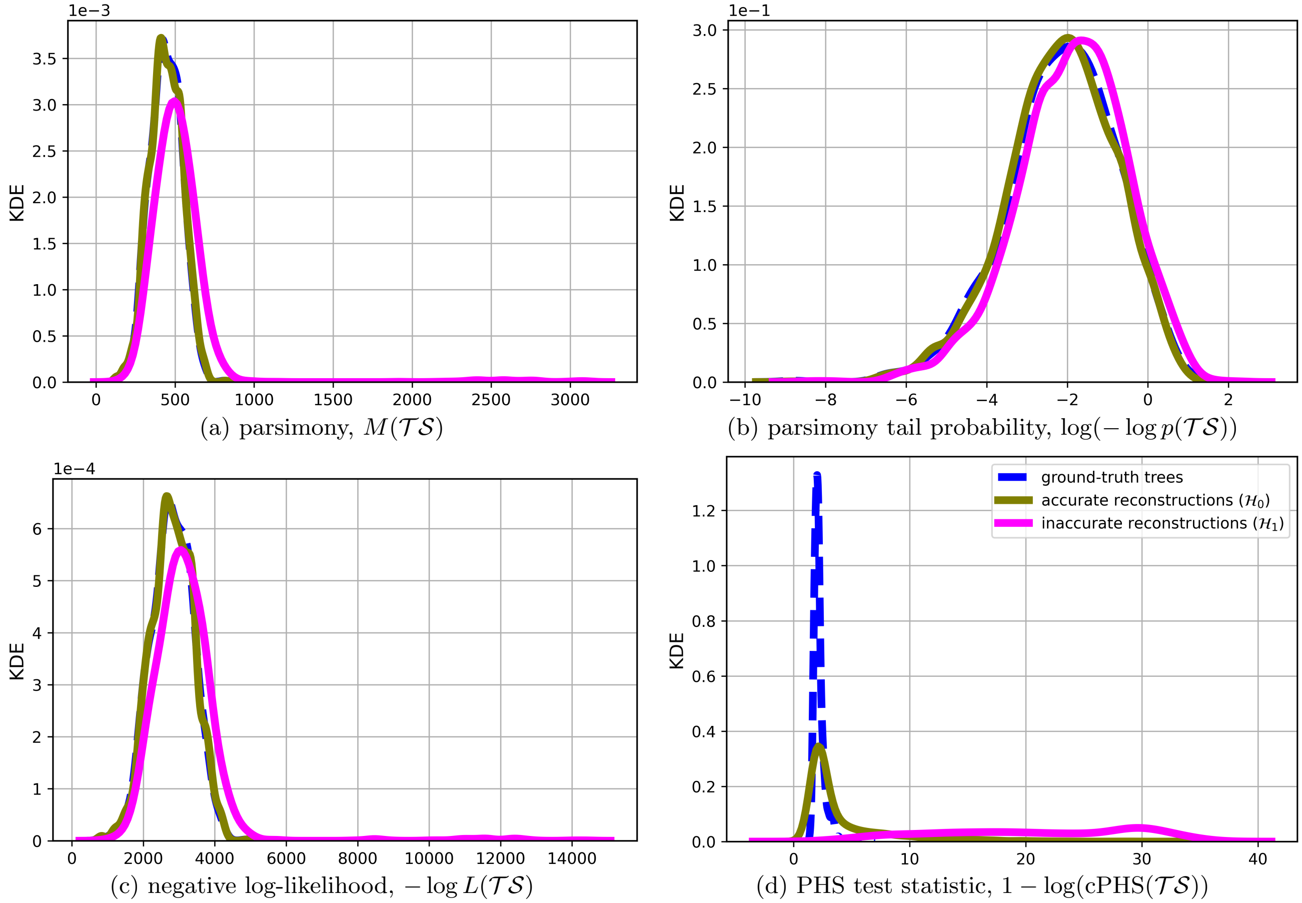

(a) parsimony, $M(\mathcal{TS})$

(b) parsimony tail probability, $\log(-\log p(\mathcal{TS}))$

(c) negative log-likelihood, $-\log L(\mathcal{TS})$

(d) PHS test statistic, $1 - \log(\mathrm{cPHS}(\mathcal{TS}))$

Figure S7: Kernel density estimates for the four accuracy measures, with the same setting as in Fig. 1, but with the parsimony as a distance function ($d = d_{\mathrm{P}}$) and $\epsilon = 0.03$.

The calibration results above are summarized into the operating thresholds used throughout the KP analysis (Table S5). The threshold t depends on both the number of recording sites k and the mutation probability ρ. For a given tumor we read off its respective threshold t from its measured k and ρ; since the KPTracer tumors have $\rho = 0.71$–0.999, the analysis uses the two higher-ρ columns.

bioRxiv preprint doi: https://doi.org/10.1101/2025.07.27.667007; this version posted July 5, 2026. The copyright holder for this preprint (which was not certified by peer review) is the author/funder, who has granted bioRxiv a license to display the preprint in perpetuity. It is made available under a CC-BY 4.0 International license.

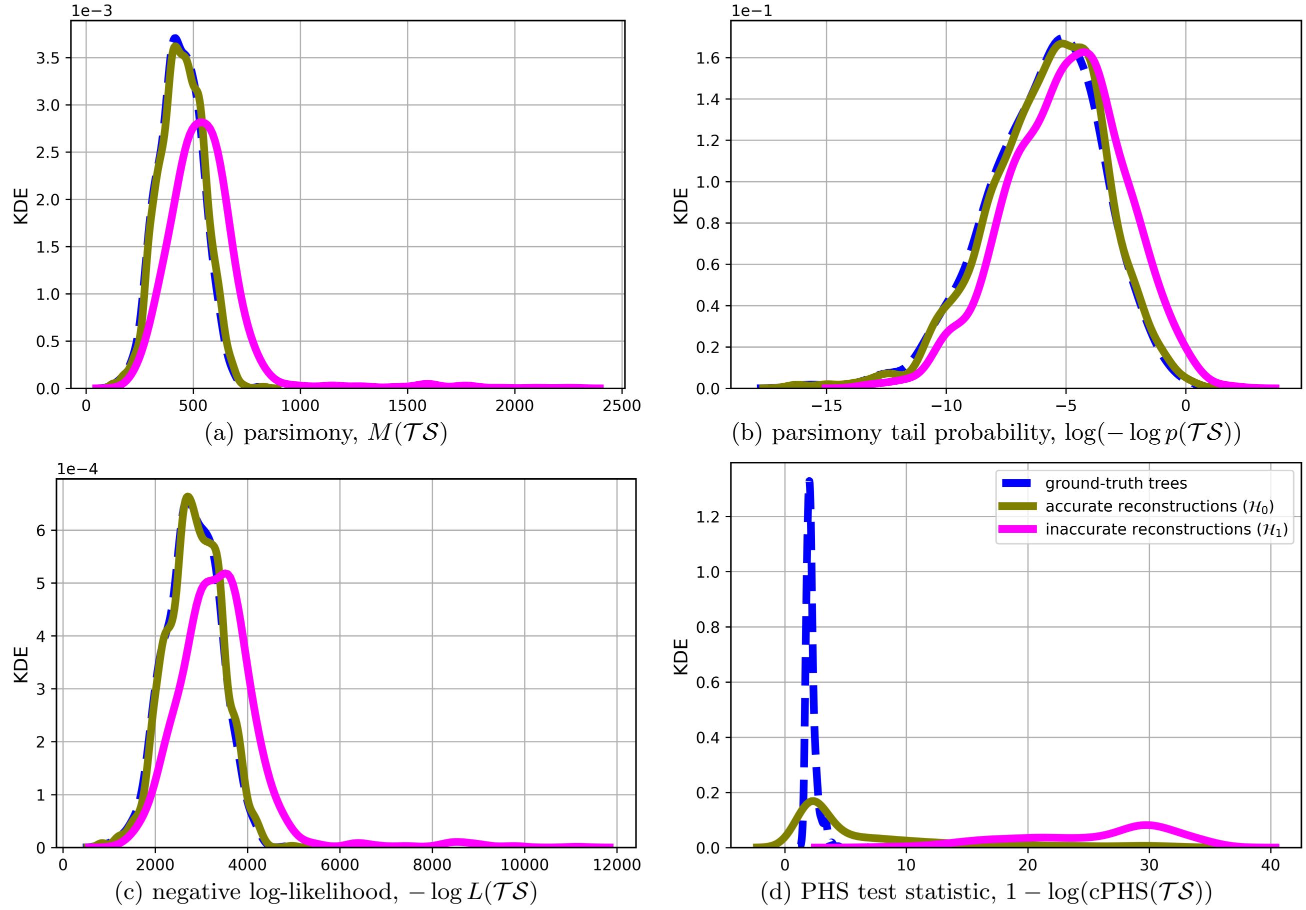

Figure S8: Kernel density estimates for the four accuracy measures, with the same setting as in Fig. 1, but with the parsimony as a distance function ($d = d_{\mathrm{P}}$) and $\epsilon = 0.1$.

bioRxiv preprint doi: https://doi.org/10.1101/2025.07.27.667007; this version posted July 5, 2026. The copyright holder for this preprint (which was not certified by peer review) is the author/funder, who has granted bioRxiv a license to display the preprint in perpetuity. It is made available under a CC-BY 4.0 International license.

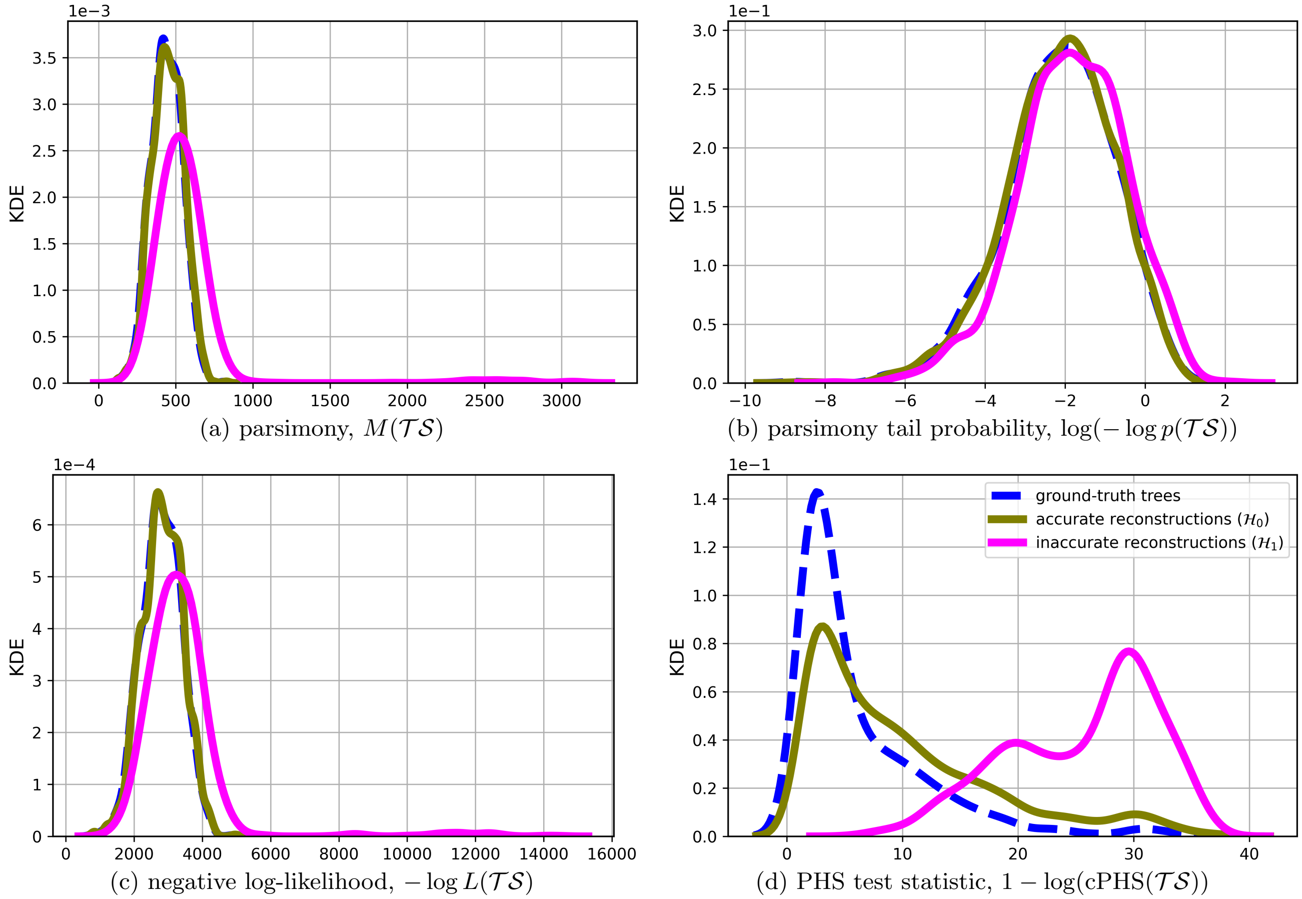

(a) parsimony, $M(\mathcal{TS})$

(b) parsimony tail probability, $\log(-\log p(\mathcal{TS}))$

(c) negative log-likelihood, $-\log L(\mathcal{TS})$

(d) PHS test statistic, $1 - \log(\mathrm{cPHS}(\mathcal{TS}))$

Figure S9: Kernel density estimates for the four accuracy measures, with the same setting as in Fig. 1, but with the likelihood as a distance function ($d = d_{\mathrm{L}}$) and $\epsilon = 0.05$.

bioRxiv preprint doi: https://doi.org/10.1101/2025.07.27.667007; this version posted July 5, 2026. The copyright holder for this preprint (which was not certified by peer review) is the author/funder, who has granted bioRxiv a license to display the preprint in perpetuity. It is made available under a CC-BY 4.0 International license.

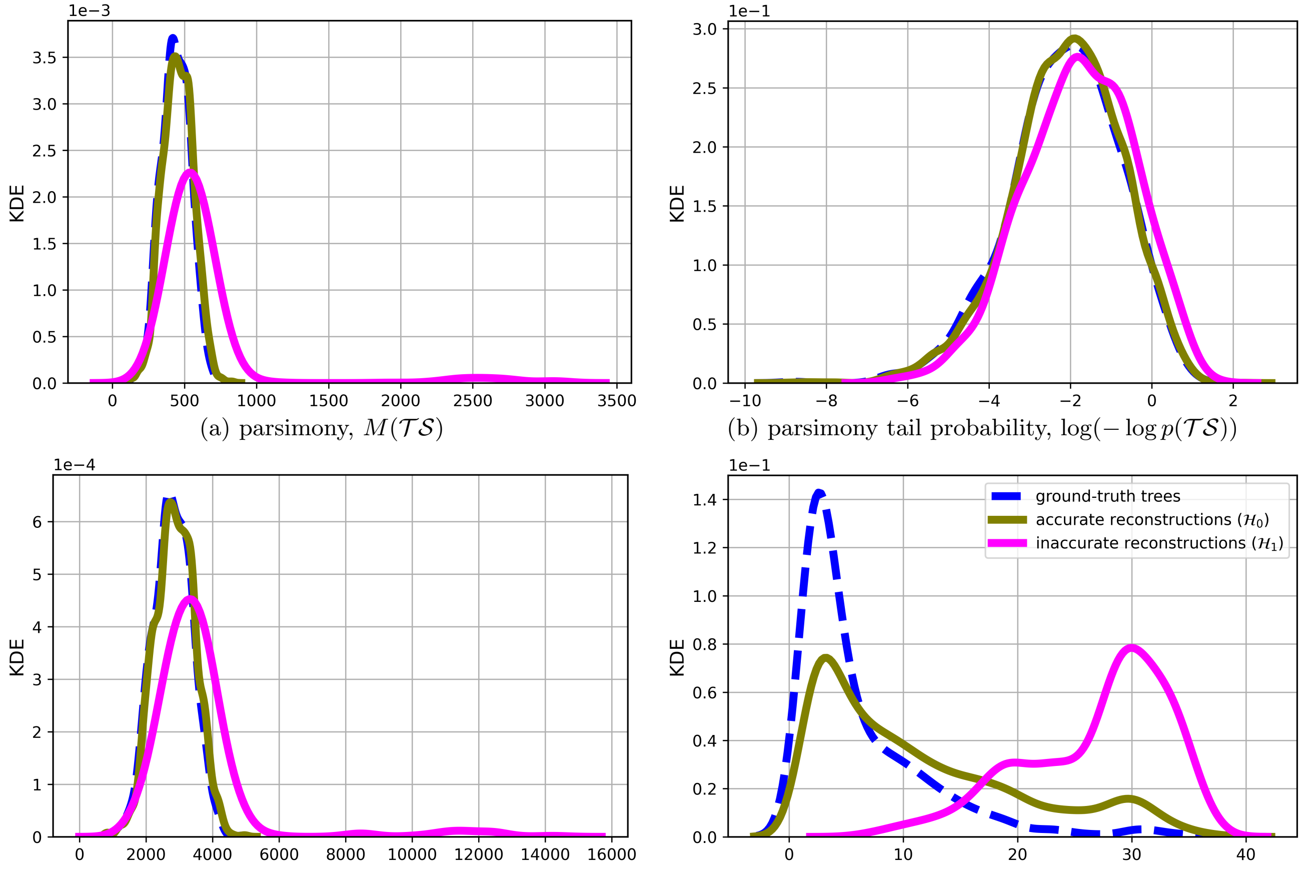

(a) parsimony, $M(\mathcal{TS})$

(b) parsimony tail probability, $\log(-\log p(\mathcal{TS}))$

(c) negative log-likelihood, $-\log L(\mathcal{TS})$

(d) PHS test statistic, $1 - \log(\mathrm{cPHS}(\mathcal{TS}))$

Figure S10: Kernel density estimates for the four accuracy measures, with the same setting as in Fig. 1, but with the triplets as a distance function ($d = d_{\mathrm{tri}}$) and $\epsilon = 1/5$.

bioRxiv preprint doi: https://doi.org/10.1101/2025.07.27.667007; this version posted July 5, 2026. The copyright holder for this preprint (which was not certified by peer review) is the author/funder, who has granted bioRxiv a license to display the preprint in perpetuity. It is made available under a CC-BY 4.0 International license.

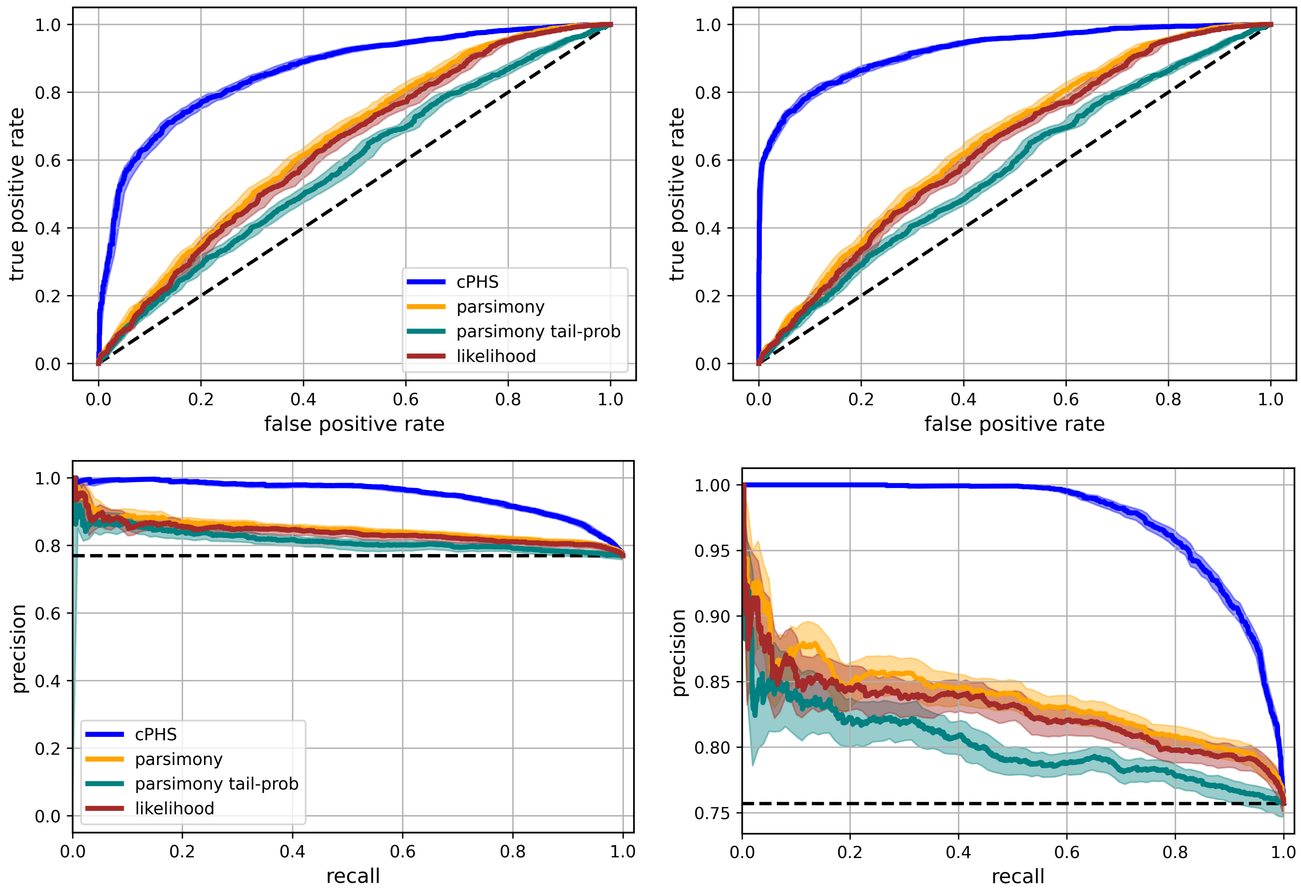

Figure S11: ROC (top) and Precision-Recall (bottom) curves with the same setting as in Fig. 2 except for the distance function. Left: triplets distance ($d = d_{\mathrm{tri}}$) with $\epsilon = 0.1$ (left panel). Right: likelihood distance ($d = d_{\mathrm{L}}$) with $\epsilon = 0.03$.

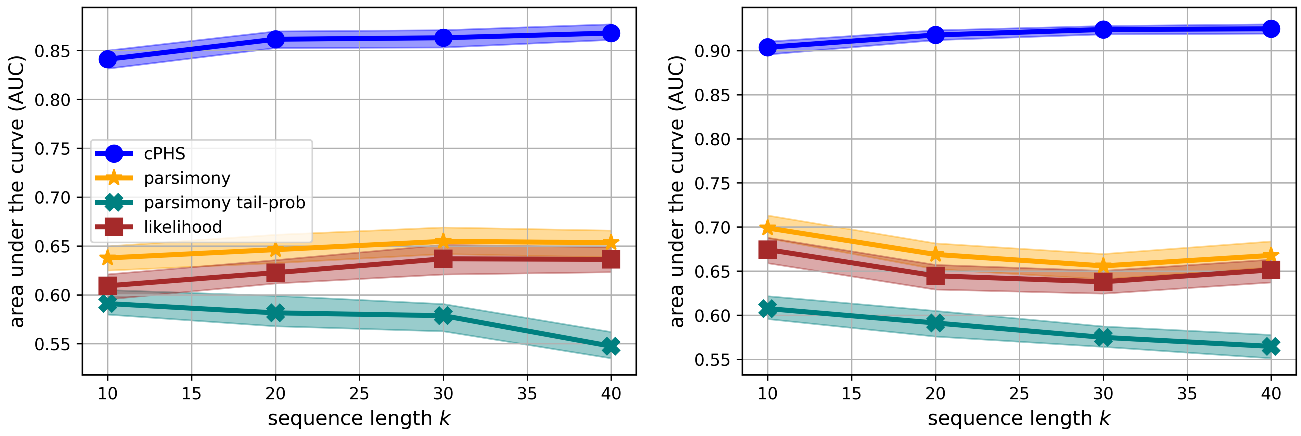

Figure S12: AUC values with the same setting as in Fig. 3(left), except for the distance function. Left: triplets distance ($d = d_{\mathrm{tri}}$) with $\epsilon = 1/5$. Right: likelihood distance ($d = d_{\mathrm{L}}$) with $\epsilon = 0.01$.

bioRxiv preprint doi: https://doi.org/10.1101/2025.07.27.667007; this version posted July 5, 2026. The copyright holder for this preprint (which was not certified by peer review) is the author/funder, who has granted bioRxiv a license to display the preprint in perpetuity. It is made available under a CC-BY 4.0 International license.

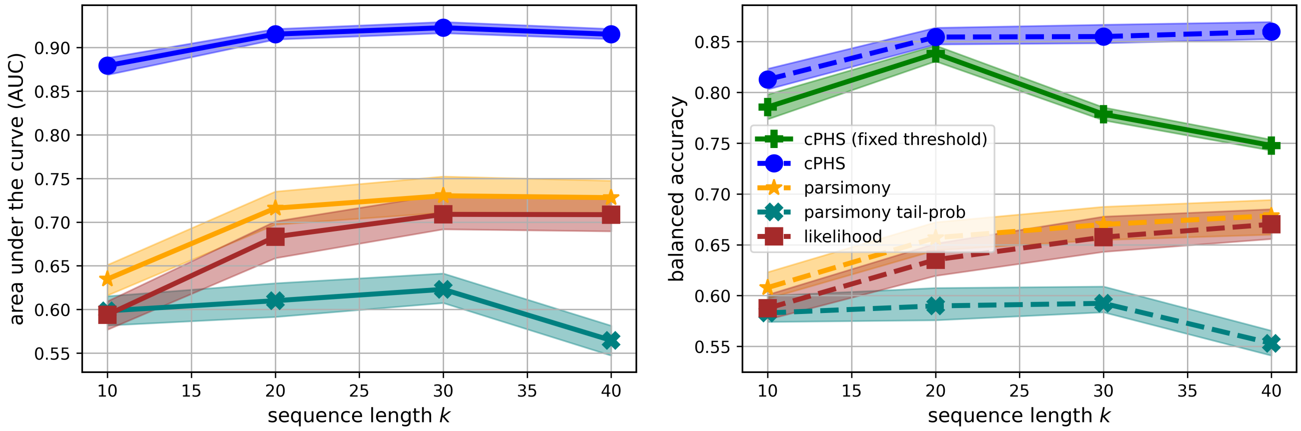

Figure S13: AUC and Balanced Accuracy values with the same setting as in the left panels of Figures. 3 and 4, except for the cutoff value which is $\epsilon = 1/4$.

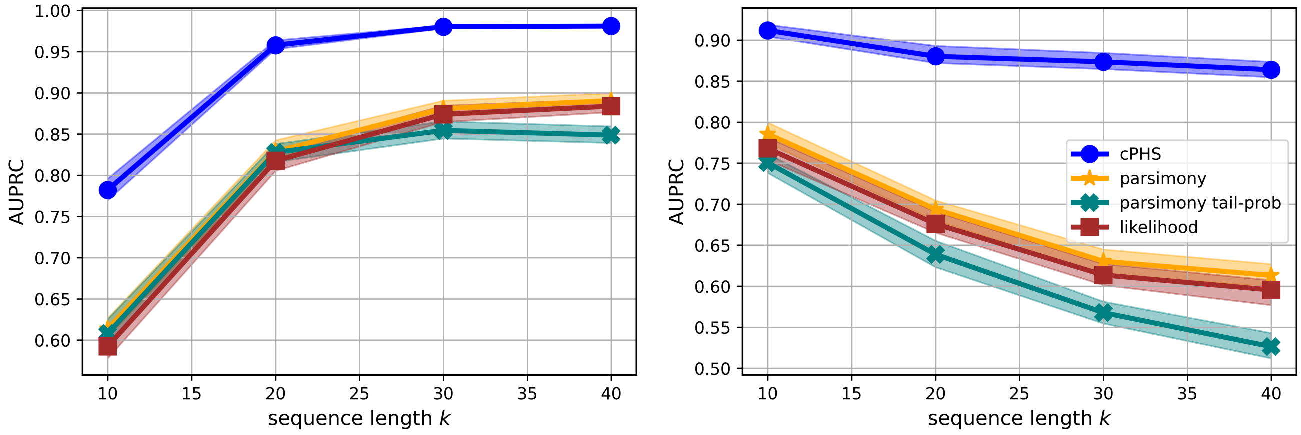

Figure S14: AUPRC (Area Under the Precision-Recall Curve) with the same setting as in the bottom left and right panels of Figure. 2, respectively. That is, left panel shows results for RF distance with $\epsilon = 1/7$, and right panels shows for parsimony distance with $\epsilon = 0.01$.

bioRxiv preprint doi: https://doi.org/10.1101/2025.07.27.667007; this version posted July 5, 2026. The copyright holder for this preprint (which was not certified by peer review) is the author/funder, who has granted bioRxiv a license to display the preprint in perpetuity. It is made available under a CC-BY 4.0 International license.

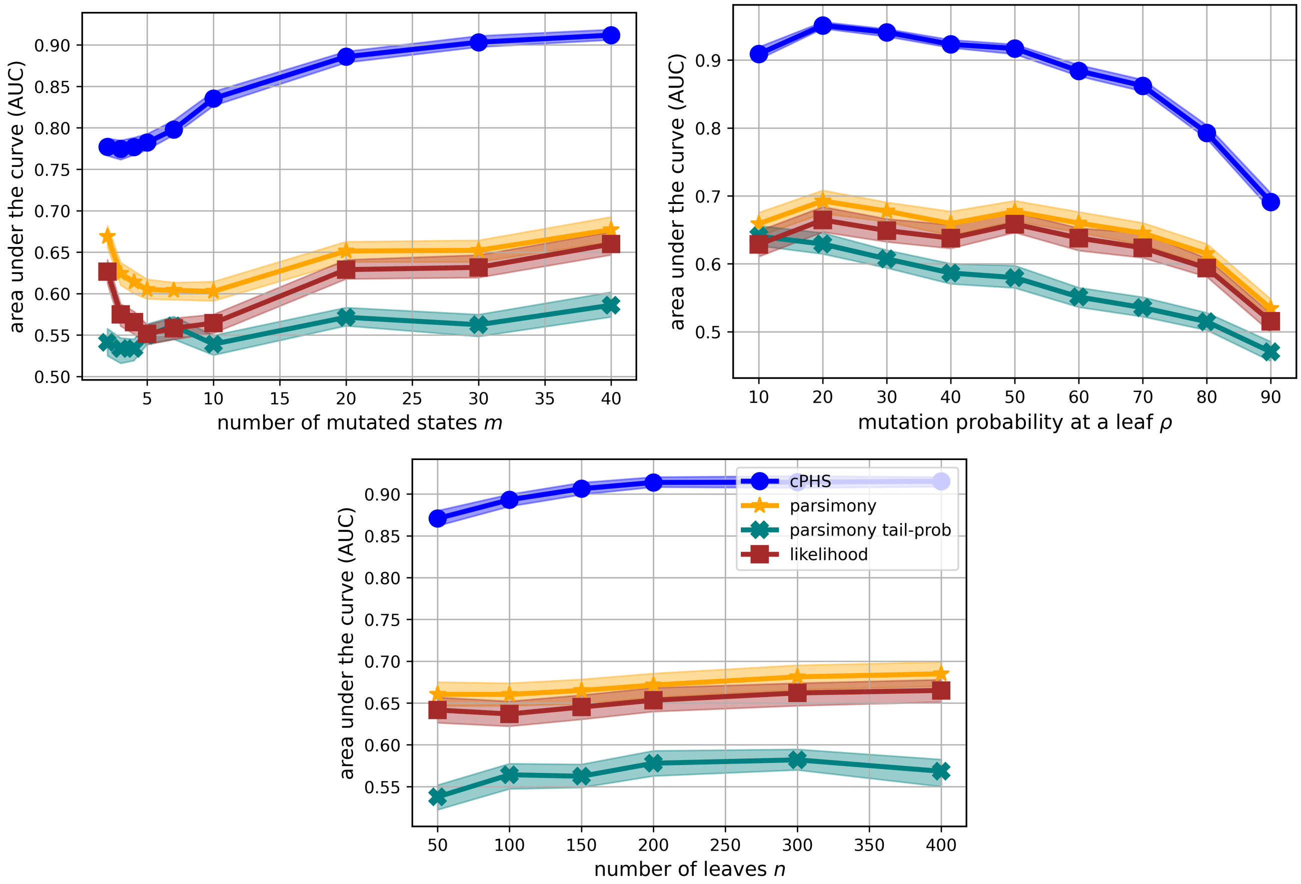

Figure S15: AUC values with the same setting as in Fig. 3, as a function of m (top left), ρ (top right) and n (bottom).

bioRxiv preprint doi: https://doi.org/10.1101/2025.07.27.667007; this version posted July 5, 2026. The copyright holder for this preprint (which was not certified by peer review) is the author/funder, who has granted bioRxiv a license to display the preprint in perpetuity. It is made available under a CC-BY 4.0 International license.

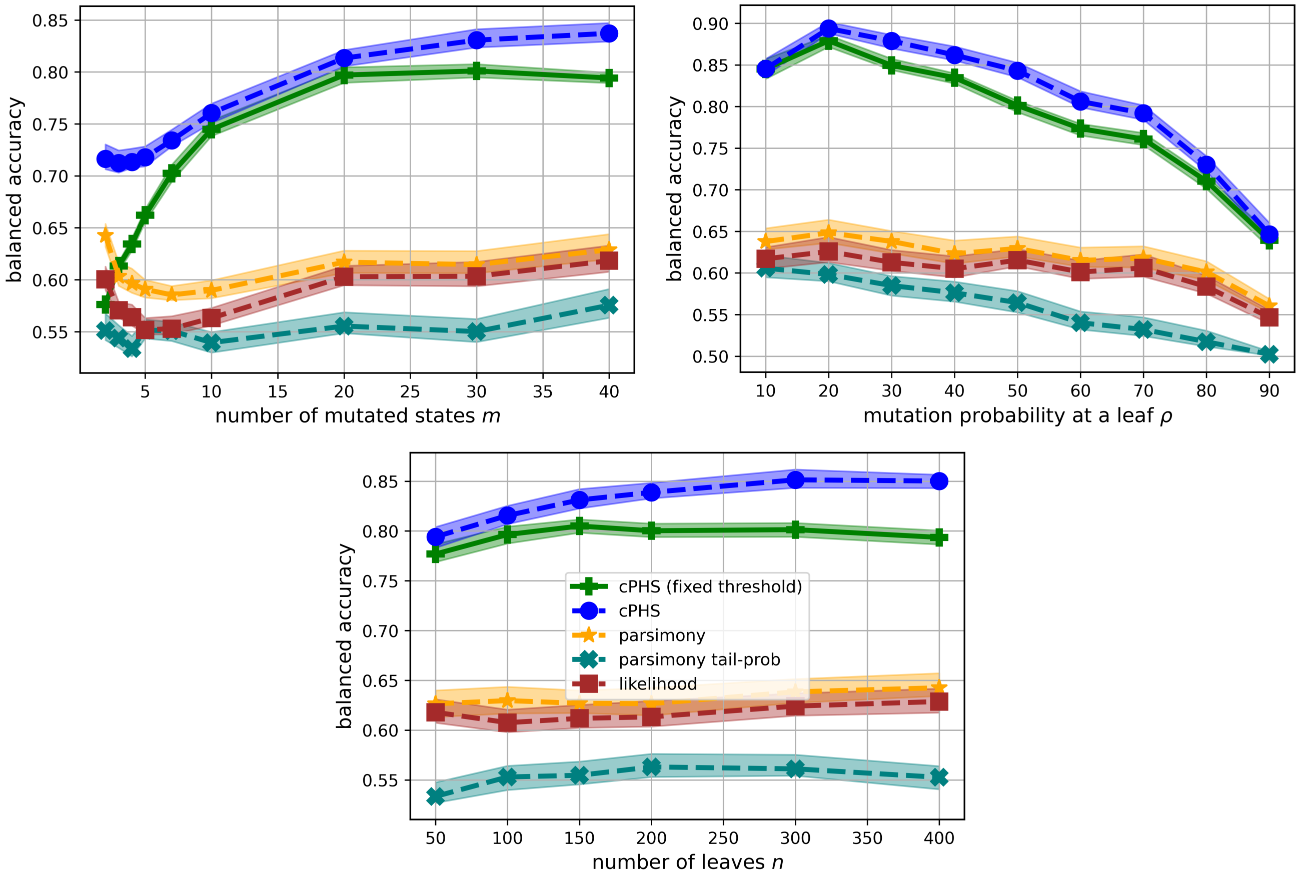

Figure S16: Balanced accuracy of the test statistics with the same setting as in Fig. 4, as a function of m (top left), ρ (top right) and n (bottom).

Table S1: Threshold calibration simulation results at $\rho = 0.5$. For each (n, k) configuration, the table shows the balanced accuracy (BA) achieved by six fixed thresholds (best per row in **bold**, where a tree is accepted if cPHS $\geq t$), and the median cPHS and mean normalized RF distance across all reconstructed trees in that configuration. The ε column is the accuracy cutoff used to label a tree accurate (RF ratio $\leq \varepsilon$) or inaccurate; it is fixed by k-regime rather than tuned for performance: $\varepsilon = 0.50$ (the standard cutoff) for $k \geq 15$, relaxed to $\varepsilon = 0.67$ for $k = 10$, where reconstructions are too poor for $\varepsilon = 0.50$ to yield enough accurate trees. The results show that for $k < 15$ no fixed threshold gives reliable balanced accuracy, even under the relaxed cutoff $\varepsilon = 0.67$: BA stays near chance (0.5) across all thresholds, confirming that the test lacks power in this regime. Reliable separation emerges only for $k > 15$.

n	k	ε	BA(0.05)	BA(0.01)	BA(10^{-3})	BA(10^{-4})	BA(10^{-5})	BA(10^{-6})	Med. cPHS	Mean RF
100	10	0.67	**0.63**	0.59	0.56	0.50	0.50	0.50	2.5×10^{-1}	0.65
200	10	0.67	**0.58**	0.55	0.53	0.51	0.49	0.50	1.7×10^{-1}	0.67
300	10	0.67	**0.54**	**0.54**	0.52	0.52	0.50	0.51	1.4×10^{-1}	0.69
500	10	0.67	**0.52**	0.51	0.50	**0.52**	0.51	0.51	6.6×10^{-2}	0.70
1000	10	0.67	0.50	0.52	**0.53**	0.50	0.48	0.48	2.0×10^{-2}	0.72
100	15	0.50	**0.59**	0.56	0.51	0.49	0.49	0.49	1.1×10^{-1}	0.55
200	15	0.50	**0.69**	0.65	0.59	0.57	0.53	0.51	4.2×10^{-2}	0.57
300	15	0.50	**0.67**	0.64	0.66	0.61	0.57	0.54	1.2×10^{-2}	0.59
500	15	0.67	0.58	**0.60**	0.57	**0.60**	0.57	0.57	5.6×10^{-3}	0.60
100	20	0.50	0.64	**0.66**	0.60	0.62	0.59	0.55	3.2×10^{-2}	0.47
200	20	0.50	0.63	0.63	**0.65**	0.62	0.58	0.56	1.2×10^{-2}	0.50
300	20	0.50	0.58	0.59	**0.61**	0.59	0.57	0.54	4.8×10^{-3}	0.50
500	20	0.50	**0.61**	0.60	**0.61**	0.57	0.55	0.53	1.6×10^{-3}	0.52
1000	20	0.50	**0.51**	**0.51**	0.50	0.49	0.49	0.49	7.0×10^{-5}	0.54
100	25	0.50	0.65	0.67	0.66	**0.68**	0.64	0.57	2.0×10^{-2}	0.43
200	25	0.50	0.64	**0.68**	0.66	0.66	0.62	0.56	8.7×10^{-3}	0.43
300	25	0.50	0.58	0.62	0.62	0.62	**0.63**	0.62	5.8×10^{-4}	0.45
500	25	0.50	0.55	0.59	**0.63**	0.57	0.56	0.56	2.6×10^{-4}	0.46
100	30	0.50	0.61	0.66	0.73	**0.77**	0.73	0.74	2.6×10^{-3}	0.38
200	30	0.50	0.64	0.69	**0.75**	0.72	0.73	0.74	5.7×10^{-4}	0.39
300	30	0.50	0.61	0.65	0.65	0.67	**0.71**	**0.71**	1.6×10^{-4}	0.41
500	30	0.50	0.53	0.56	**0.60**	0.55	0.55	0.48	3.3×10^{-5}	0.42
1000	30	0.50	0.58	0.59	0.60	**0.61**	0.60	0.56	2.6×10^{-6}	0.43

bioRxiv preprint doi: https://doi.org/10.1101/2025.07.27.667007; this version posted July 5, 2026. The copyright holder for this preprint (which was not certified by peer review) is the author/funder, who has granted bioRxiv a license to display the preprint in perpetuity. It is made available under a CC-BY 4.0 International license.

bioRxiv preprint doi: https://doi.org/10.1101/2025.07.27.667007; this version posted July 5, 2026. The copyright holder for this preprint (which was not certified by peer review) is the author/funder, who has granted bioRxiv a license to display the preprint in perpetuity. It is made available under a CC-BY 4.0 International license.

Table S2: Effect of mutation probability ρ on threshold performance ($n = 100$, $\varepsilon = 0.50$). As ρ increases, absolute cPHS values drop by orders of magnitude (median cPHS column), shifting the optimal threshold downward. Balanced accuracy (BA) is shown for five fixed thresholds (best per row in **bold**, tree accepted if cPHS $\geq t$). The adopted thresholds are marked with $\bullet$: $t = 10^{-3}$ for $k = 16$–24 and $t = 10^{-4}$ for $k \geq 25$. The $k = 15$ block is shown for reference only; it is flagged in the analysis, and consistent with Table S1 no fixed threshold separates reliably there.

k	ρ	BA(0.05)	BA(0.01)	BA(10^{-3})	BA(10^{-4})	BA(10^{-5})	Med. cPHS
15	0.5	**0.59**	0.56	0.51	0.49	0.49	1.1×10^{-1}
15	0.7	**0.64**	0.62	0.63	0.61	0.57	3.2×10^{-2}
15	0.9	0.67	0.69	**0.73**	0.72	0.70	3.7×10^{-3}
15	0.99	0.62	0.64	0.65	0.66	**0.69**	4.2×10^{-5}
20	0.5	0.64	**0.66**	$0.60^\bullet$	0.62	0.59	3.2×10^{-2}
20	0.7	0.69	0.71	$0.73^\bullet$	**0.77**	0.69	4.6×10^{-3}
20	0.9	0.62	0.63	$0.61^\bullet$	0.66	**0.68**	3.5×10^{-5}
20	0.99	0.61	0.66	$0.70^\bullet$	0.70	**0.72**	2.3×10^{-5}
25	0.5	0.65	0.67	0.66	**0.68**$^\bullet$	0.64	2.0×10^{-2}
25	0.7	0.66	0.71	0.77	$0.82^\bullet$	**0.86**	1.4×10^{-3}
25	0.9	0.61	0.66	0.69	$0.75^\bullet$	**0.78**	1.8×10^{-5}
25	0.99	0.49	0.52	0.57	$0.60^\bullet$	**0.62**	6.6×10^{-10}
30	0.5	0.61	0.66	0.73	**0.77**$^\bullet$	0.73	2.6×10^{-3}
30	0.7	0.61	0.65	0.72	$0.78^\bullet$	**0.83**	4.7×10^{-4}
30	0.9	0.57	0.59	0.63	**0.67**$^\bullet$	0.56	2.3×10^{-8}
30	0.99	0.50	0.52	0.53	$0.54^\bullet$	**0.58**	4.2×10^{-10}

N Comparison to Likelihood and Parsimony

The consistency between cPHS scores and that of parsimony and likelihood can be summarized as a best-method concordance. Table S6 reports, for each pair of metrics, the fraction of the 42 reliable tumors in which the two metrics select the same best method. cPHS and likelihood agree most often (36%), cPHS and parsimony slightly less (31%), and parsimony and likelihood least (24%); all three coincide in only 10% of tumors (4 of 42, all on Cassiopeia Greedy). The cPHS–likelihood disagreement is systematic rather than noise: when cPHS prefers a method other than the one likelihood favors, likelihood selects SMJ in two-thirds of those cases (18 of 27), reflecting that SMJ optimizes an objective close to likelihood during construction. The metrics thus capture overlapping but distinct notions of tree quality, consistent with the moderate rank correlations reported above.

O Factors influencing cPHS performance in KP data

Considering the objective of rejecting random trees - the collision probability q is the strongest predictor of cPHS separation (Spearman $\rho = -0.54$, $p = 1.4 \times 10^{-6}$), stronger than the effective homoplasy rate $\lambda \times q$ ($\rho = -0.50$) or λ alone ($\rho = -0.31$); see Figure S19. Separation is strong for $q < 0.35$ (mean 7.6 orders of magnitude), degrades for $0.35 \leq q < 0.75$ (mean 3.2), and collapses for $q \geq 0.75$. Practically, q can be estimated directly from character-state frequencies, so cPHS applicability can be assessed directly.

Focusing on the 42 tumors with $q < 0.35$ and $k > 15$, 14 had no inferred tree passing the cPHS threshold. We examined tumor-level recording parameters and data-quality indicators (number of cells n, sites k, collision probability q, mutation probability ρ, percent missing data, unique alleles per site, percent unique cell profiles, percent unsaturated sites, and the derived quantities k/n, $k/\log n$, $k \cdot (1 - q)$, and $k \cdot \log(1/q)$) and correlated each with the best $\log_{10}$(cPHS) per tumor. The correlations are summarized in Table S8.

The dominant correlate is tumor size ($r = -0.77$ between n and best $\log_{10}$(cPHS)): passing tumors have median 300 cells vs. 1,145 for non-passing, and all 4 tumors with $n > 2{,}000$ and 8 of 10 with $n > 1{,}000$ fall in the no-pass category. The rationale is the need for increased number of recording sites for larger number of leaves, which has been shown to be quadratic in nature. The ratio k/n is thus the single most discriminative predictor ($r = +0.70$): all 14 non-passing tumors have $k/n < 0.05$, and above this value every tumor has at least one method passing. The

bioRxiv preprint doi: https://doi.org/10.1101/2025.07.27.667007; this version posted July 5, 2026. The copyright holder for this preprint (which was not certified by peer review) is the author/funder, who has granted bioRxiv a license to display the preprint in perpetuity. It is made available under a CC-BY 4.0 International license.

Table S3: Effect of mutation probability ρ on threshold performance at $n = 500$, $\varepsilon = 0.50$ (compare Table S2 for $n = 100$). The same threshold ranking holds, but balanced accuracy is consistently lower, reflecting the increased difficulty of classifying larger trees. Balanced accuracy (BA) is shown for five fixed thresholds (best per row in **bold**, tree accepted if cPHS $\geq t$). The adopted thresholds are marked with $\bullet$: $t = 10^{-3}$ for $k = 16$–24 and $t = 10^{-4}$ for $k \geq 25$. The $k = 15$ block is shown for reference only; it is flagged in the analysis.

k	ρ	BA(0.05)	BA(0.01)	BA(10^{-3})	BA(10^{-4})	BA(10^{-5})	Med. cPHS
15	0.5	0.59	**0.77**	0.71	0.63	0.57	5.6×10^{-3}
15	0.7	**0.60**	**0.60**	0.57	0.55	0.55	1.2×10^{-3}
15	0.9	0.59	**0.64**	0.61	0.61	**0.64**	2.3×10^{-4}
15	0.99	**0.92**	0.90	0.85	0.82	0.79	1.9×10^{-7}
20	0.5	**0.61**	0.60	**0.61**$^\bullet$	0.57	0.55	1.6×10^{-3}
20	0.7	0.60	0.63	0.62$^\bullet$	**0.64**	0.61	2.5×10^{-4}
20	0.9	0.57	0.59	0.58$^\bullet$	0.60	**0.61**	1.5×10^{-5}
20	0.99	0.49	0.54	0.54$^\bullet$	0.56	**0.60**	7.6×10^{-8}
25	0.5	0.55	0.59	**0.63**	0.57$^\bullet$	0.56	2.6×10^{-4}
25	0.7	0.59	0.63	0.66	0.71$^\bullet$	**0.75**	1.6×10^{-6}
25	0.9	0.55	0.57	0.61	0.65$^\bullet$	**0.67**	5.0×10^{-8}
25	0.99	0.51	0.53	0.56	0.59$^\bullet$	**0.60**	1.2×10^{-8}
30	0.5	0.53	0.56	**0.60**	0.55$^\bullet$	0.55	3.3×10^{-5}
30	0.7	0.58	0.61	0.63	0.69$^\bullet$	**0.70**	1.3×10^{-6}
30	0.9	0.55	0.57	0.60	0.62$^\bullet$	**0.65**	5.6×10^{-10}
30	0.99	0.52	0.54	0.56	0.56$^\bullet$	**0.58**	3.8×10^{-11}

positive correlation of q with passing ($r = +0.70$) reflects the opposite effect, since higher q inflates cPHS toward the threshold by raising chance homoplasy, and is itself a size confound, since larger tumors have lower q.

Simulation-based validation. Simulating trees across the KP (n, q) regime ($n = 100$–2,000, $q \approx 0.02$–0.33, $k = 20, 30$, $\rho = 0.9$) reproduces the empirical pattern: pass rates fall with n and rise with q, and the KP tumors that fail cluster in the high-n, low-q region where simulations also predict low pass rates. This supports the interpretation that cPHS failures in the reliable set are driven by insufficient k relative to n.

P Degradation experiments for KP data: NNI perturbation and data degragation

We assessed cPHS sensitivity to topological degradation by using controlled NNI perturbation of the reconstructed trees.

P.1 NNI perturbation

To evaluate how cPHS responds to controlled degradation of tree topology, we performed Nearest Neighbor Interchange (NNI) perturbation experiments. Starting from the reconstructed tree for each tumor, we applied random NNI moves that swap subtrees across internal edges, progressively distorting the topology while holding the character data fixed. After each perturbation level we measured cPHS, parsimony, likelihood, as well as Robinson–Foulds (RF) distance and triplets-correct distance to the original tree.

Edge-normalized perturbation. A fair comparison of perturbation effects between reconstruction methods requires accounting for differences in tree topology. Cassiopeia Greedy produces shallow trees with many polytomies (few internal edges, large fan-out), whereas Shared Mutation Joining (SMJ) produces fully binary trees (many internal edges, all bifurcating). Applying the same number of NNI moves to both tree types produces unequal effective perturbation: each move on a Cassiopeia tree swaps larger subtrees, and so induces greater topological change, than the same move on a binary SMJ tree. To make the two comparable, we normalized the number of NNI moves by the number of internal edges in each tree. Perturbation levels were set at $0, 5, 10, 25, 50, 100, 150, 200\%$ of

bioRxiv preprint doi: https://doi.org/10.1101/2025.07.27.667007; this version posted July 5, 2026. The copyright holder for this preprint (which was not certified by peer review) is the author/funder, who has granted bioRxiv a license to display the preprint in perpetuity. It is made available under a CC-BY 4.0 International license.

Table S4: Effect of collision probability q on threshold performance ($n = 100$, $\varepsilon = 0.50$). Higher q shifts cPHS values upward (median cPHS column), making strict thresholds less effective. Balanced accuracy (BA) is shown for five fixed thresholds (best per row in **bold**, tree accepted if cPHS $\geq t$). At $q = 0.33$ (the upper boundary of the low-q regime), the adopted threshold $t = 10^{-4}$ ($\bullet$) achieves only moderate balanced accuracy, while $t = 0.05$ becomes the best-performing threshold. This independently validates the partitioning of tumors at $q = 0.35$: the calibrated thresholds are effective for low-q tumors but would not be appropriate for high-q tumors.

k	q	m	ρ	BA(0.05)	BA(0.01)	BA(10^{-3})	BA(10^{-4})	BA(10^{-5})	Med. cPHS
25	0.02	50	0.5	0.65	0.67	0.66	**0.68**$^\bullet$	0.64	2.0×10^{-2}
25	0.02	50	0.9	0.61	0.66	0.69	0.75$^\bullet$	**0.78**	1.8×10^{-5}
25	0.10	10	0.5	**0.65**	**0.65**	0.63	0.55$^\bullet$	0.51	7.2×10^{-2}
25	0.10	10	0.9	0.69	0.68	**0.72**	0.69$^\bullet$	0.66	1.3×10^{-3}
25	0.33	3	0.5	**0.64**	0.59	0.55	0.51$^\bullet$	0.50	3.1×10^{-1}
25	0.33	3	0.9	**0.69**	0.63	0.56	0.54$^\bullet$	0.52	2.3×10^{-1}
30	0.02	50	0.5	0.61	0.66	0.73	**0.77**$^\bullet$	0.73	2.6×10^{-3}
30	0.02	50	0.9	0.57	0.59	0.63	**0.67**$^\bullet$	0.56	2.3×10^{-8}
30	0.10	10	0.5	**0.74**	**0.74**	0.70	0.65$^\bullet$	0.59	7.2×10^{-2}
30	0.10	10	0.9	0.67	0.67	**0.71**	0.68$^\bullet$	0.67	1.3×10^{-3}
30	0.33	3	0.5	**0.63**	0.63	0.57	0.54$^\bullet$	0.52	1.9×10^{-1}
30	0.33	3	0.9	**0.66**	0.63	0.61	0.56$^\bullet$	0.53	1.4×10^{-1}

Table S5: Adopted cPHS thresholds t as a function of the number of recording sites k and the mutation probability ρ. Tumors with $k \leq 15$ are flagged for insufficient power. Since the KPTracer tumors have $\rho = 0.71$–0.999, the analysis uses the middle and right columns.

	$\rho = 0.5$–0.6	$\rho = 0.7$–0.9	$\rho = 0.99$
$k \leq 15$	flagged (insufficient power)		
$k = 16$–24	$t = 0.05$	$t = 10^{-3}$	$t = 10^{-4}$
$k \geq 25$	$t = 10^{-3}$	$t = 10^{-4}$	$t = 10^{-4}$

internal edges. At 100%, both methods reach comparable RF distances to the original tree (Cassiopeia 0.59 ± 0.04, SMJ 0.57 ± 0.03), confirming that the normalization produces equivalent effective perturbation.

Tumors analyzed. NNI results were computed for 34 of the reliable low-q tumors, each perturbed with 20 replicates per level. All 34 contribute to both the cPHS pass-rate and the continuous-metric curves. A tumor is counted as passing at a given perturbation level if a majority of its replicate trees pass cPHS.

Results. Figures S20 show that the cPHS pass rate declines with perturbation, pooled across tumors and averaged per tumor respectively, confirming that cPHS detects topological degradation. The two standard tree-comparison metrics move consistently with it: RF distance to the original rises monotonically and triplets-correct falls monotonically as perturbation increases, for both methods , with the Cassiopeia Greedy algorithm showing wider tumor-to-tumor spread than SMJ in both. Figure S21 shows all four continuous metrics (parsimony, RF, triplets, likelihood) degrading monotonically and consistently for both methods, validating the edge-normalized scheme. Per-tumor parsimony, likelihood, RF and triplets trajectories for representative tumors are given in Figure S20, where the tight replicate agreement confirms that the degradation is reproducible regardless of the specific random sequence of moves. Table S10 aggregates the effect over the 48 low-q tumors: the cPHS pass rate falls from 41.7% (intact) to 16.7% at 50% NNI, with median cPHS, parsimony, and likelihood all degrading monotonically while random trees remain fully separated (0% pass).

The same perturbation analysis also confirms why the low-k tumors are flagged. Table S9 shows cPHS for NNI-perturbed Cassiopeia Greedy trees of the six flagged tumors ($k \leq 15$): even trees with 25–50% of their topology scrambled routinely retain cPHS values that would clear any reasonable threshold, so cPHS cannot distinguish a good tree from a badly perturbed one at these site counts. This is in direct contrast to the $k \geq 25$ tumors, where 50%-perturbed trees almost never pass.

bioRxiv preprint doi: https://doi.org/10.1101/2025.07.27.667007; this version posted July 5, 2026. The copyright holder for this preprint (which was not certified by peer review) is the author/funder, who has granted bioRxiv a license to display the preprint in perpetuity. It is made available under a CC-BY 4.0 International license.

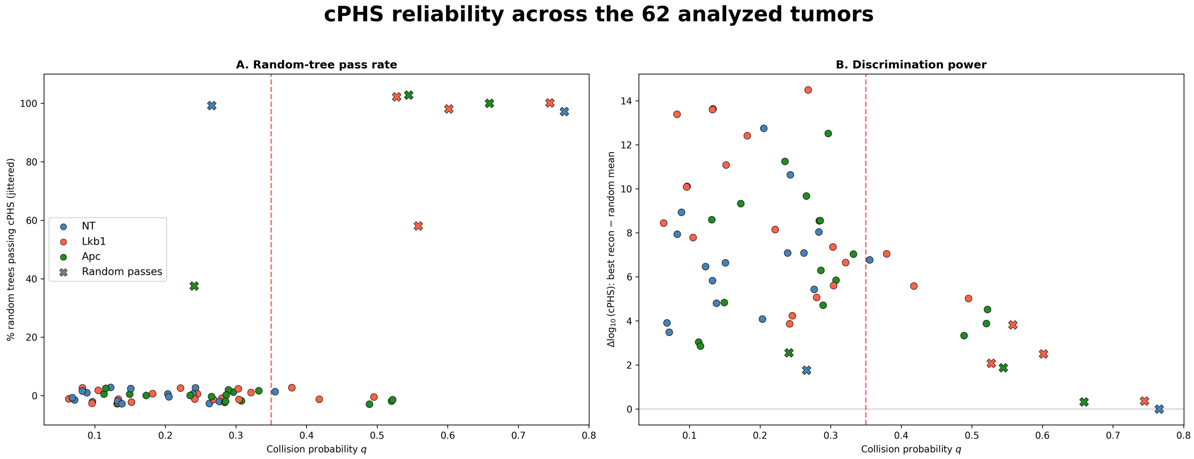

Figure S17: cPHS reliability across the 63 analyzed KPTracer tumors, evaluated at the calibrated thresholds ($t = 10^{-3}$ for $k \leq 24$, $t = 10^{-4}$ for $k \geq 25$). (A) Fraction of random trees passing cPHS vs. collision probability q; markers colored by genotype, X marks tumors with at least one random pass. Random passes occur in 10 tumors, 7 at high q ($q \geq 0.35$, where chance homoplasy lifts cPHS) and 3 at low q but few sites ($k \leq 15$, where the test lacks power). (B) Discrimination power, the $\log_{10}$ separation between the best reconstruction's cPHS and the random-tree mean, vs. q; large at low q, collapsing as q increases. Red dashed line: $q = 0.35$. No random tree passes in the 42 reliable tumors ($k > 15$, $q < 0.35$).

Table S6: Best-method concordance across the 42 reliable low-q tumors. Each off-diagonal entry is the fraction of tumors in which the row and column metrics select the same best reconstruction method. All three metrics agree on the single best method in only 10% of tumors (4 of 42), reflecting that each rewards a different property of the tree.

	cPHS	Parsimony	Likelihood
cPHS	—	31%	36%
Parsimony	31%	—	24%
Likelihood	36%	24%	—

Appendix References

Day, William HE, David S Johnson, and David Sankoff (1986). "The computational complexity of inferring rooted phylogenies by parsimony". In: *Mathematical biosciences* 81.1, pp. 33–42.

Felsenstein, Joseph (2004). *Inferring Phylogenies*. Sinauer Associates. Sunderland, Massachusetts.

Fitch, Walter M (1977). "On the problem of discovering the most parsimonious tree". In: *The American Naturalist* 111.978, pp. 223–257.

Jones, Matthew G et al. (2020). "Inference of single-cell phylogenies from lineage tracing data using Cassiopeia". In: *Genome biology* 21.92.

Prillo, Sebastian et al. (2026). "ConvexML: Fast and accurate branch length estimation under irreversible mutation models, illustrated through applications to CRISPR/Cas9-based lineage tracing". In: *Systematic Biology* 75.1, pp. 115–134.

Sankoff, David (1975). "Minimal mutation trees of sequences". In: *SIAM Journal on Applied Mathematics* 28.1, pp. 35–42.

Sashittal, Palash et al. (2023). "Startle: a star homoplasy approach for crispr-cas9 lineage tracing". In: *Cell Systems* 14.12, pp. 1113–1121.

bioRxiv preprint doi: https://doi.org/10.1101/2025.07.27.667007; this version posted July 5, 2026. The copyright holder for this preprint (which was not certified by peer review) is the author/funder, who has granted bioRxiv a license to display the preprint in perpetuity. It is made available under a CC-BY 4.0 International license.

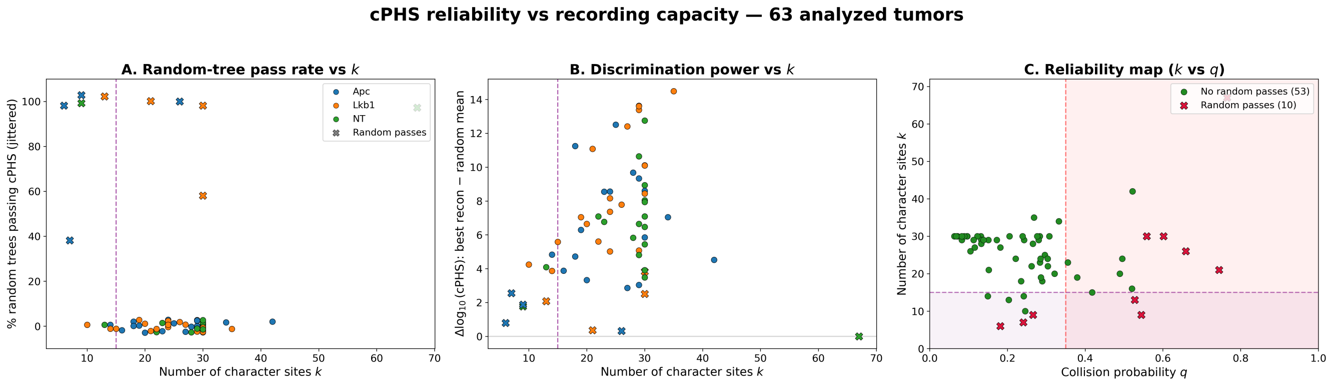

Figure S18: cPHS reliability as a function of recording capacity across the 63 analyzed KPTracer tumors, evaluated at the calibrated thresholds ($t = 10^{-3}$ for $k \leq 24$, $t = 10^{-4}$ for $k \geq 25$). (A) Fraction of random trees passing cPHS vs. number of character sites k; markers colored by genotype, X marks tumors with at least one random pass, and the purple dashed line marks $k = 15$. (B) Discrimination power, the $\log_{10}$ separation between the best reconstruction's cPHS and the random-tree mean, vs. k. (C) Reliability map of k vs. collision probability q: tumors with no random passes (green) versus at least one random pass (red X), with the reliable region ($k > 15$, $q < 0.35$) unshaded and the low-k ($k \leq 15$) and high-q ($q \geq 0.35$) zones shaded. Random passes concentrate in the low-k and high-q zones; the reliable region is free of them.

Table S7: Number of tumors (out of the 42 reliable) in which each method achieves the best score under each metric. Cassiopeia Greedy is preferred most often by cPHS and parsimony, whereas SMJ is preferred most often by likelihood, reflecting that the three metrics reward different properties.

Method	Best cPHS	Best parsimony	Best likelihood
Cassiopeia Greedy	18	24	14
Maximum Cut	8	17	3
SMJ	11	1	25
Neighbor Joining	5	0	0
Spectral Greedy	0	0	0
Spectral	0	0	0

Seidel, Sophie and Tanja Stadler (2022). "TiDeTree: a Bayesian phylogenetic framework to estimate single-cell trees and population dynamic parameters from genetic lineage tracing data". In: *Proceedings of the Royal Society B* 289.1986, p. 20221844.

Yang, Dian et al. (2022). "Lineage tracing reveals the phylodynamics, plasticity, and paths of tumor evolution". In: *Cell* 185.11, pp. 1905–1923.

bioRxiv preprint doi: https://doi.org/10.1101/2025.07.27.667007; this version posted July 5, 2026. The copyright holder for this preprint (which was not certified by peer review) is the author/funder, who has granted bioRxiv a license to display the preprint in perpetuity. It is made available under a CC-BY 4.0 International license.

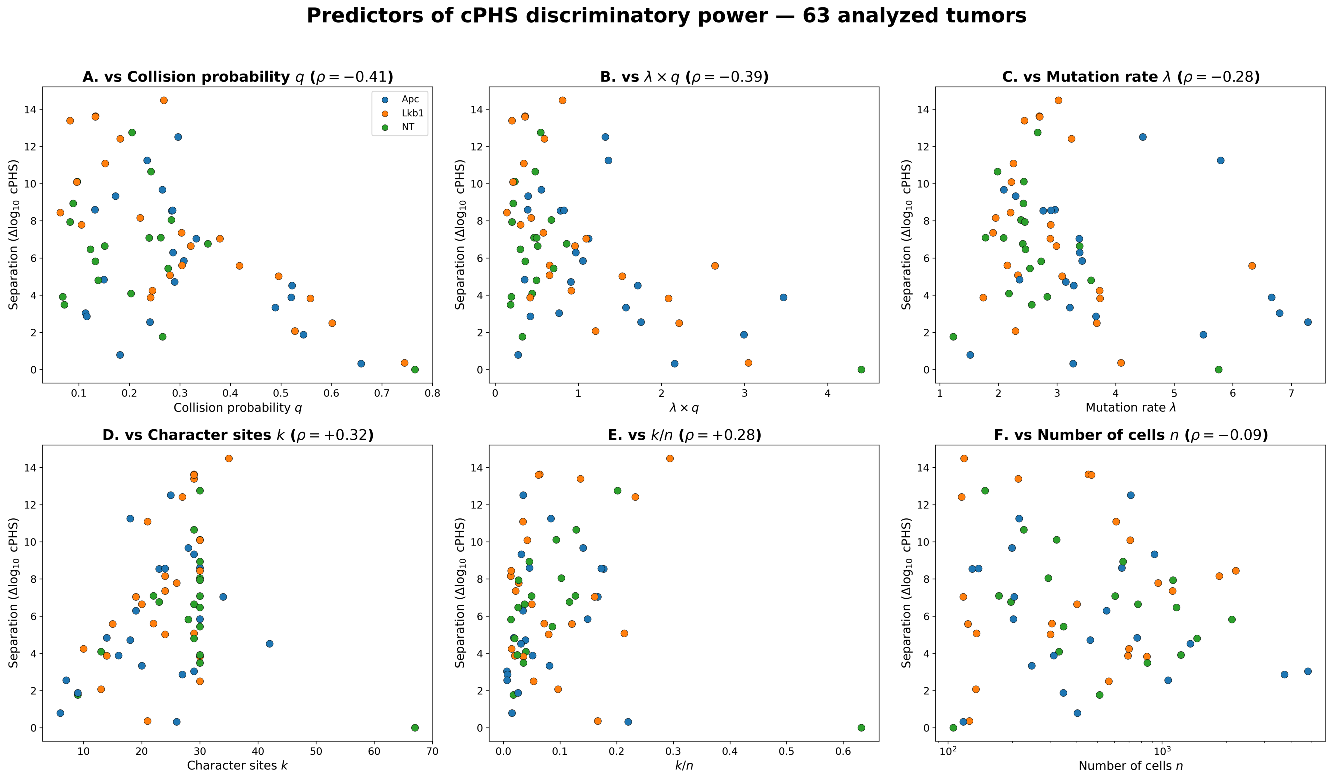

Figure S19: Predictors of cPHS discriminatory power across the 63 analyzed KPTracer tumors. Separation is the $\log_{10}$ gap between the best reconstruction's cPHS and the random-tree mean; points colored by genotype. Collision probability q is the strongest single predictor (A, $\rho = -0.41$), followed by $\lambda \times q$ (B, $\rho = -0.39$), with weaker dependence on the mutation rate λ (C, $\rho = -0.28$), character sites k (D, $\rho = +0.32$), the ratio k/n (E, $\rho = +0.28$), and number of cells n (F, $\rho = -0.09$, n.s.).

Table S8: Key quality indicators for the 42 reliable low-q tumors, stratified by cPHS outcome. Spearman r is the correlation with $\log_{10}$(best cPHS) across all 42. * $p < 0.05$; ** $p < 0.01$.

Indicator	Passes (28)	No pass (14)	Spearman r	p
n (cells)	300	1,145	−0.77	$< 10^{-4}$**
k (character sites)	28.5	29.5	−0.31	0.048*
q (collision prob.)	0.267	0.114	+0.70	$< 10^{-4}$**
k/n	0.090	0.026	+0.70	$< 10^{-4}$**
% missing data	21.3	15.9	+0.34	0.027*
Unique alleles/site	11.7	22.9	−0.71	$< 10^{-4}$**
% unique cells	61.8	53.8	+0.13	0.43
% unsaturated sites	93.9	100.0	−0.29	0.082
$k \cdot (1-q)$	20.8	25.9	−0.63	$< 10^{-4}$**
$k \cdot \log(1/q)$	37.3	61.9	−0.68	$< 10^{-4}$**

bioRxiv preprint doi: https://doi.org/10.1101/2025.07.27.667007; this version posted July 5, 2026. The copyright holder for this preprint (which was not certified by peer review) is the author/funder, who has granted bioRxiv a license to display the preprint in perpetuity. It is made available under a CC-BY 4.0 International license.

Table S9: cPHS of perturbed Cassiopeia Greedy trees for the 6 flagged tumors ($k \leq 15$). Even trees with 25–50% of their topology scrambled by NNI moves routinely achieve cPHS values that would pass any reasonable threshold, confirming that cPHS lacks discriminatory power for these tumors. For comparison, in tumors with $k \geq 25$ the 50%-perturbed trees almost never pass.

Tumor	k	Best recon	5% NNI	10% NNI	25% NNI	50% NNI
3430_NT_T2	9	3.6e-01	6.6e-02	2.3e-02	6.9e-02	2.1e-02
3454_Lkb1_T3	10	1.0e+00	1.0e+00	8.2e-01	1.2e-01	1.7e-04
3508_Apc_T2_Fam	7	2.8e-01	1.6e-01	6.6e-02	1.5e-01	1.4e-01
3433_NT_T2	13	1.0e+00	6.3e-01	1.6e-01	5.2e-01	4.3e-01
3510_Apc_T2	14	6.5e-06	7.4e-07	7.4e-07	7.4e-07	4.4e-08
3732_Lkb1_T2	14	7.1e-02	6.7e-02	5.7e-02	8.8e-04	3.2e-02

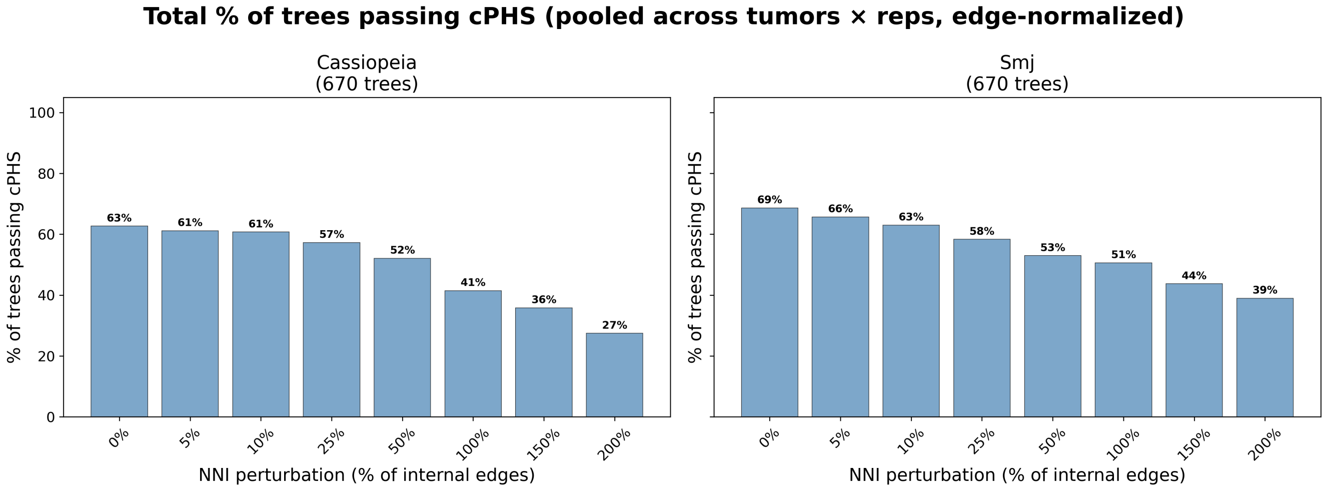

Figure S20: Total percentage of trees passing cPHS at each NNI perturbation level (edge-normalized), pooling all tumors and replicates. Left: Cassiopeia; right: SMJ. Pass rates decline with increasing perturbation.

Table S10: Effect of NNI perturbation on reconstruction quality, restricted to the 48 low-q tumors ($q < 0.35$) where cPHS is fully reliable. Starting from the Cassiopeia Greedy tree, increasing fractions of NNI moves are applied. All metrics degrade monotonically with perturbation level. cPHS median and mean $\log_{10}$ show the typical cPHS value; Pars vs rand and Lik vs rand show the mean percent improvement over random trees.

Level	N	cPHS pass	cPHS median	Mean $\log_{10}$	Pars vs rand	Lik vs rand
Cassiopeia (intact)	48	41.7%	2.24e-03	−4.72	82.8%	69%
Cass + 5% NNI	48	39.6%	1.60e-03	−4.84	81.2%	66%
Cass + 10% NNI	48	35.4%	2.95e-04	−5.08	79.6%	64%
Cass + 25% NNI	48	22.9%	2.83e-05	−6.06	76.1%	60%
Cass + 50% NNI	48	16.7%	1.22e-05	−6.31	70.8%	55%
Random	240	0.0%	5.43e-13	−10.71	-0.0%	0%

bioRxiv preprint doi: https://doi.org/10.1101/2025.07.27.667007; this version posted July 5, 2026. The copyright holder for this preprint (which was not certified by peer review) is the author/funder, who has granted bioRxiv a license to display the preprint in perpetuity. It is made available under a CC-BY 4.0 International license.

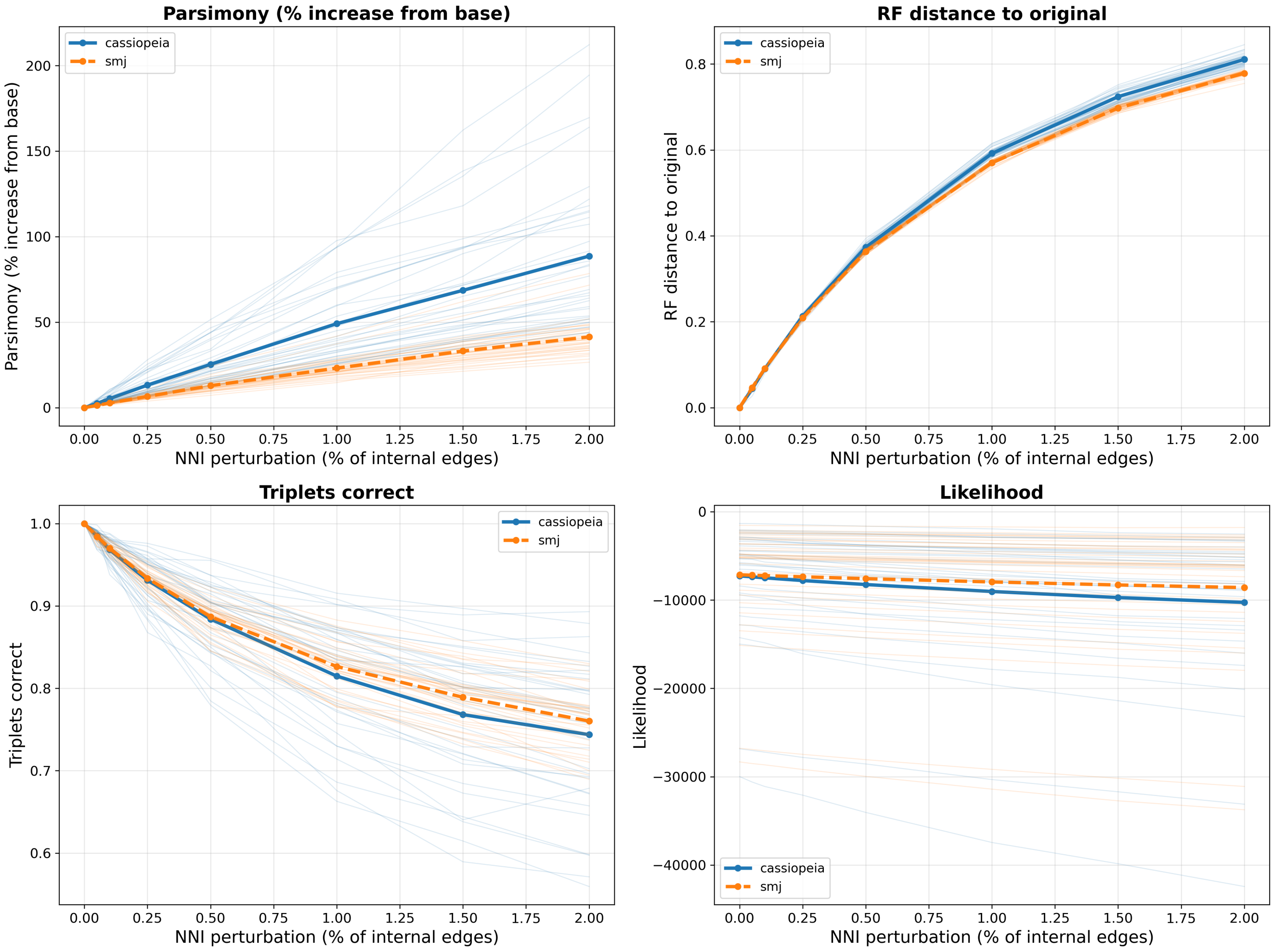

Figure S21: Degradation of quality metrics under edge-normalized NNI perturbation, across all 34 tumors. Top left: parsimony (% increase from base). Top right: RF distance. Bottom left: triplets correct. Bottom right: likelihood. Faint lines: individual tumor means; bold lines: grand mean. Blue: Cassiopeia; orange: SMJ.
